# Reductive coupling of ketones and nitriles at a geometrically constrained silanide

**DOI:** 10.1039/d6qi01961g

**Published:** 2026-10-29

**Authors:** Pamela Adienes Benzan Lantigua, María L. G. Sansores-Paredes, Martin Lutz, Marc-Etienne Moret

**Affiliations:** a Organic Chemistry and Catalysis, Institute for Sustainable and Circular Chemistry, Faculty of Science, Utrecht University 3584 CG Utrecht The Netherlands m.moret@uu.nl; b Department of Chemistry and Applied Biosciences, ETH Zürich CH-8093 Zürich Switzerland; c Structural Biochemistry, Bijvoet Centre for Biomolecular Research, Faculty of Science, Utrecht University 3584 CG Utrecht The Netherlands

## Abstract

While silanides ([R_3_Si^−^]) are best known for their nucleophilic reactivity, examples have recently been shown to display biphilic reactivity akin to neutral Si(ii) compounds. In this context, we report on the reactivity of silanide [K(18-crown-6)][tmimSi] (tmimH_3_ = tris(3-methylindol-2-yl)methane) with carbonyl compounds and nitriles. The unusual reductive cross-coupling of a ketone and a nitrile result in the formation of 5-membered silacycles. The ketone and the nitrile coupling partners are each able to form homocoupling products, but at a slower rate. DFT calculations support an Umpolung mechanism involving initial condensation of the ketone and silanide to form an anionic oxasilirane – an analogue of which can be isolated for benzaldehyde – that readily opens at the Si–C bond to insert a nitrile molecule.

## Abstract 2

A silanide incorporated in a bicyclic tris-indolide structure mediates the reductive cross-coupling of ketones and nitriles to form anionic heterocycles.

## Graphical abstract

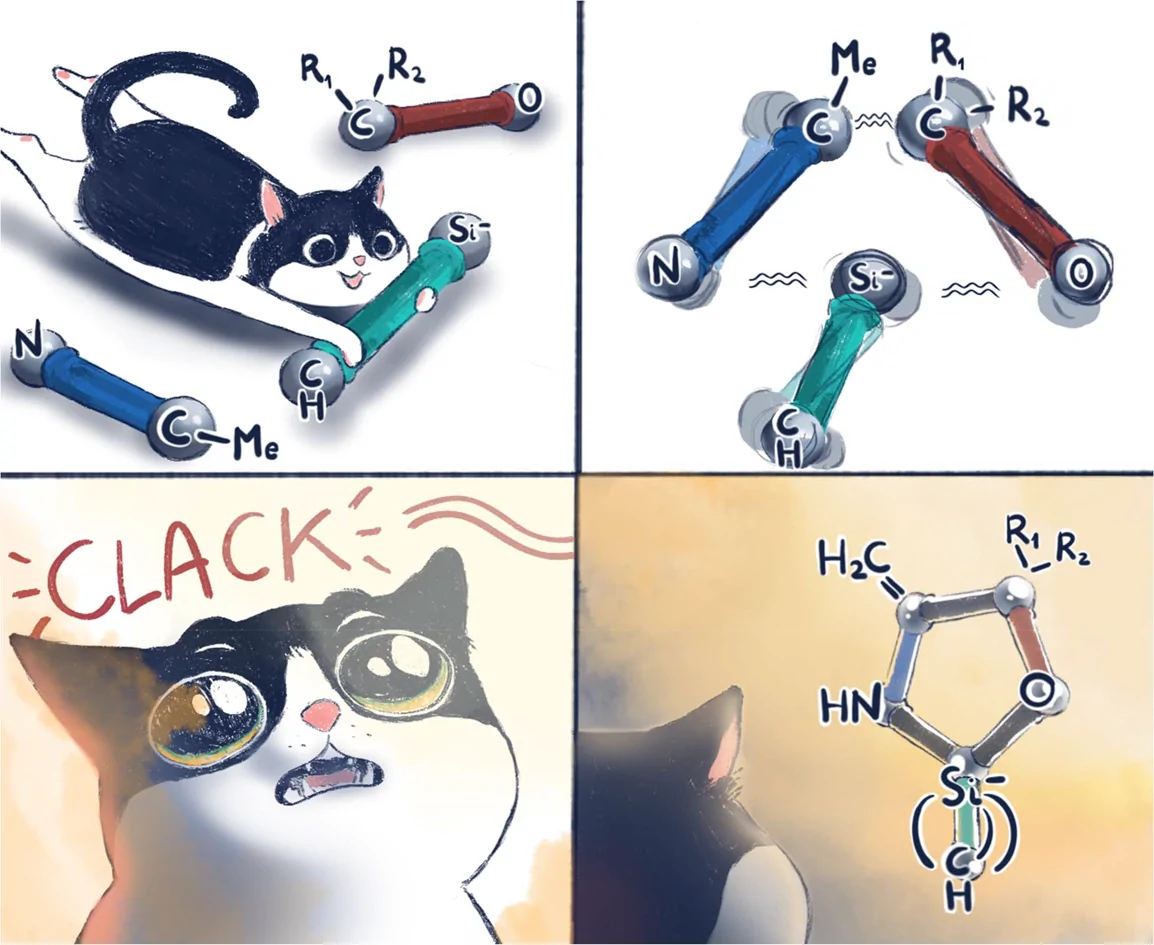

## Introduction

Carbon–carbon bond forming reactions occupy a central place in organic synthetic methodologies and in the pharmaceutical industry. In this context, so-called Umpolung reactions are an attractive way to form new C–C bonds between two fragments of similar polarity, most often two electrophiles. They proceed through the initial inversion of polarity of one of the coupling partners, allowing subsequent nucleophilic attack on another electrophilic center.^1–3^ Classical examples of reductive Umpolung reactions are benzoin condensation, the cyanide-catalyzed condensation of two aromatic aldehydes to form an α-hydroxyketone,^1,2^ and pinacol coupling, in which the reductive homocoupling of aldehydes or ketones afford a vicinal diol product.^4^ More recently, a range of related processes have been developed using N-heterocyclic carbene (NHC) catalysis *via* Breslow intermediates.^5^ Several redox-active metals have been applied to the development of selective (catalytic) reductive coupling reactions, including low valent early transition metals (V, Cr, Ti) and lanthanides (mostly Sm).^1–3,6–8^ Heavier main-group elements that can access a 2-electron formal redox couple are attractive reagents for Umpolung-type reactions. In this context, O'Reilly *et al.* showed that an Al(i) anion supported by bis-silylamido ligand NON ([NON]^2−^ = [O(SiMe_2_NDipp)_2_]^2−^) supports both homocoupling and cross-coupling pinacol-type reactions (Fig. 1a).^9^ A trapped ketyl derivative suggests that radical species are accessible under these conditions.^9^ The Ga(i) analogue K[Ga(NON)] also mediates the homocoupling of non-enolizable ketones (Fig. 1a).^10^ Nikonov *et al*. observed an unusual coupling of carbonyl substrates (non-enolizable ketones, amides and ketimines) with pyridine on a neutral tricoordinate Al(i) center to afford 2-coupled dihydro-pyridine complexes (Fig. 1b).^11^ An interesting example of a 2-electron Umpolung reaction using the P(iii/v) couple was recently reported by Radosevich *et al.* in a P(NMe_2_)_3_-mediated alkylation of α-keto esters (Fig. 1c).^12^

**Fig. 1:**
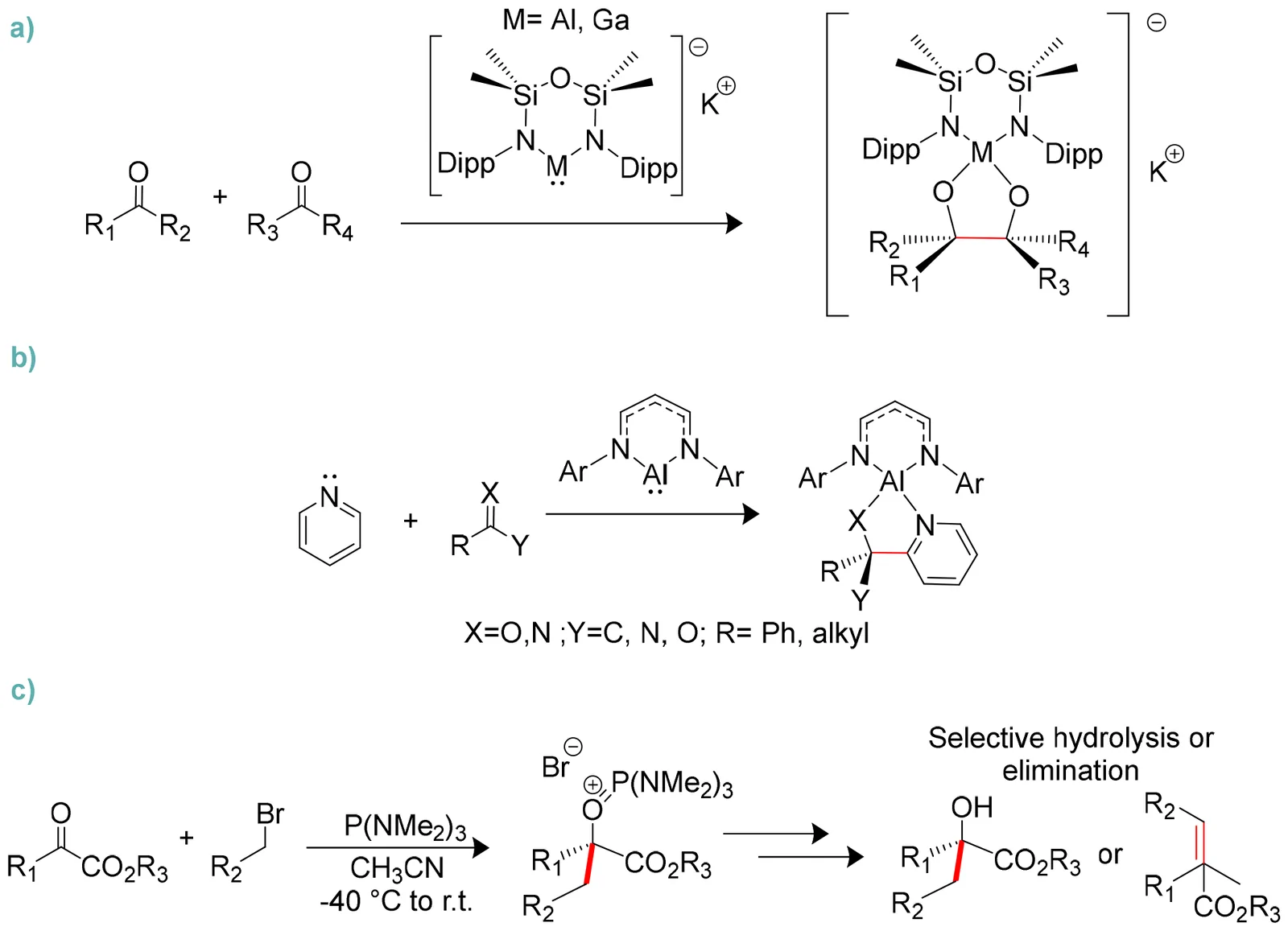
Examples of reductive coupling reactions at main group element centers.

Low valent silicon(ii) compounds, such as neutral (base-stabilized) silylenes, have been shown to support the reductive homocoupling of several organic fragments including alkynes, nitriles, aldehydes and ketones.^13–19^ Inoue *et al.* showed that an NHC-stabilized hydridosilylene reacts with diphenylacetylene to afford the corresponding silole^20^ (Fig. 2a). Wang *et al.* showed that an isolable dialkylsilylene can couple aromatic nitriles in either a head-to-head or head-to-tail fashion: electron-poor substrates give 1,3-diaza-2-siloles and electron-rich ones give 1,4-diaza-2-siloles exclusively (Fig. 2b).^21^

**Fig. 2:**
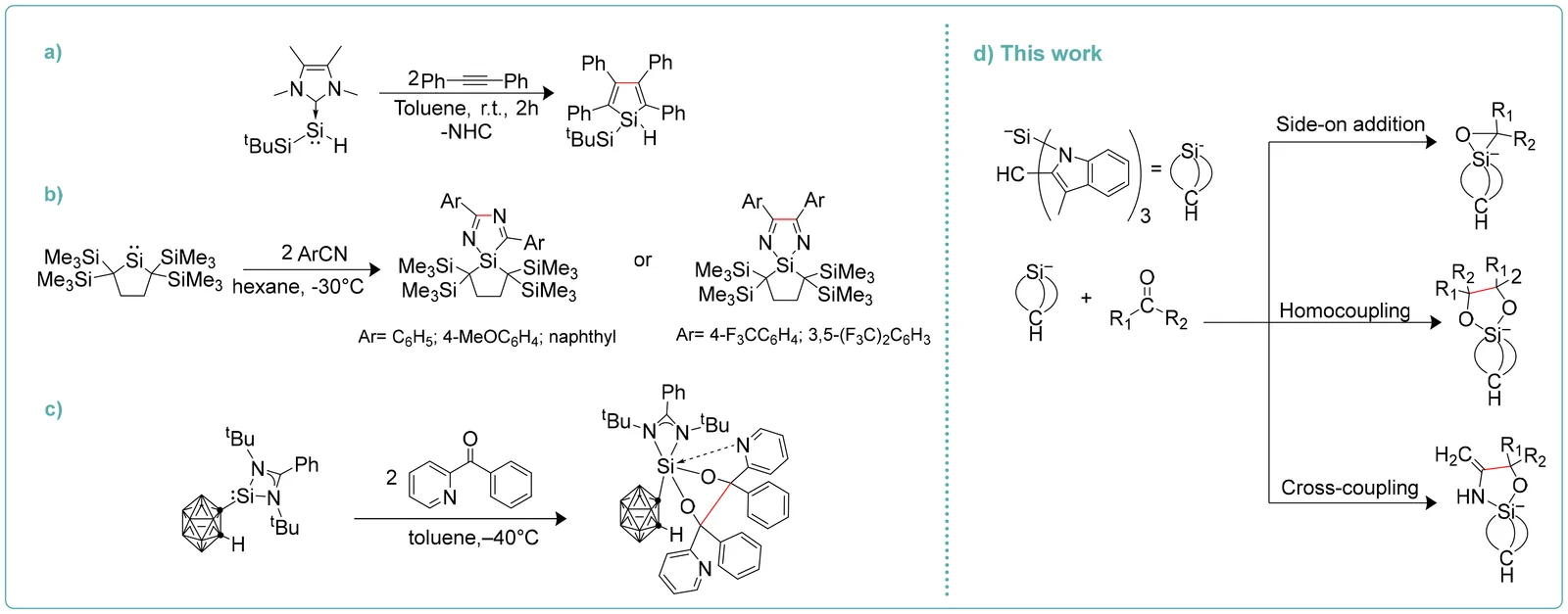
Prior examples of reductive homocoupling reactions at silicon and the cross-coupling reaction reported here.

Silylenes have been shown to react with ketones in various ways. For example, Li *et al.* showed that an NHC-stabilized Si(ii) silylene forms silicon bis-enolates with enolizable ketones while benzophenone undergoes bis-silylation of the C=O group.^22^ Similarly, Driess *et al.* investigated reactions of an isolable zwitterionic silylene with benzophenone, acetophenone and benzylideneacetone which led to addition/cycloaddition and rearrangements.^23^ Some examples of carbonyl homocoupling have been reported using silicon compounds including silole dianions,^24^ a chlorotrisyl-silylenoid as silylene precursor,^25^ and a boryl–ethynyl silylene with 9-fluorenone.^26^ In a key example, Xie *et al.* showed that a carborane-fused silylene can couple 2 eq. of 2-benzoylpyridine (Fig. 2c); based on the isolation of an adduct with benzophenone, the authors proposed a stepwise [2 + 1] cycloaddition/insertion mechanism for this reaction.^16^

Silanides ([R_3_Si^−^]) are best known for their nucleophilic reactivity illustrated in (conjugate) addition to (α,β-insaturated) carbonyl compounds.^27,28^ When a silyl-substituted silanide is used, silyl-group migration can initiate the formation of (transient) silene derivatives *via* a sila-Peterson mechanism.^29,30^ Recently, certain silanides have been shown to display biphilic reactivity akin to the neutral silylenes analogues. For example, perfluorinated silanides have been shown to undergo oxidative addition of C–F bonds^31^ and side-on additions of benzaldehyde and benzophenone by the group of Hoge.^32^ Liu *et al.* reported the isolation and full characterizations of the first T-shaped planar silanide, where the silicon centre features a lone pair of electrons and a vacant 3p orbital. This compound can undergo oxidative additions to various chemical bonds under mild conditions.^33^ We recently reported the oxidative addition reactivity of a cage silanide 1 [K(18-crown-6)][(tmim)Si] derived from tris(3-methylindol-2-yl)methane (tmimH_3_) with polar element-hydrogen bonds (X–H, X = O, S, N)^34^ and the observation of the reverse O–H reductive elimination process upon collision induced dissociation (CID) in a mass spectrometer.^35^

Here, we report on the reactivity of cage silanide 1 with carbonyl compounds and nitriles. We observe the unusual formation of 5-membered silacycles resulting from a formal reductive cross-coupling of a ketone and a nitrile. The ketone and the nitrile coupling partners can each also form homocoupling products, but slower. DFT calculations support an Umpolung mechanism involving initial condensation of the ketone and silanide to form an oxasiliranide – an analogue of which can be isolated for benzaldehyde – that readily opens at the Si–C bond to insert a nitrile molecule.

## Results and discussion

### Synthesis and characterization

Treatment of silanide 1 ^34,36^ with an equimolar amount of benzaldehyde in MeCN at r.t. for 2 h led to a side-on addition of the carbonyl group of the benzaldehyde to afford a potassium salt of the corresponding oxasiliranide anion 2 (Scheme 1). Compound 2 slowly decomposes at r.t. forming an intractable mixture of products. The same compound is formed if other solvents such as dioxane or THF are used. The isolated compound 2 product was characterized by multinuclear NMR spectroscopy and X-ray crystal structure determination. Next to the signals of the tmim ligand and the aromatic ring derived from benzaldehyde, the ^1^H-NMR spectrum of 2 displays a diagnostic singlet at 5.33 ppm that is directly bonded to a carbon atom with a ^13^C-NMR chemical shift of 70.8 ppm and corresponds to the oxasiliranide proton. The considerable downfield shift from 192.3 in free benzaldehyde^37^ is in line with the ^13^C-NMR shift of 75.5 ppm reported by Hoge^32^ for the analogous oxasiliranide anion derived from (CF_3_CF_2_)_3_Si^−^. In the crystal structure of 2 (Fig. 3), the silicon-bound benzaldehyde was refined with a disorder model, and all hydrogen atoms were introduced in calculated positions. Based on the angles, around the Si center, the geometry can be described as a strongly distorted square pyramid with one of the chelating nitrogens at the apex. Next to Hoge's anion,^32^ the three-membered Si–O–C heterocycle has a few neutral precedents obtained by side-on addition of silylenes to carbonyl compounds.^16,18,38–44^

**Scheme 1:**
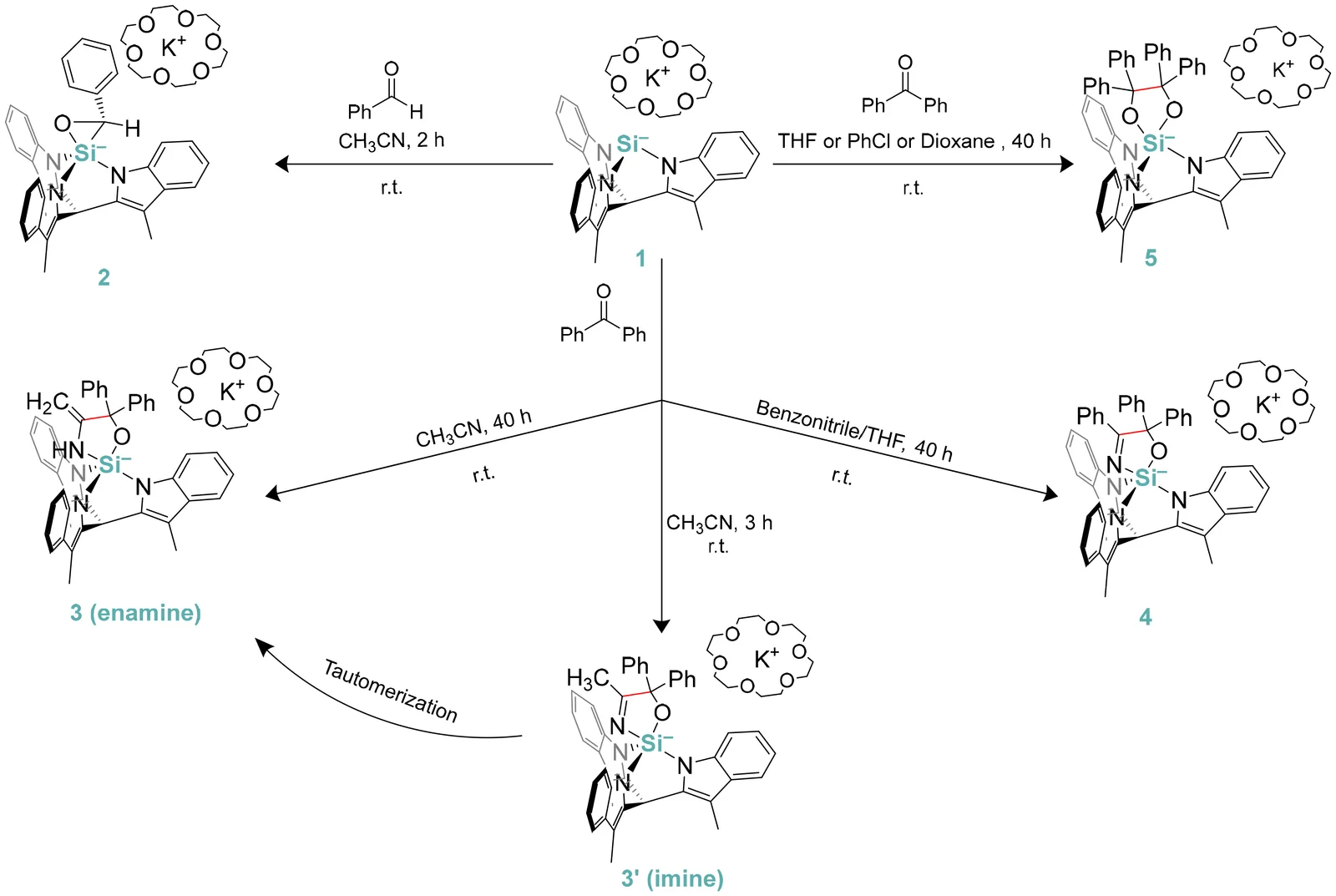
Reaction of compound 1 with benzaldehyde and benzophenone.

**Fig. 3:**
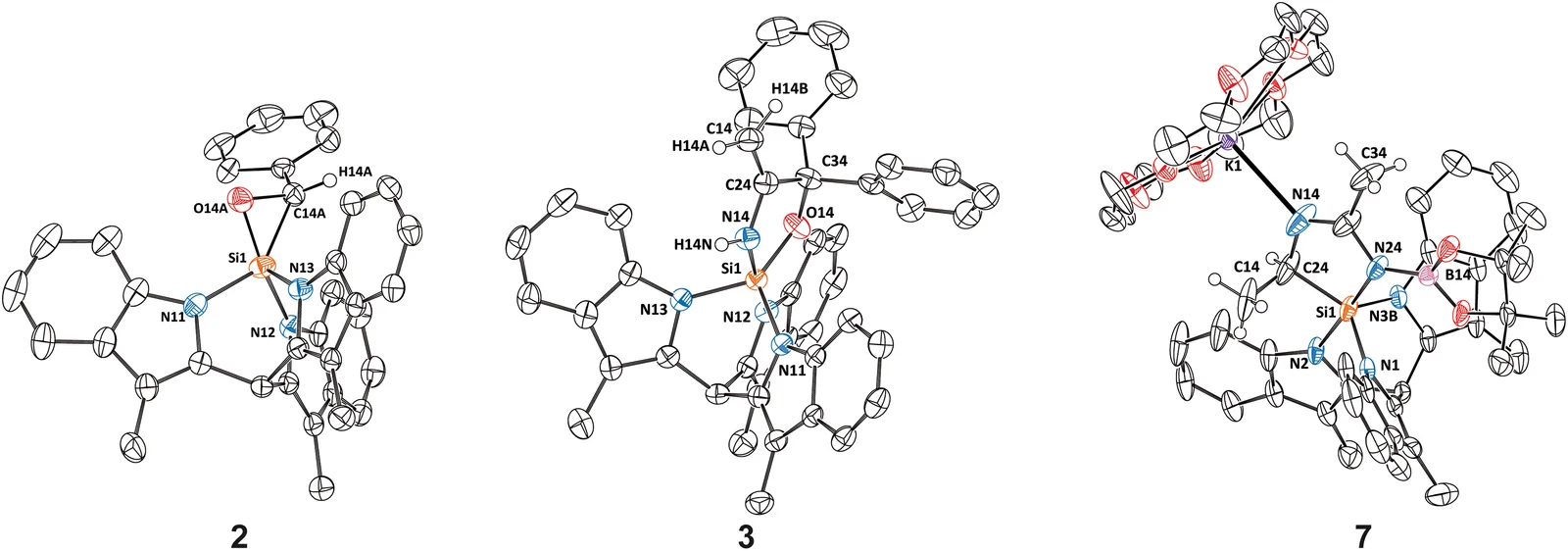
Molecular structures of compound 2, 3, and 7 in the crystal. Displacement ellipsoids are drawn at the 50% probability level. Countercations, solvents and most hydrogen atoms were omitted for clarity. Only the major components of disordered moieties are shown.

In contrast with benzaldehyde, the treatment of free silanide 1 with an equimolar amount of benzophenone at room temperature did not lead to an observable oxasiliranide anion. Instead, we observed the formation of different five-membered cycles depending on the solvent in which the reaction was performed (Scheme 1). A reaction performed in MeCN for 40 h yielded cyclic enamine 3, which could be isolated in 80% yield. The crystal structure of 3 shows a 5-membered cycle obtained by the formation of a new C–C single bond (153.0 (4) pm) between the electrophilic carbon atoms of MeCN and benzophenone. Moreover, the C–N (137.0(3) pm) and C–C (133.5 (4) pm) distances differ considerably from those in free MeCN (115.1(10) and 143.6(10) pm^45^) and are consistent with a single C–N bond and a double C=C bond in the corresponding fragment. The 5-membered cycle adopts an envelope conformation with the O-bound carbon atom in the *endo* (out-of-plane) position. The geometry of the Si center in 3 can be described as a slightly distorted trigonal bipyramid (TBP) with 2 N atoms from the tmim fragment and the O atom from the cycle in the equatorial positions. The remaining N atom from the tmim fragment and the N atom from the cycle are in the axial position, with an almost linear N12–Si–N14 angle (170.31(10)°) similar to those in previously reported pentacoordinated hydro-silicates [K(18-crown-6)][(tmim)SiH(XC_6_H_4_R)] (X = O, S, NH, R = H or CH_3_) derived from silanide 1.

Solution 1D and 2D NMR spectra of 3 are consistent with the solid-state structure. The –NH (4.02 ppm) and the =CH_2_ (3.48 ppm) signals of the enamine moiety were identified using HSQC measurement in MeCN-d_3_. Both of these signals are absent when the reaction is performed in CD_3_CN, confirming their chemical origin (see SI section 2.1). One of the aromatics ^1^H-NMR signals of the tmim moiety at 7.60 ppm is very broad; this suggested the presence of fluxional process that was studied using VT NMR. Cooling a solution of 3 in DCM-d_2_ to −85 °C results in the sharpening, shifting and splitting of the broad ^1^H signal of the tmim moiety (7.60 ppm, 3H) into two sharp doublets (8.29 ppm, 1H and 7.03 ppm, 2H) while the other signals experience only slight shifts (see SI section 2.4). We attribute this behavior to the C–*H* nucleus in position 7 of one of the indole rings being magnetically shielded by the neighboring aromatic rings from the diphenylmethylene moiety and exchanging with the other indole rings at higher temperatures *via* a Berry pseudorotation process around the pentacoordinate Si center.

The formation of 3 was hypothesized to proceed *via* tautomerization of an imine intermediate 3′ that would be more directly derived from MeCN. This was confirmed by working up the reaction after 3 h only, which allowed isolation of the imine tautomer 3′ in 87% yield. Compound 3′ displays a characteristic CH_3_^1^H NMR signal at 2.44 ppm that is absent from the spectrum when the reaction is performed in CD_3_CN. As anticipated, the isolated compound 3′ slowly tautomerizes to 3 in MeCN at RT, converting completely in *ca.* 40 h. The tautomerization can be accelerated by heat. In DCM, solutions of either 3 or 3′ reach an equilibrium 3 : 3′ ratio of about 4 : 1 within minutes (see SI). Performing the reaction using 2.2.2-Cryptand^28^ instead of the 18-crown-6 as complexant for K^+^ did not hinder the formation of 3, suggesting that the reaction is not assisted by the potassium cation (see SI). A stable analogue of 3′ was obtained by using benzonitrile instead of MeCN to prevent tautomerization. The reaction afforded cyclic imine 4, which was thoroughly characterized by 1D-NMR, 2D-NMR, IR analysis and ESI-MS.

First, the anionic mode ESI-MS spectrum of compound 4 displays a signal at *m*/*z* = 732 (M + H_2_O) indicating that one benzonitrile and one benzophenone molecule have been condensed with silanide 1. The IR spectrum of 4 displays a peak at 1609 cm^−1^, which can be associated with the C=N bond.^46^ The ^13^C-NMR signal for the C=N was identified at 176.3 ppm and the C–O signal was found at 86.9 ppm, confirming a C=N double bond and a C–O single bond. The ^1^H-NMR signals at 2.46 ppm (6H) and 2.43 ppm (3H), corresponding to the methyl groups of the tmim ligand, suggest an overall *C*_s_ symmetry with a mirror plane containing the 5-membered cycle and one of the indole units of the tmim ligand. Evidently, the imine-bound phenyl group in 4 hinders the pseudorotation process observed in 3 at least up to room temperature.

The selective formation of 3′ and 4 from benzophenone and acetonitrile constitutes an unusual cross-electrophile reductive coupling. This prompted us to investigate the possibility of related homocoupling reactions. When 1 is treated with benzophenone in THF, PhCl or dioxane, thus in absence of MeCN, a different heterocycle is formed. The anionic mode ESI-MS spectrum of compound 5 displays a signal at *m*/*z* = 793 indicating that two benzophenone molecules have been added to 1. Similarly to 4, a single ^13^C NMR signal of the C–O unit derived from benzophenone is found at 89.3 ppm in 5, indicating a symmetrical structure with an sp^3^-hybridized carbon atom. A ^29^Si NMR signal at −118.4 ppm supports a 5-coordinated Si center. These data, together with detailed 2D NMR data (see SI), support the formulation of 5 as a 5-membered 2-sila-1,3-dioxolane formed by reductive homocoupling of two benzophenone molecules (Scheme 1). If the reaction is performed using 1 eq. of benzophenone and 1 eq. of MeCN, a mixture of products including 3, 3′ and 5 are obtained (see SI) showing a competition between the two reactions.

A different kind of homocoupling reaction was observed for acetonitrile. While compound 1 can be handled in acetonitrile solution for short times, leaving such a solution standing for 2 days led to the formation of cyclic 2,4-diazasilole 6, in which two MeCN molecule couple *head to tail* to form a 5-membered cycle with the formation of a new C–N bond (Scheme 2). The ^1^H-NMR spectrum of 6 displays three CH_3_ signals: one at 2.46 ppm (9H) corresponding to the tmim ligand and two signals at 2.54 ppm (3H) and 2.38 ppm (3H) corresponding to the methyl groups originating from the MeCN reactant. The strongly deshielded ^13^C-NMR signals for the two C=N carbon nuclei were identified at 182.2 ppm and 178.7 ppm.

**Scheme 2:**
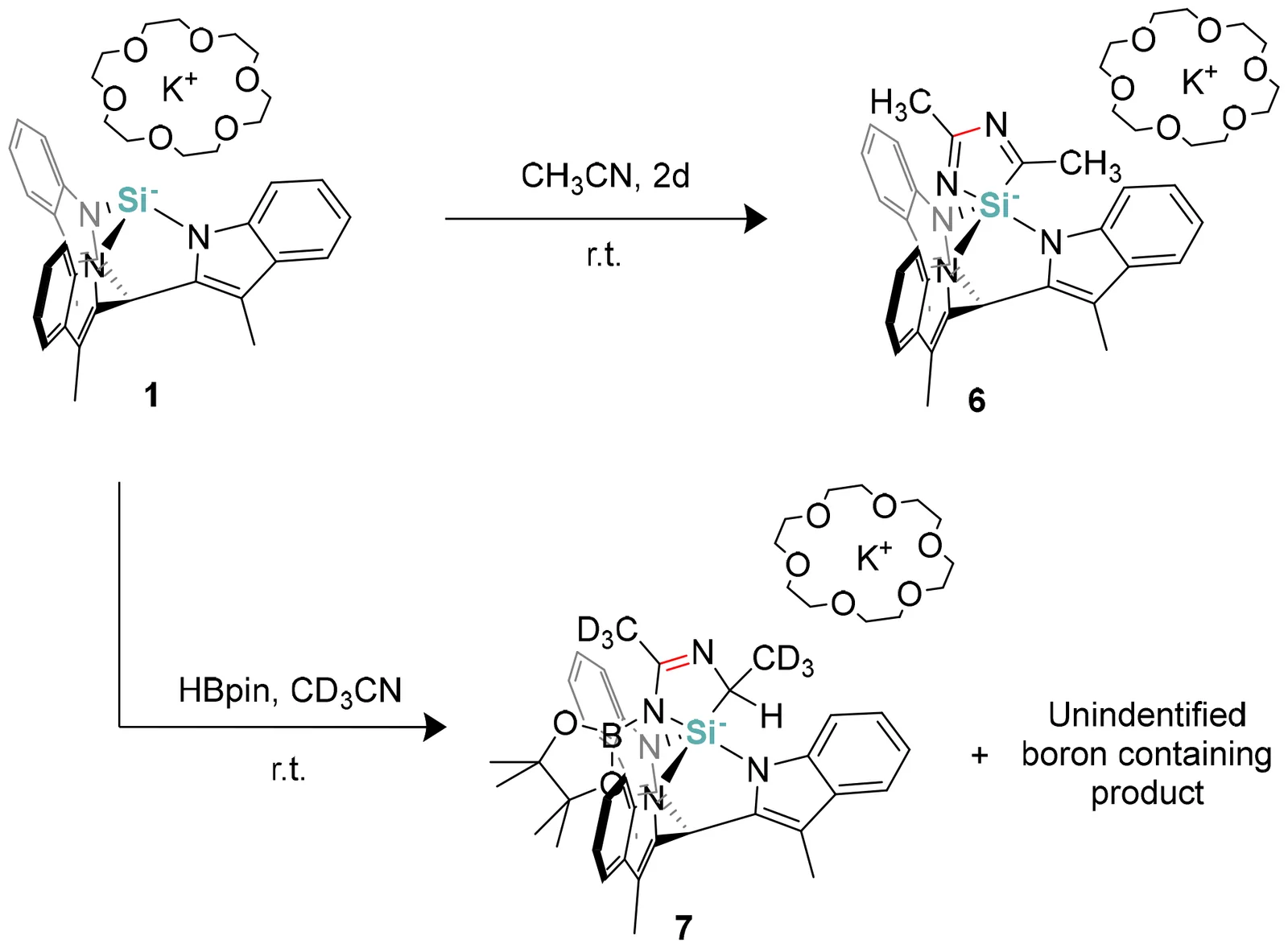
Reaction of compound 1 with MeCN and MeCN-d_3_ and HBpin.

While no crystals of 6 suitable for X-ray diffraction could be obtained, small amounts of a crystalline derivative 7 were obtained from the reaction of compound 1 with HBpin in MeCN-d_3_ (see SI). Monitoring the *in situ* reaction revealed the formation of a mixture of products of which one (7) deposited X-ray quality crystals over time. The crystal structure of 7 showed a 5-membered heterocycle that would be the result of a formal hydroboration of 6 and confirms the head to tail condensation of two acetonitrile molecules (Scheme 2 and Fig. 3).

### Computational study

To investigate the mechanistic details of the formation of silacycles 2–5, DFT calculations were performed using the anionic moiety without the counteraction. Geometries were optimized at the B3LYP-GD3BJ/6-311++g(d,p) level of theory; the effect of the solvent was included in single point calculations using SMD (Solvation Model based on Density) for THF and MeCN. Both solvents afforded similar Gibbs energy values (see SI section 4) and the energies using MeCN are showed in Scheme 3. Calculations on the formation of 6 were also performed, but both concerted and stepwise pathways were found to be energetically prohibited (see SI section 4.2) suggesting that a more complex mechanism (*e.g.* radical) may be at play. Formation of silaepoxide A from 1 and benzophenone is slightly endergonic at 1.2 kcal mol^−1^. Intermediate A can equilibrate with its open form (B) located at 7.4 kcal mol^−1^*via* a readily accessible transition state at 13.1 kcal mol^−1^. NBO analysis of these intermediates show that both are best described as Si(iv) compounds bound to a doubly reduced benzophenone fragment (see SI section 4.4).

**Scheme 3:**
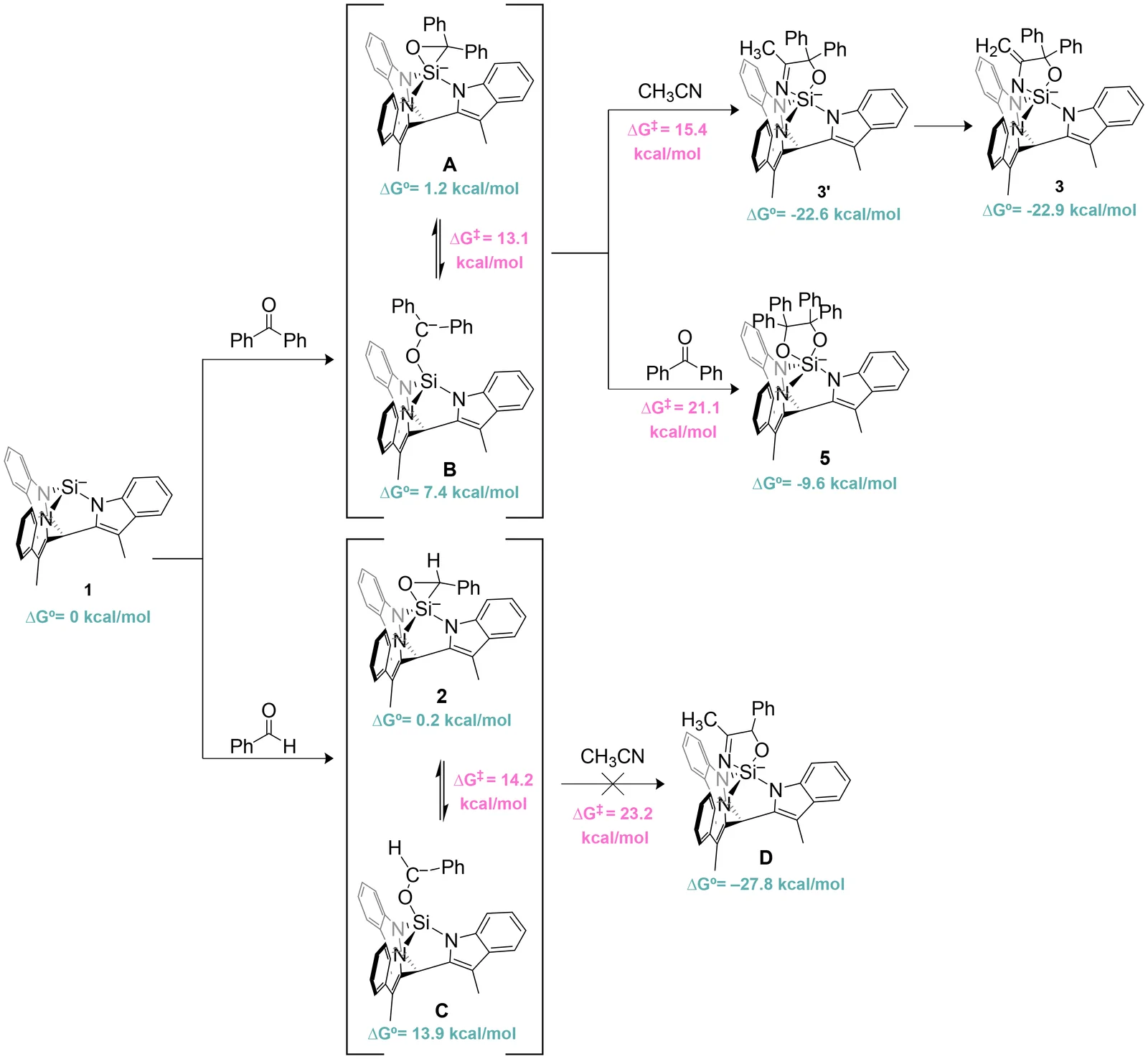
Gibbs free energies for the intermediates (blue) and transition states (magenta) relevant to the formation of silacyles 2–5, calculated at the B3LYP-GD3BJ//6-311++g(d,p)/SMD(CH_3_CN)//B3LYP-GD3BJ//6-311++g(d,p) level of theory.

In compound A, the silaepoxide features an alkoxide and a Si–C bond, whose bonding orbital is strongly polarized towards carbon (76%). In compound B, the principal Lewis structure describes the oxygen as an alkoxide with three lone pairs, with the remaining negative charge delocalized in the phenyl rings. These findings support the idea that the interaction between the silanide and benzophenone induces an Umpolung of the carbon atom and renders it nucleophilic. From compound B, addition of CH_3_CN leads to the formation of the imine cycle 3′ with an accessible barrier of 15.4 kcal mol^−1^. Imine cycle 3′ is located at Δ*G*° = −22.6 kcal mol^−1^ and subsequently by tautomerization forms enamine 3, which is located at Δ*G*° = −22.9 kcal mol^−1^. In the absence of acetonitrile, an additional benzophenone molecule can react with B to form the silacycle 5 with a higher barrier of 21.1 kcal mol^−1^. In both transition states, the silacycle is formed in a single step; stepwise routes were explored but presented at higher energy barrier (see SI).

## Conclusions

In summary, we report the reactivity of the cage silanide 1 toward carbonyl compounds and nitriles, leading to the formation of new C–C bonds and affording both homo- and cross-coupling products. The resulting five-membered silacycles were isolated and fully characterized. DFT calculations support a proposed Umpolung mechanism involving the initial condensation of the ketone with the silanide to generate an anionic siloxirane intermediate, which subsequently undergoes facile Si–C bond cleavage and nitrile insertion. These findings demonstrate that silanides can serve as effective platforms for the coupling of ketones and nitriles. Current efforts are directed toward the release of the coupled products under reductive conditions to regenerate the silanide species, a key requirement for the future development of catalytic processes using silanides.

## Author contributions

Dr M.-E. Moret initiated and supervised the project. P. A. Benzan Lantigua performed the experiments and Dr M. L. G. Sansores-Paredes performed the DFT calculations. Dr M. Lutz oversaw the acquisition, solving and interpretation of the X-ray crystal structure. P. A. Benzan Lantigua and Dr M. L. G. Sansores-Paredes wrote the manuscript with input from Dr M.-E. Moret and with the contribution of Dr M. Lutz. All authors approved the final manuscript.

## Conflicts of interest

There are no conflicts to declare.

## Supplementary Material

QI-OLF-D6QI01961G-s001

QI-OLF-D6QI01961G-s002

## Data Availability

All experimental procedures, analytical data and computational data supporting the findings of this study are available within the article and its supplementary information (SI). Supplementary information is available. See DOI: https://doi.org/10.1039/d6qi01961g. CCDC 2564771–2564773 (2, 3 and 7) contain the supplementary crystallographic data for this paper.^47*a*–*c*^
