# Supplementary material for: Reductive coupling of ketones and nitriles at a geometrically constrained silanide
Source: Inorg Chem Front. 2026 Oct 29. Online ahead of print. doi: 10.1039/d6qi01961g (PMC13732756; doi:10.1039/d6qi01961g)
Supplement: Supplementary file 1 [file QI-OLF-D6QI01961G-s001.pdf]

This journal is © the Partner Organisations

Reductive Coupling of Ketones and Nitriles at a Geometrically Constrained Silanide

Pamela Adienes Benzan Lantigua, María L. G. Sansores-Paredes; Martin Lutz and Marc-Etienne Moret*

Abstract: While silanides ($[R_3Si^-]$) are best known for their nucleophilic reactivity, examples have recently been shown to display biphilic reactivity akin to neutral Si(II) compounds. In this context, we report on the reactivity of silanide [K(18-crown-6)][tmimSi] ($tmimH_3$ = tris(3-methylindol-2-yl)methane) with carbonyl compounds and nitriles. The unusual reductive cross-coupling of a ketone and a nitrile result in the formation of 5-membered silacycles. The ketone and the nitrile coupling partners are each able to form homocoupling products, but at a slower rate. DFT calculations support an Umpolung mechanism involving initial condensation of the ketone and silanide to form an anionic oxasilirane – an analogue of which can be isolated for benzaldehyde – that readily opens at the Si–C bond to insert a nitrile molecule.

Table of Contents

1. Experimental Procedures 3
    - 1.1 General information 3
    - 1.2. Synthesis and characterization 3
2. Additional experiments 6
    - 2.1. Imine-Enamine Tautomerization 6
    - 2.2. Synthesis of the imine cycle 3' using $[K(2.2.2\text{-cryptand})]^+[tmimSi]^-$ 8
    - 2.3. Synthesis of the enamine cycle using 1 eq of MeCN 10
    - 2.4. VT-NMR of the enamine product 3 in DCM-d2 10
    - 2.5. Reaction of compound 1 with HBpin in CD_3CN 12
    - 2.6. IR analysis 13
3. X-Ray Crystal structure determinations 15
    - 3.1. X-ray crystal structure determination of Compound 2 15
    - 3.2. X-ray crystal structure determination of Compound 3 15
    - 3.3. X-ray crystal structure determination of Compound 7 16
4. DFT studies 17
    - 4.1. Computational methods. 17
    - 4.2. Comparison of the Gibbs energy using SMD= MeCN or THF. 17
    - 4.3. Calculations on the formation of MeCN silacycle (6) 18
    - 4.4. Additional calculations on the formation of the silacycles derived from benzophenone. 21
    - 4.5. Table of energies 23
    - 4.6. Coordinates of calculated structures **Error! Bookmark not defined.**
5. Spectra of isolated compounds 24
6. References 40
7. Author Contributions 40

1. Experimental Procedures

1.1. General Information

All reactions involving air-sensitive compounds were conducted under an N2 atmosphere by using standard glovebox or Schlenk techniques. Acetonitrile, n-hexane and Et_2O were dried with an MBRAUN MB SPS-79 system. THF was distilled from benzophenone/Na, and DCM was dried over 4Å molecular sieves. All solvents were degassed by bubbling with N_2 and stored over molecular sieves in a glovebox. Deuterated acetonitrile, THF, and DCM were degassed by three freeze-pump-thaw cycles and stored over molecular sieves in a glovebox. Skatole, KH 30 w% in mineral oil, 2,6-diisopropylaniline, $HSiCl_3$, KOtBu (≥97%), benzaldehyde and benzophenone were purchased from Sigma-Aldrich. Triethyl orthoformate, glyoxal 40 w%, TMSCl, paraformaldehyde, 18-crown-6 were purchased from Acros. All chemicals used in this work except the synthetized ones were commercial products of the highest available purity and were further purified by the standard methods, if necessary. Cage silanide **1** and its precursors were prepared according to the reported procedure.[1–3] All NMR measurements were performed on a Varian VNMRS400 or Varian MRF400 spectrometer. The chemical shifts are reported relative to TMS with the residual solvent signal as internal standard. All NMR experiments involving air-sensitive compounds were conducted in J-Young NMR tubes under an N_2 atmosphere. ESI-MS measurements were performed on a Waters LCT Premier XE KE317 spectrometer. Infrared spectra were recorded using a Perkin Elmer Spectrum One FT-IR spectrometer under N_2 flow when necessary. Due to the high sensitivity of the compounds, no satisfactory combustion analysis data could be obtained for compounds **2-6**. In support of the bulk purity of isolated compounds, NMR spectra for new compounds are reproduced in full in the current document.

1.2. Synthesis and Characterization

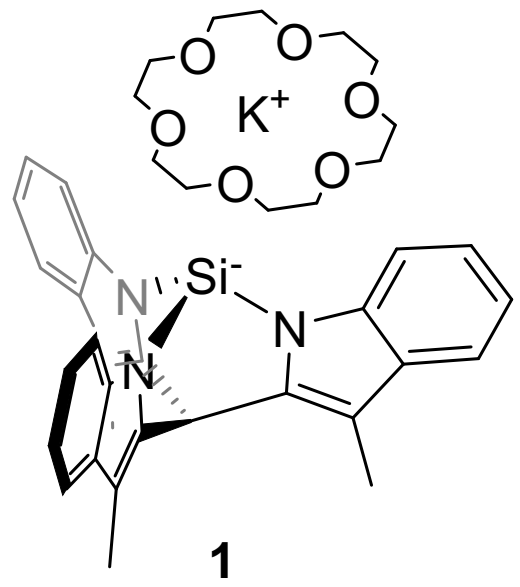

1

Synthesis of [K(18-crown-6)]$^+$[(tmim)Si]$^-$ (1). The synthesis was performed as previously reported.[1,2] (ref)

^{1}H NMR (400 MHz, MeCN-d_3) δ 7.73 (dt, *J* = 8.1, 1.0 Hz, 3H, indole-*H*7), 7.32 (dt, *J* = 7.8, 1.0 Hz, 3H, indole-*H*4), 6.97 (ddd, *J* = 8.2, 7.0, 1.3 Hz, 3H, indole-*H*5), 6.87 (ddd, *J* = 7.9, 7.0, 1.1 Hz, 3H, indole-*H*6), 5.95 (s, 1H, R3C*H*), 3.56 (s, 36H, [18-crown-6]), 2.42 (s, 9H, CH_3).

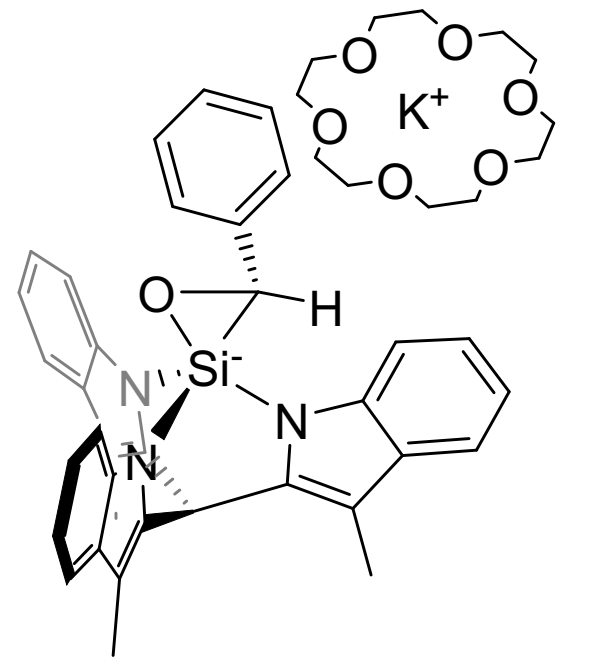

2

Synthesis of oxasiliranide [K(18-crown-6)]$^+$[(tmim)Si(CH(Ph)O)]$^-$ (2). Benzaldehyde (4µL, 0.0394 mmol) was added to a suspension of [K(18-crown-6)]$^+$[(tmim)Si]$^-$ (30.1 mg, 0.0411 mmol) in MeCN (1.5 mL). The mixture was stirred for 1h, over which time the cloudy white suspension turned to a clear light-yellow solution. The resulting solution was filtered, the solvent was evaporated *in vacuo*, and the resulting off-white powder was dissolved in the minimal amount of MeCN and precipitated using Et_2O. The supernatant was removed, and the precipitate washed with Et_2O. Drying *in vacuo* afforded the product as an off-white powder 13.3 mg 0.0159 mmol, 40%). Crystals suitable for X-ray crystallography were grown by vapor diffusion of Et_2O into a concentrated solution of **2** in MeCN at –35°C.

^{1}H-NMR (400 MHz, CD_3CN, 25°C) δ 7.72 (d, *J* = 8.0 Hz, 2H, CPhHO-H_{Ar}), 7.54 (d, *J* = 8.1 Hz, 3H, Indole-*H*), 7.39 (t, *J* = 7.9 Hz, 2H, CPhHO-H_{Ar}), 7.31 (d, *J* = 8.1 Hz, 3H, Indole-*H*), 7.20 (t, *J* = 7.4 Hz, 1H, CPhHO-H_{Ar}), 6.83 (m, *J* = 20.0, 7.1 Hz, 6H, Indole-*H*), 5.92 (s, 1H, Indole-C*H* linker), 5.32 (s, 1H, CPh*H*O), 3.52 (s, 24H,18-crown-6), 2.41 (s, 9H, Indole-CH_3). ^{1}H-NMR (400 MHz, CD_2Cl_2, 25°C) δ 7.74 (d, *J* = 8.0 Hz, 2H, CPhHO-H_{Ar}), 7.61 (d, *J* = 8.9 Hz, 3H, Indole-*H*), 7.39 (t, *J* = 7.7 Hz, 2H, CPhHO-H_{Ar}), 7.30 (d, *J* = 7.6 Hz, 3H, Indole-*H*), 7.19 (t, *J* = 7.4 Hz, 1H, CPhHO-H_{Ar}), 6.84 (m, *J* = 7.0, 1.5 Hz, 6H, Indole-*H*), 5.86 (s, 1H, Indole-C*H* linker), 5.39 (s, 1H, CPh*H*O), 3.19 (s, 24H, 18-crown-6), 2.41 (s, 9H, Indole-CH_3). **^{13}C{^{1}H}-NMR** (APT, 101 MHz, CD_3CN, 25°C) δ 149.8 (s, CPhHO-C_{Ar}), 142.6 (s, C_{Indole}), 140.7(s, C_{Indole}), 131.2 (s, C_{Indole}), 129.4 (s, CPhHO-CH_{Ar}), 128.6(s, CPhHO-CH_{Ar}), 125.5 (s, CPhHO-CH_{Ar}), 121.1 (s, CH_{Indole}), 118.7 (s, CH_{Indole}), 118.5 (s, CH_{Indole}), 114.7 (s, CH_{Indole}), 105.2 (s, C_{Indole}), 72.5 (s, *C*PhHO), 70.8 (s, CH_2,18-crown-6), 32.7 (s, CH_{Indole} linker), 8.6 (s, $CH_{3\text{-}Indole}$).

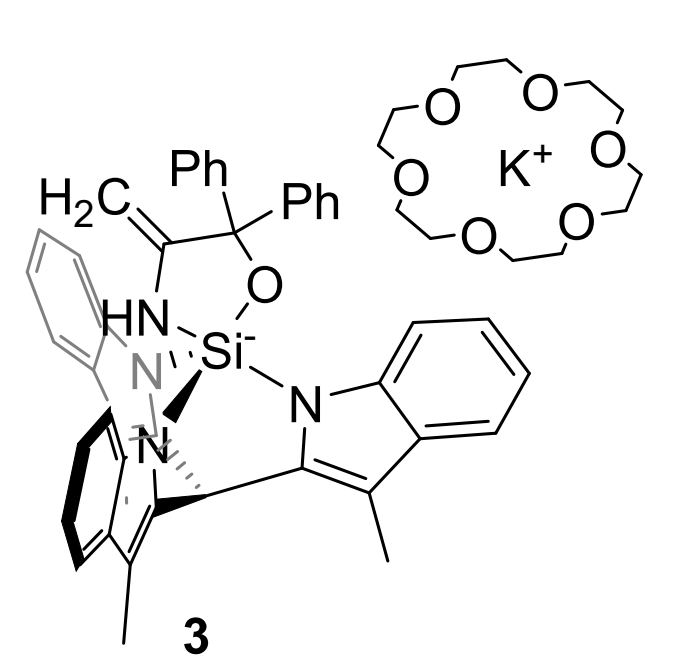

3

Synthesis of enamine cycle 3. Benzophenone (7.4 mg, 0.0406 mmol) was added to a suspension of $[K(18\text{-crown-}6)]^+[(tmim)Si]^-$ (30.3 mg, 0.0414mmol) in MeCN (2.5 ml). The mixture was stirred for 40h, over which time the cloudy white suspension turned to a clear light-yellow solution. The resulting solution was filtered, the solvent was removed *in vacuo,* and the resulting off white powder was dissolved in the minimum amount of MeCN and precipitated using n-pentane. The supernatant was removed, and the precipitate washed with Et_2O. Drying *in vacuo* afforded the product as a light yellow (30.9 mg, 0.0384 mmol, 80%).

^{1}H NMR (400 MHz, CD_3CN, 25°C) δ 7.93 (dt, *J* = 6.3, 1.5 Hz, 4H, Ph_2CO-H_{Ar}), 7.49 (br, 3H, Indole-*H*), 7.41 – 7.29 (m, 6H, Ph_2CO-H_{Ar}), 7.26 (d, *J* = 7.8 Hz, 3H, Indole-*H*), 6.79 (t, *J* = 7.3 Hz, 3H, Indole-*H*), 6.63 (ddd, *J* = 8.3, 6.9, 1.3 Hz, 3H, Indole-*H*), 5.86 (s, 1H, Indole-C*H* linker), 4.10 (d, *J* = 5.5 Hz, 1H, NHCCH_2), 4.02 (d, *J* = 1.7 Hz, 1H, N*H*CCH_2), 3.50 (s, 24H, 18-Crown-6), 3.48 (d, J = 1.8 Hz, 1H, NHCCH_2), 2.40 (s, 9H, Indole-CH_3). ^{1}H NMR (400 MHz, CD_2Cl_2, 25°C) δ 7.98 – 7.92 (m, 4H, Ph_2CO-H_{Ar}), 7.59 (s, 3H, Indole-*H*), 7.37 – 7.24 (m, 12H, Indole-*H* and Ph_2CO-H_{Ar}), 6.84 – 6.80 (m, 3H, Indole-*H*), 6.71 (ddd, *J* = 8.3, 6.9, 1.4 Hz, 3H, Indole-*H*), 5.83 (s, 1H C*H* linker), 4.06 (s, 1H, N*H*CCH_2), 3.99 (s, 1H, NHCCH_2), 3.57 (s, 1H, NHCCH_2), 3.12 (s, 24H,18-Crown-6), 2.40 (s, 9H, Indole-CH_3). **^{13}C{^{1}H}-NMR** (APT, 101 MHz, CD_3CN, 25°C) δ 159.2 (s, PhCN), 148.1 (s, NH*C*=CH_2), 142.8 (s, C_{Indole}), 141.0 (s, C_{Indole}), 131.2 (s, C_{Indole}), 128.9 (s, Ph_2CO-CH_{Ar}), 128.6(s, Ph_2CO-CH_{Ar}), 127.9(s, NHCCH_2), 120.1(s, CH_{Indole}), 117.5(s, CH_{Indole}), 115.7(s, CH_{Indole}), 103.8(s, C_{Indole}), 84.5 (s, Ph_2CO), 76.6(s, NHC=CH_2), 71.5 (s, CH_2,18-crown-6), 32.4 (s, CH_{Indole} linker), 8.6(s, $CH_{3\text{-}Indole}$). (CH_{Indole} around 118.3 ppm covered by solvent signal). **^{29}Si NMR** (79 MHz, CD_2Cl_2) δ -119.11 (this NMR spectrum was recorded on the partially deuterated compound derived from CD_3CN). **ESI-MS$^-$** ($C_{43}H_{36}N_4OSi^-$) m/z calculated 652; found 652.

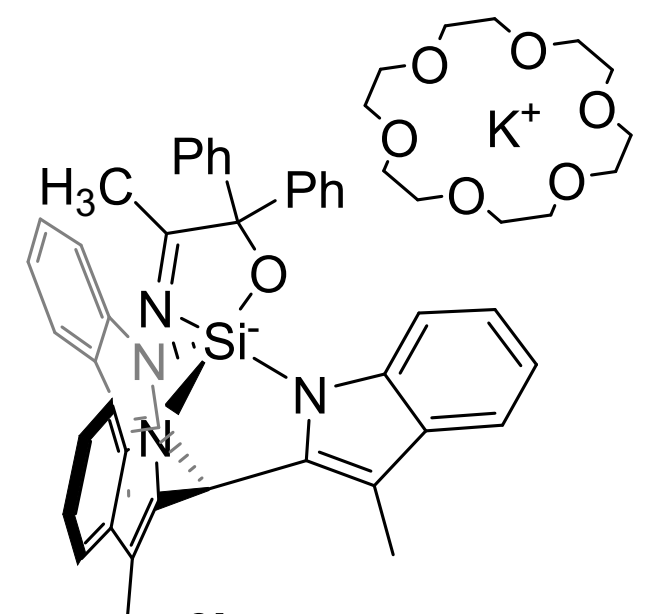

3'

Synthesis of imine cycle 3'. A solution of benzophenone in MeCN (7.6 mg, 0.0417 mmol) in MeCN (1 ml) was added to a suspension of $[K(18\text{-crown-}6)]^+[(tmim)Si]^-$ (30.6 mg, 0.0418 mmol) in MeCN (1ml). The mixture was stirred for 3h, over which time the cloudy white suspension turned to a clear light-yellow solution. The resulting solution was filtered the solvent was remove *in vacuo*, and the resulting off-white powder was dissolved in the minimum amount of MeCN and precipitated using Et_2O. The supernatant was removed, and the precipitate washed with Et_2O. Drying *in vacuo* afforded the product as an off-white powder (35.3 mg, 0.036 mmol, 87%).

^{1}H NMR (400 MHz, CD_3CN, 25°C) δ 7.77 (dt, *J* = 6.3, 1.5 Hz, 4H, Ph_2CO-H_{Ar}), 7.44 – 7.32 (m, 6H, Ph_2CO-H_{Ar}), 7.27 (d, *J* = 7.8 Hz, 3H, Indole-*H*), 6.78 (t, *J* = 7.5 Hz, 3H, Indole-*H*), 6.67 (d, *J* = 7.7 Hz, 3H, Indole-*H*), 5.93 (d, *J* = 1.5 Hz, 1H, Indole-C*H* linker), 3.48 (s, 24H,18-Crown-6), 2.45 (s, 3H NCCH_3), 2.43 (s, 9H, Indole-CH_3), one Indole-*H* probably very broad.

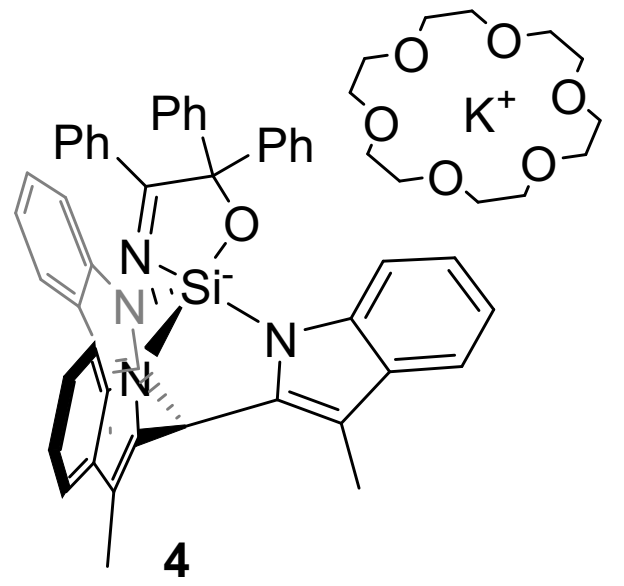

4

Synthesis of Imine cycle with benzonitrile 4. Benzophenone (20.5 mg, 0.113 mmol) was added to a suspension of $[K(18\text{-crown-}6)]^+[(tmim)Si]^-$ (79.9 mg, 0.109 mmol) in THF (3 ml). Benzonitrile (1 ml) was then added to this mixture. The mixture was stirred for 16h, over which time the cloudy white suspension turned to a clear light-yellow solution. The resulting solution was filtered, the solvent was removed *in vacuo*, and the resulting off white powder was dissolved in the minimum amount of MeCN and precipitated using Et_2O. The supernatant was removed, and the precipitate washed with Et_2O. Drying *in vacuo* afforded the product as light-yellow powder (56.4 mg, 0.055 mmol, 50%).

^{1}H NMR (400 MHz, CD_3CN, 25°C) δ 8.10 (d, *J* = 8.3 Hz, 1H, Indole-*H*), 7.81 – 7.72 (m, 4H, Ph_2CO-H_{Ar}), 7.57 (d, *J* = 0.9 Hz, 1H, PhCN-H_{Ar}), 7.55 (t, *J* = 1.6 Hz, 1H, PhCN-H_{Ar}), 7.39 – 7.30 (m, 7H, Ph_2CO-H_{Ar} and PhCN-H_{Ar}), 7.26 (m, *J* = 7.9 Hz, 5H, Indole-*H* and PhCN-H_{Ar}), 6.77 (t, *J* = 7.3 Hz, 3H, Indole-*H*), 6.74 (t, *J* = 6.9 Hz, 1H, Indole-*H*), 6.67 (d, *J* = 8.3 Hz, 2H, Indole-*H*), 6.58 (t, *J* = 7.6 Hz, 1H, Indole-*H*), 6.51 (ddd, *J* = 8.2, 6.8, 1.3 Hz, 2H, Indole-*H*), 5.97 (s, 1H, Indole-C*H* linker), 3.52 (s,24H, 18-crown-6), 2.46 (s, 6H, Indole-CH_3), 2.43 (s, 3H, Indole-CH_3). **^{13}C{^{1}H}-NMR** (APT, 101 MHz, CD_3CN, 25°C) δ 176.3 (s, PhCN), 145.1 (s, Ph_2CO-*C*), 143.3 (s, C_{Indole}), 142.6(s, C_{Indole}), 142.3(s, PhCN-*C*), 140.7(s, C_{Indole}), 140.5(s, C_{Indole}), 131.4(s, CH_{Indole}), 131.3(s, CH_{Indole}), 130.6(s, PhCN-CH_{Ar}), 130.3(s, PhCN-CH_{Ar}), 130.2(s, Ph_2CO-CH_{Ar}), 129.0(s, Ph_2CO-CH_{Ar}), 128.8(s, Ph_2CO-CH_{Ar}), 128.4 (s, Ph_2CO-CH_{Ar}), 120.2(s, CH_{Indole}), 119.6(s, CH_{Indole}), 118.5(s, CH_{Indole}), 117.8(s, CH_{Indole} or PhCN-CH_{Ar}), 117.6(s, CH_{Indole} or PhCN-CH_{Ar}), 117.5(s, CH_{Indole} or PhCN-CH_{Ar}), 114.6(s, CH_{Indole}), 104.5(s, C_{Indole}), 102.9(s, C_{Indole}), 86.9 (s, Ph_2CO) 70.9(s, CH_2,18-crown-6), 32.7(s, CH_{Indole} linker), 8.7(s, $CH_{3\text{-}Indole}$), 8.6(s, $CH_{3\text{-}Indole}$). **^{29}Si{^{1}H}-NMR** (79 MHz, CD_3CN, 25°C) δ –104.35. **ESI-MS$^-$** ($C_{48}H_{37}N_4OSi^-$) m/z calculated 714; found M+$H_2$0 732

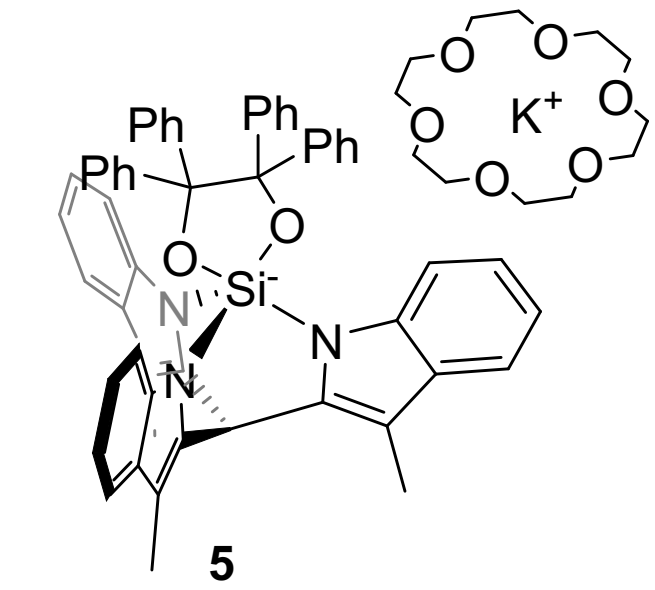

5

Synthesis of 2-sila-1,3-dioxolane 5. Benzophenone (27.7 mg, 0.152 mmol) was added to a suspension of $[K(18\text{-crown-}6)]^+[(tmim)Si]^-$ (60.8 mg, 0.083 mmol) in THF (6 ml). The mixture was stirred for 48h, over time the suspension turns from off-white to white, then n-hexane was added. The supernatant was removed, and the precipitate washed with n-hexane. Drying *in vacuo* afforded the product as a white powder (68 mg, 0.062 mmol, 75%).

^{1}H-NMR (400 MHz, CD_2Cl_2, 25°C) δ 7.92 – 7.82 (m, 8H, Ph_2CO-H_{Ar}), 7.19 (d, *J* = 7.7 Hz, 3H, Indole-*H*), 7.07 – 6.98 (m, 12H, Ph_2CO-H_{Ar}), 6.90 (d, *J* = 8.5 Hz, 3H, Indole-*H*), 6.70 (t, *J* = 7.3 Hz, 3H, Indole-*H*), 6.29 – 6.16 (m, 3H, Indole-*H*), 5.88 (s, 1H, Indole-C*H* linker), 3.18 (s, 24H, 18-crown-6), 2.42 (s, 9H, Indole-CH_3). **^{13}C{^{1}H}-NMR** (APT, 101 MHz, CD_2Cl_2, 25°C) δ 147.6(s, Ph_2CO-*C*), 142.9(s, C_{Indole}), 140.9(s, C_{Indole}), 131.0(s, Ph_2CO-CH_{Ar}), 130.9(s, C_{Indole}), 127.1(s, Ph_2CO-CH_{Ar}), 126.3(s, Ph_2CO-CH_{Ar}), 118.2(s, CH_{Indole}), 118.0(s, CH_{Indole}), 117.1(s, CH_{Indole}), 116.5(s, CH_{Indole}), 103.2(s, C_{Indole}), 89.3 (Ph_2CO), 70.2 (s, CH_2,18-crown-6), 32.3 (s, CH_{Indole} linker), 8.8(s, $CH_{3\text{-}Indole}$). **^{29}Si{^{1}H}-NMR** (79 MHz, CD_3CN, 25°C) δ -118.4. **ESI-MS$^-$** ($C_{54}H_{42}N_3O_2Si^-$) m/z calculated 793; found 793.

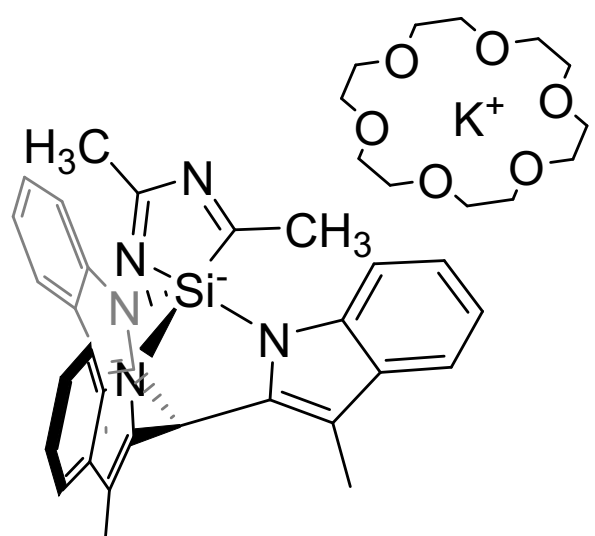

6

Synthesis of the cyclic diazasilole 6 A suspension of $tmimSi^-K^+$[18-crown-6] (45.8 mg, 0.063 mmol) in MeCN (6 ml) was left stirring for for 48h. Then the mixture was filtered. Drying *in vacuo* afforded the product as a yellow powder (33.6mg, 0.041 mmol, 65%).

^{1}H-NMR (400 MHz, CD_3CN, 25°C) δ 7.42 (ddt, J = 6.2, 4.0, 2.0 Hz, 3H, Indole-*H*), 7.25 – 7.18 (m, 3H, Indole-*H*), 7.04 – 6.96 (m, 6H, Indole-*H*), 6.39 (s, 1H, Indole-C*H* linker), 3.47 (s, 24H,18-crown-6), 2.54 (s, 3H, Cycle-CH_3), 2.46 (s, 9H, Indole-CH_3), 2.38 (s, 3H, Cycle-CH_3). **^{13}C{^{1}H}-NMR** (APT, 101 MHz, CD_3CN, 25°C) δ 182.2 (s, Cycle *C*=N), 178.7 (s, Cycle *C*=N), 139.7(s, C_{Indole}), 137.9(s, C_{Indole}), 131.6(s, C_{Indole}), 122.3 (s, CH_{Indole}), 120.5 (s, CH_{Indole}), 119.2(s, CH_{Indole}), 113.4 (s, CH_{Indole}), 71.1(s, CH_2,18-crown-6), 32.8(s, CH_{Indole} linker), 30.5(s, Cycle CH_3), 29.7(s, Cycle CH_3), 9.3(s, $CH_{3\text{-}Indole}$) ($C_{Indole\ at}$ 108.5 ppm detected via HMBC measurement).

2. Additional Experiments

2.1. Imine-Enamine Tautomerization

The product of the reaction of compound 1 with benzophenone and MeCN or MeCN-d$_3$ gave the imine **3'** or **3''** that are isolated if the reaction is stopped after 3h. The comparison of the NMR of **3'**and **3''** shows the absence of the deuterated CH$_3$ of the imine in **3**'' (Figure S1).

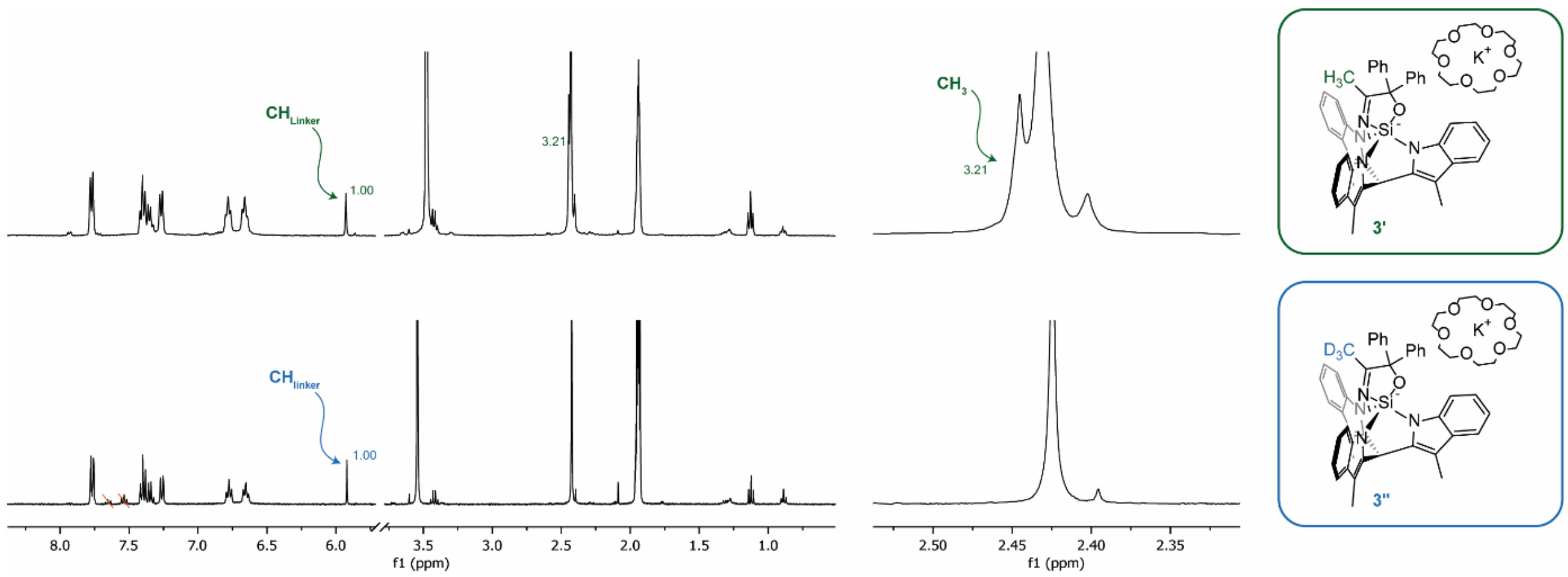

Figure S1. 1H-NMR (400 MHz) spectra of compounds **3'** and **3''** in CD$_3$CN at 25°C. Impurities: 1.12 and 3.42 ppm – Et$_2$O, in the aromatic region of the spectra of compound 3'' between 7.75 and 7.50– benzophenone.

It was observed that the imine **3'** slowly tautomerizes to the enamine **3** over time at RT (Scheme S1 and Figure S2).

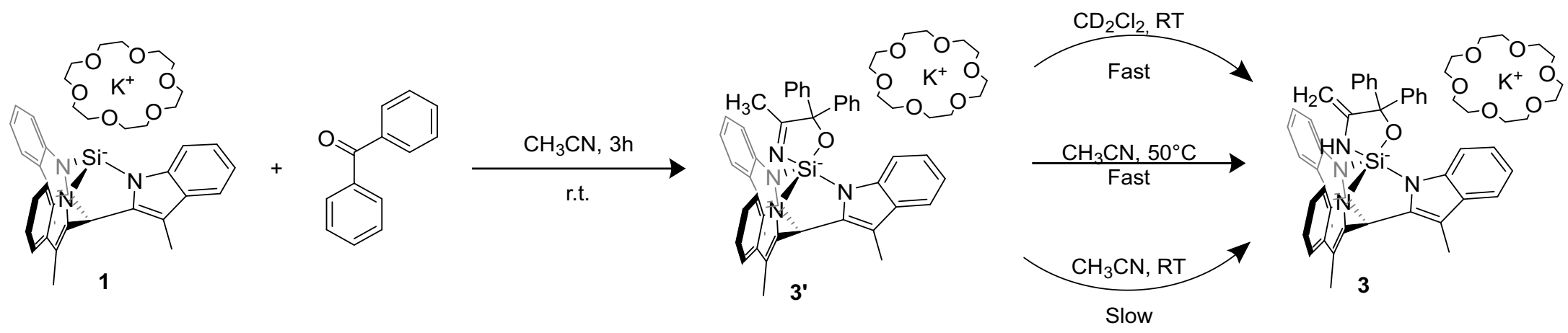

Scheme S1. Tautomerization of compound **3' (imine)** to **3(enamine)**.

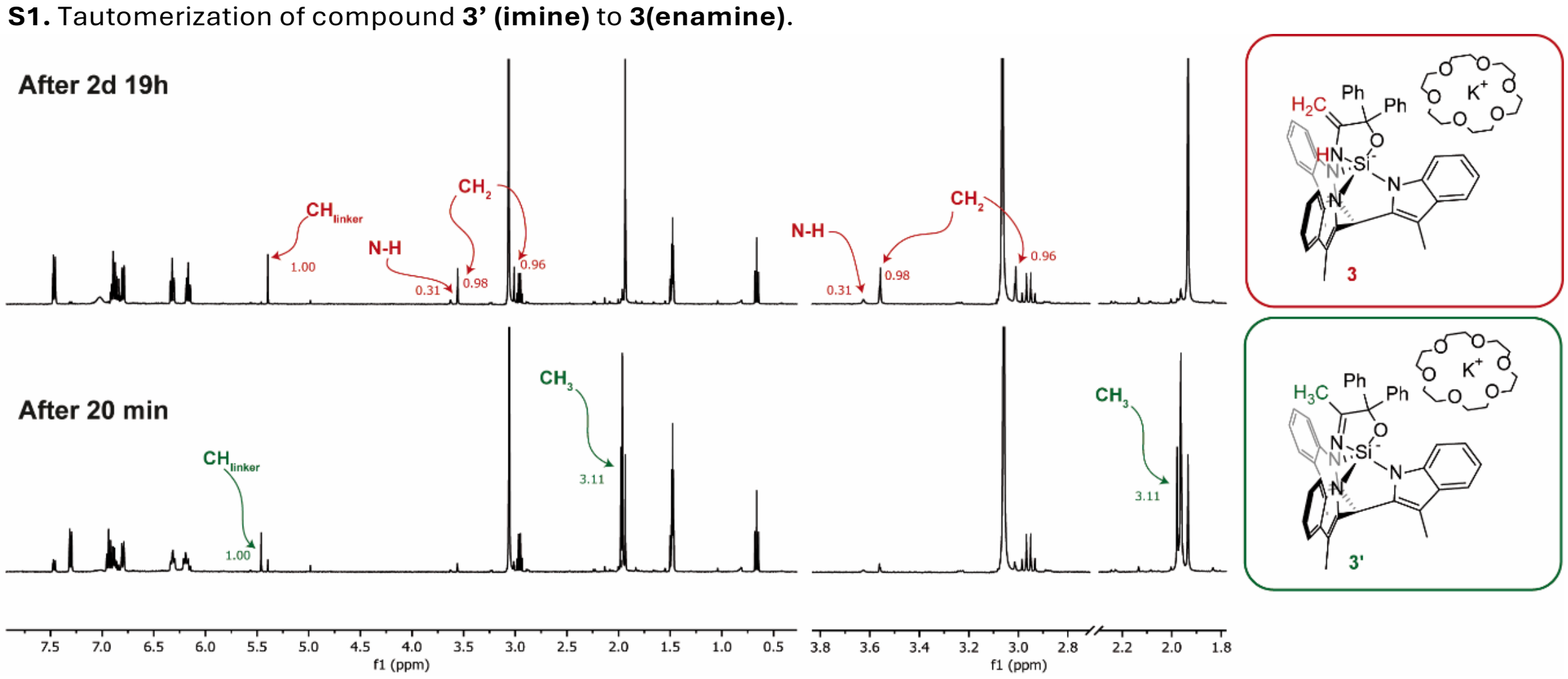

Figure S2. Comparison of ^{1}H-NMR (400 MHz) spectrum of compound **3** and **3'** in CD$_3$CN at 25°C. Impurities: 1.12 and 3.42 ppm – Et$_2$O.

The -NH signal of enamine **3** was identified using HSQC measurements in MeCN-d$_3$ and DCM-d$_2$, where its signal not bonded to any carbon can be observed (figure S3).

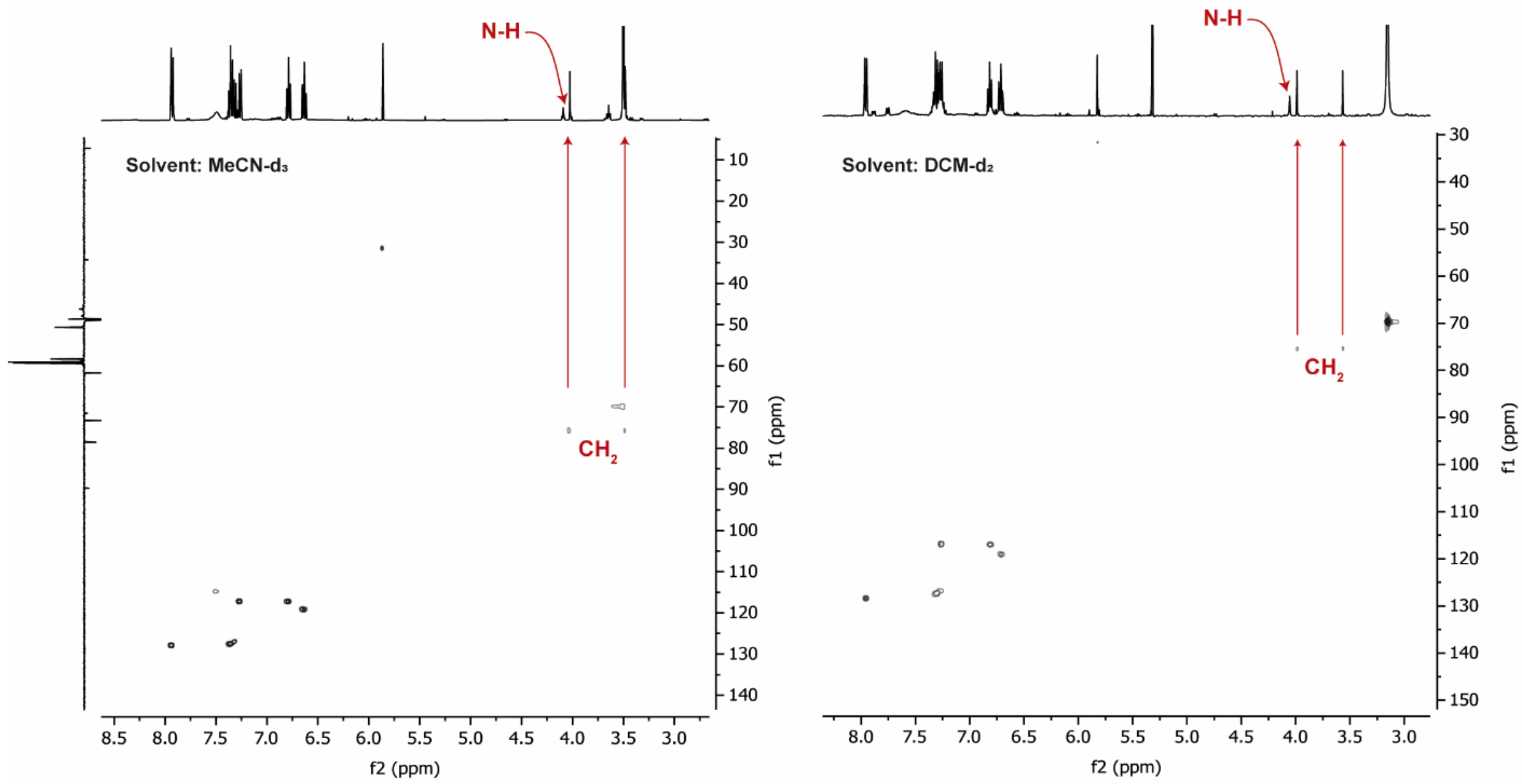

Figure S3. ^{1}H-^{13}C HSQC spectra of **3** in MeCN-d$_3$ and DCM-d$_2$.

The rate of tautomerization can be increased heating the sample (Figure S4).

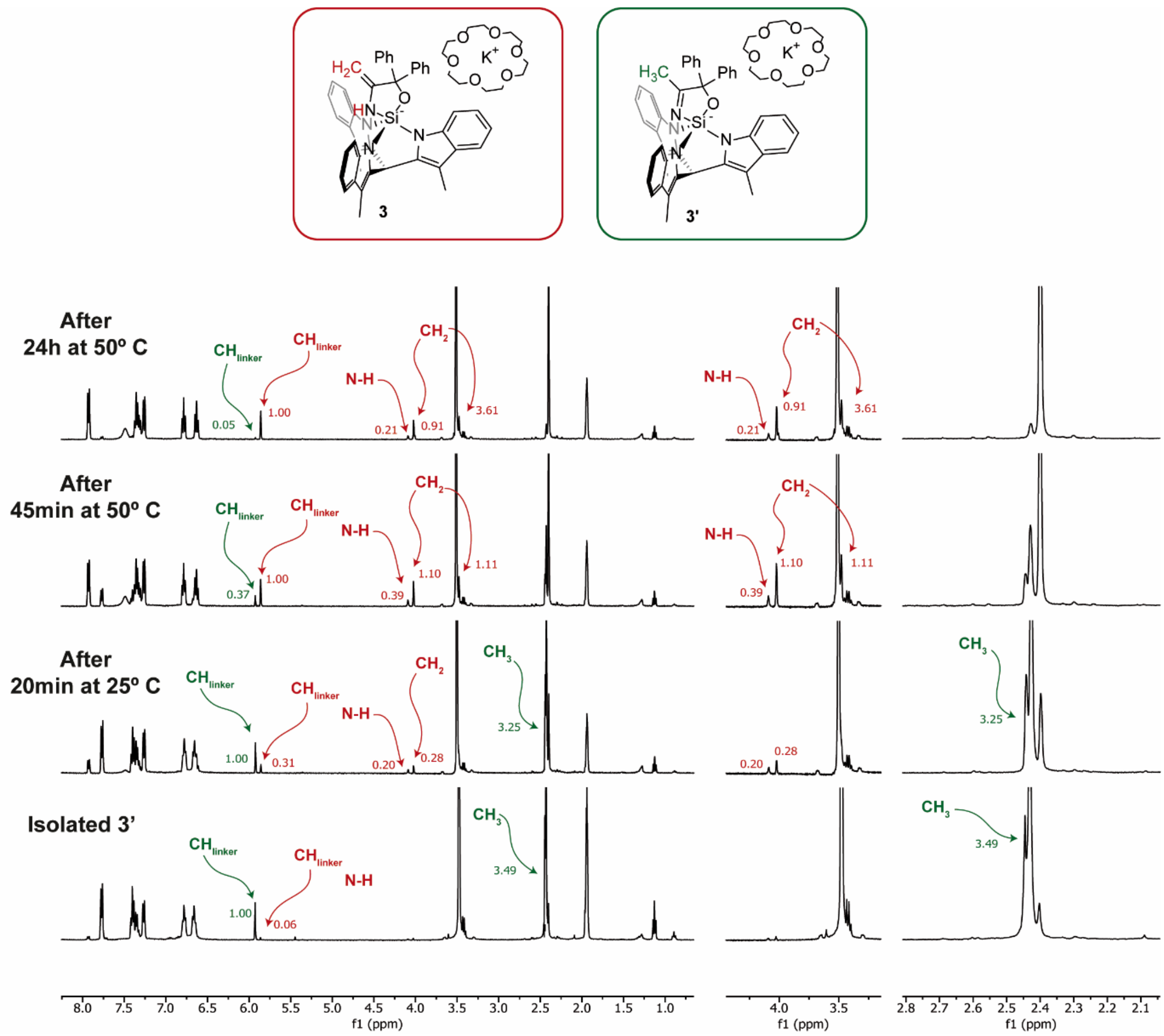

Figure S4. ^{1}H-NMR spectra of **3'** in MeCN-d$_3$.

If **3'** is dissolved in DCM (or DCM-d$_2$) moves the tautomerization towards **3**, ending up to a 1:0.25 ratio (Figure S5).

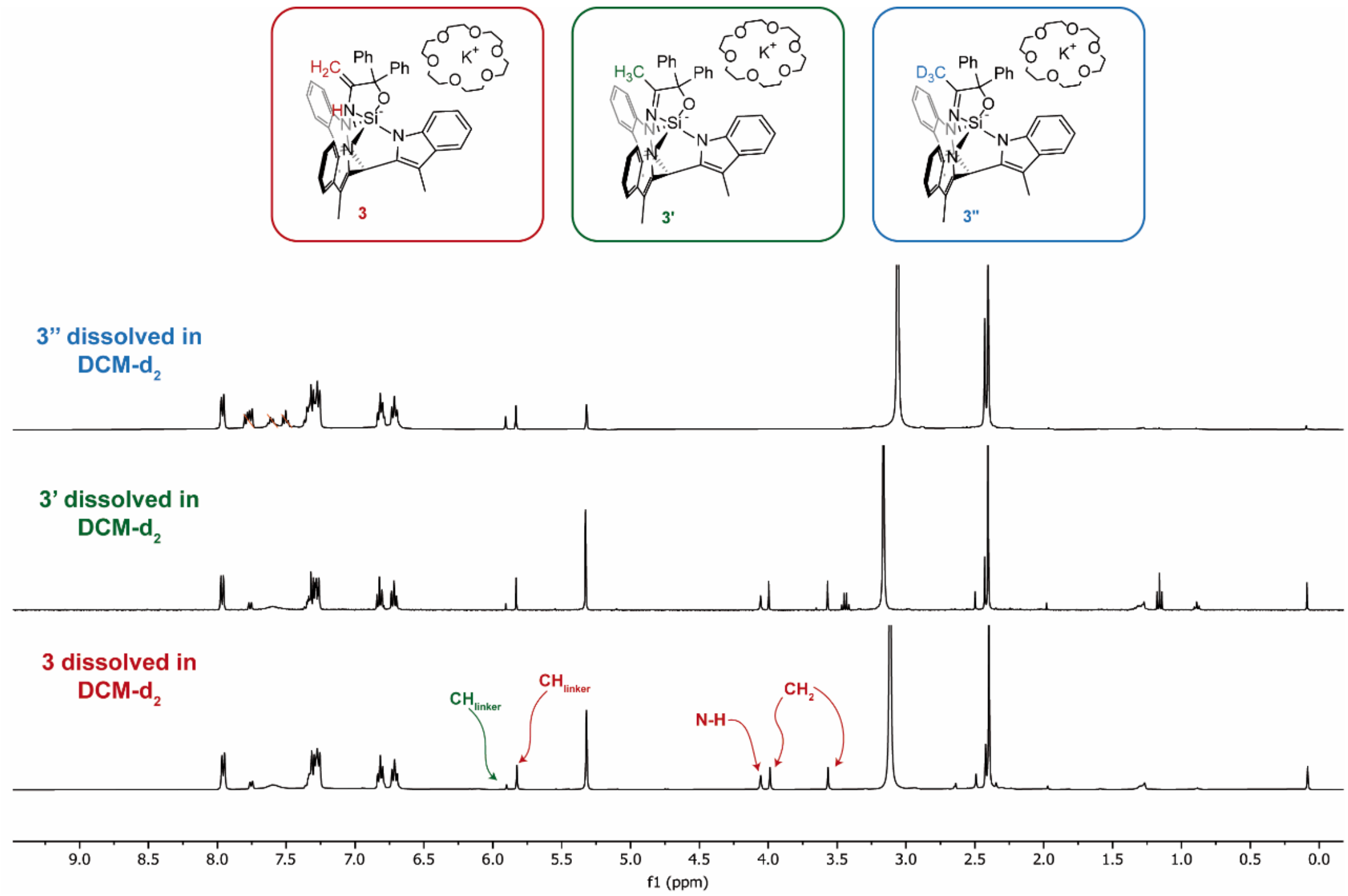

Figure S5. Comparison of ^{1}H-NMR (400 MHz) spectrum of compound **3**, **3'** and **3''** in DCM-d_2.

2.2. Synthesis of the Imine Cycle 3' Using $[K(2.2.2\text{-cryptand})]^+[tmimSi]^-$

To verify if the potassium cation is involved in the mechanism for the formations of the 5-membered cycles, the reaction was performed using an analogue of **1** in which the potassium counterion is bound to a 2.2.2-cryptand instead 18-crown-6 (ref), which encapsulates the potassium cation and renders it less accessible. The reaction was performed for 3h, then the product was isolated. The experiment showed that the lack of accessibility of the potassium does not hinder the formation of the product, suggesting that the reaction is not aided by the potassium cation (Scheme S2 and Figure S6). The reaction proceeds more slowly due to the very low solubility of the cryptand cage.

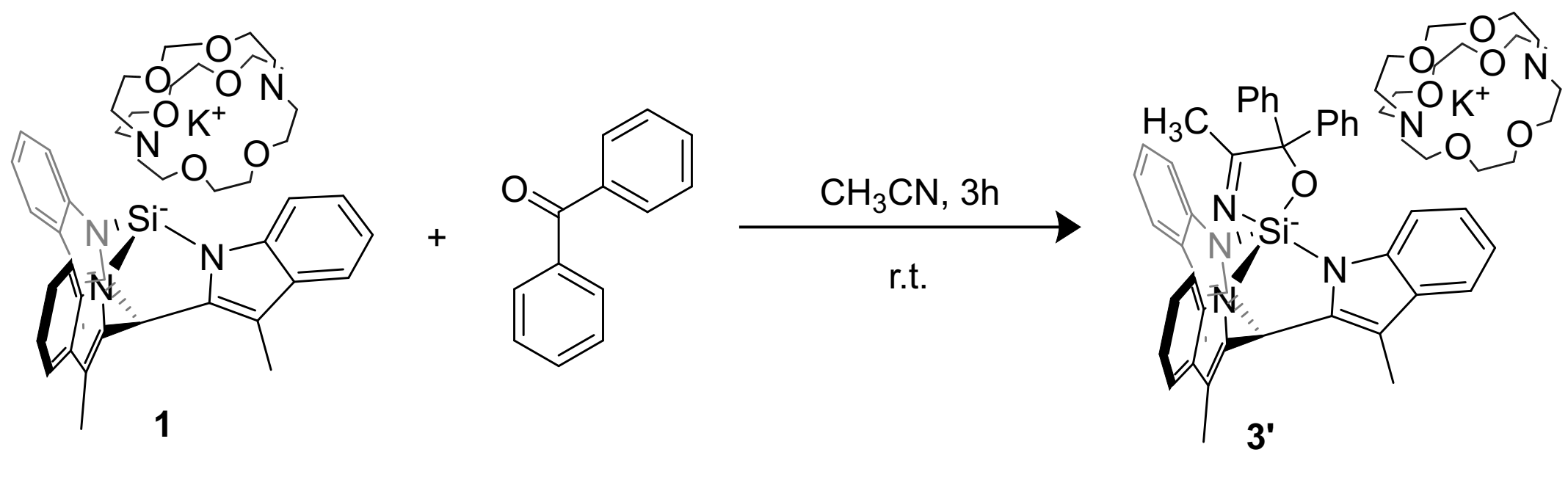

Scheme S2 Reaction of the cryptand cage with 1 eq of benzophenone in MeCN.

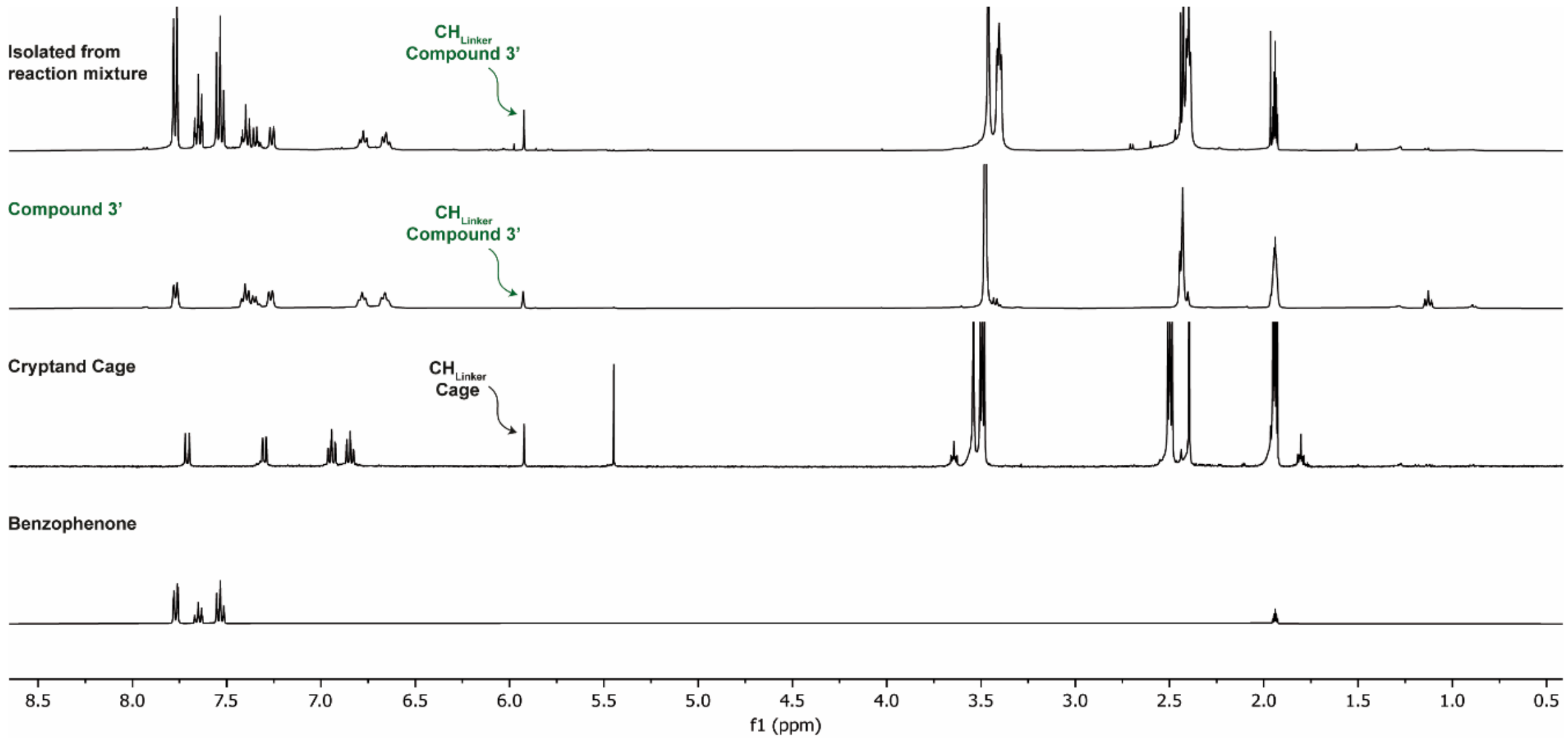

Figure S6. Comparison of ^{1}H-NMR spectra of benzophenone, the free cryptand cage, compound **3'**and the product isolated from the reaction of the free cryptand cage with 1 eq of benzophenone in MeCN.

2.3. Synthesis of the Enamine Cycle using 1 eq of MeCN

When the reaction of **1** with 1eq of benzophenone is performed using 1eq of MeCN in THF for 3h, a mixture of products that include compound **3'**, **3** and **5** is formed (Figure S7). The reaction mixture was dried and the solid was extracted first with THF then with MeCN.

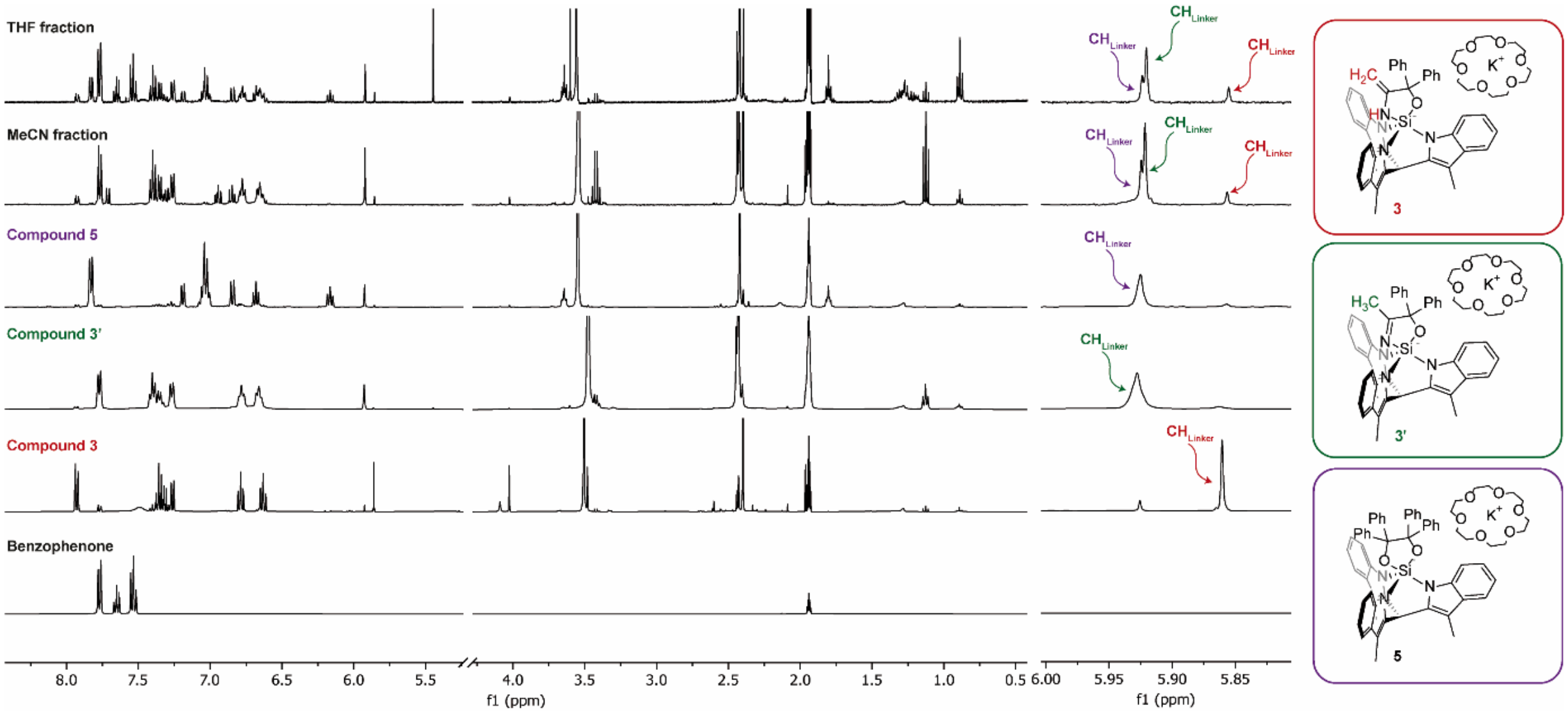

Figure S7. Comparison of ^{1}H-NMR (400 MHz) spectra of benzophenone, compound **3**, compound **3'**, compound **5** and samples obtained from extraction of the dried reaction mixture with THF and then with MeCN in CD_3CN at 25°C.

2.4. VT-NMR of the Enamine Product 3 in DCM-d_2

The fluxional process of compound **3** was investigated by variable-temperature ^{1}H-NMR spectroscopy. In DCM-d_2, the spectrum shows a series signals, which belong to the tmim fragment, with one signal that is very broad and split into two signals as the temperature is lowered (Figure S8, S9, S10 and S11).

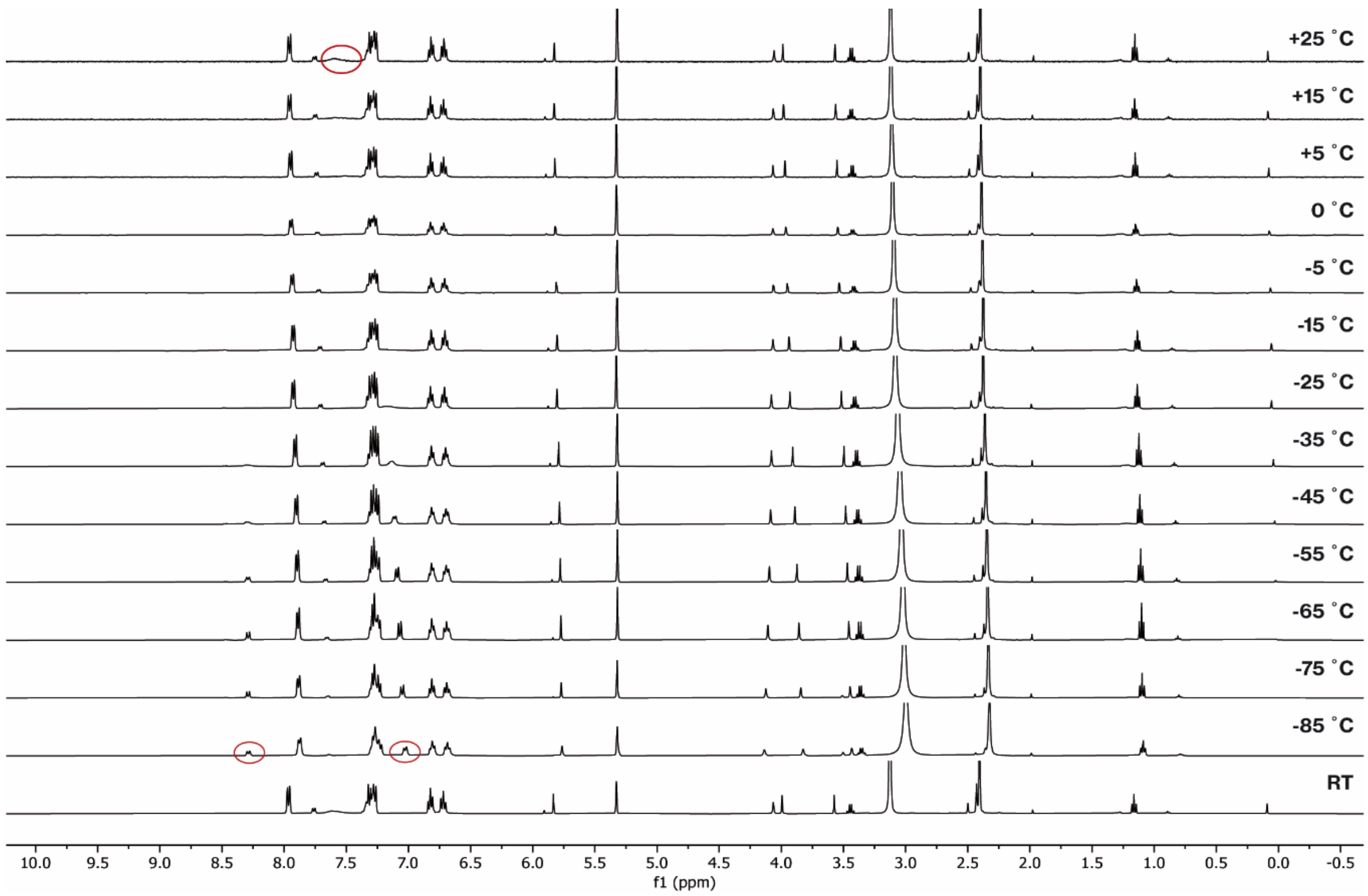

Figure S8. Variable-temperature ^{1}H-NMR (400 MHz) spectra of compound **3** (from –85°C to +25°C) measured in DCM-d_2.

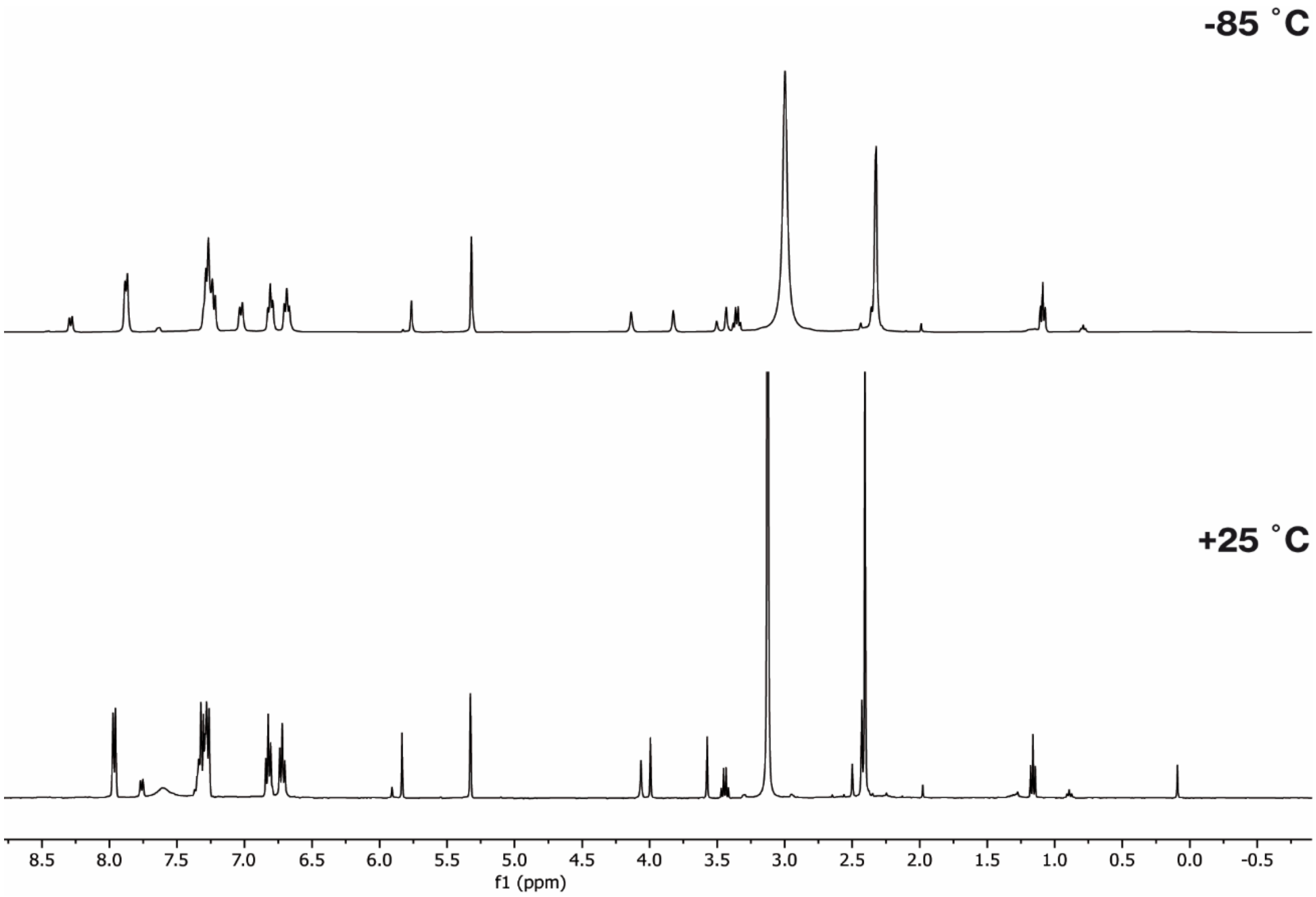

Figure S9. Comparison of the ^{1}H-NMR spectra of compound **3** at +25°C and -80°C.

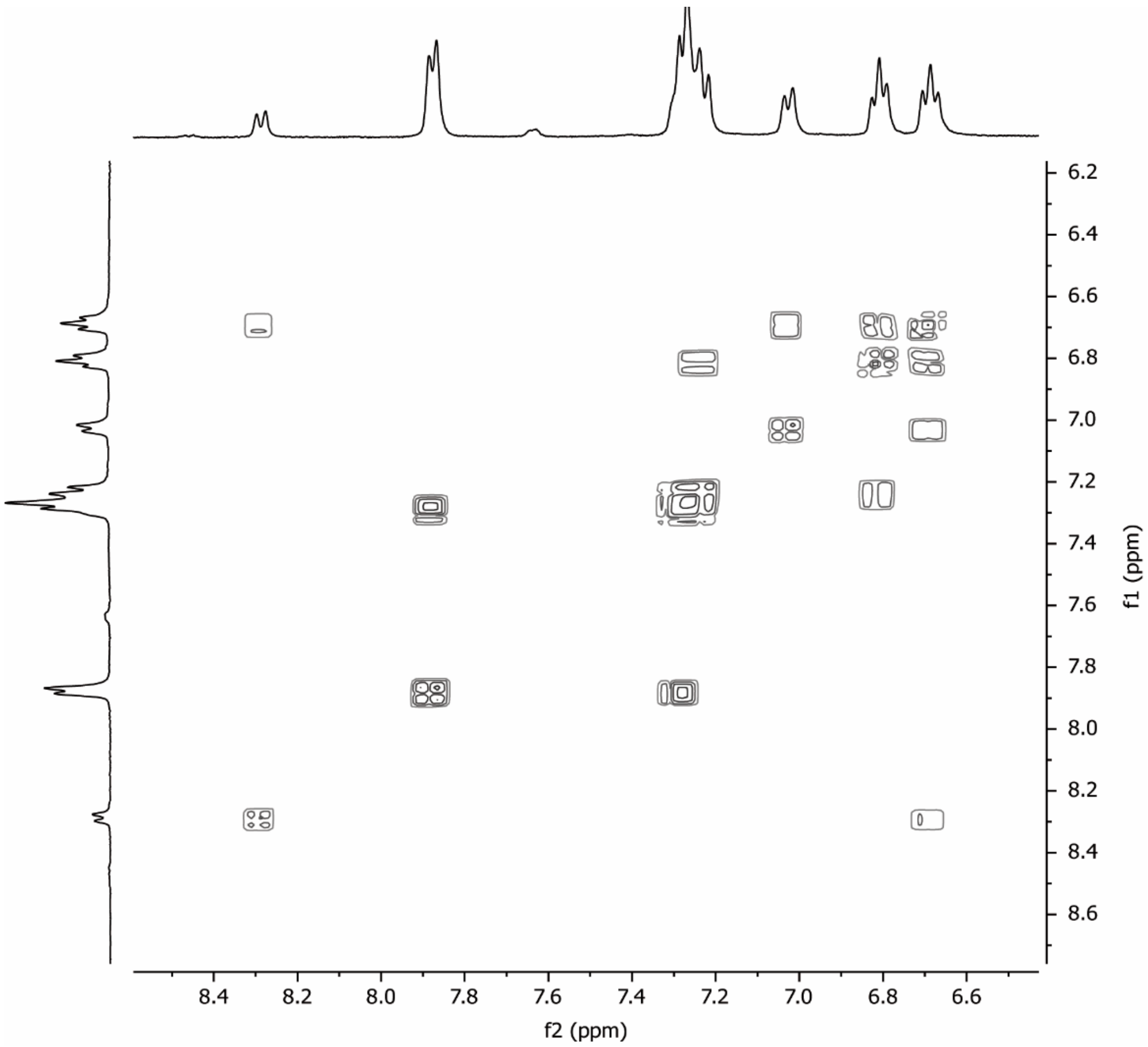

Figure S10. ^{1}H-COSY (400 MHz) spectrum of compound **3** in CD_3CN at -85°C

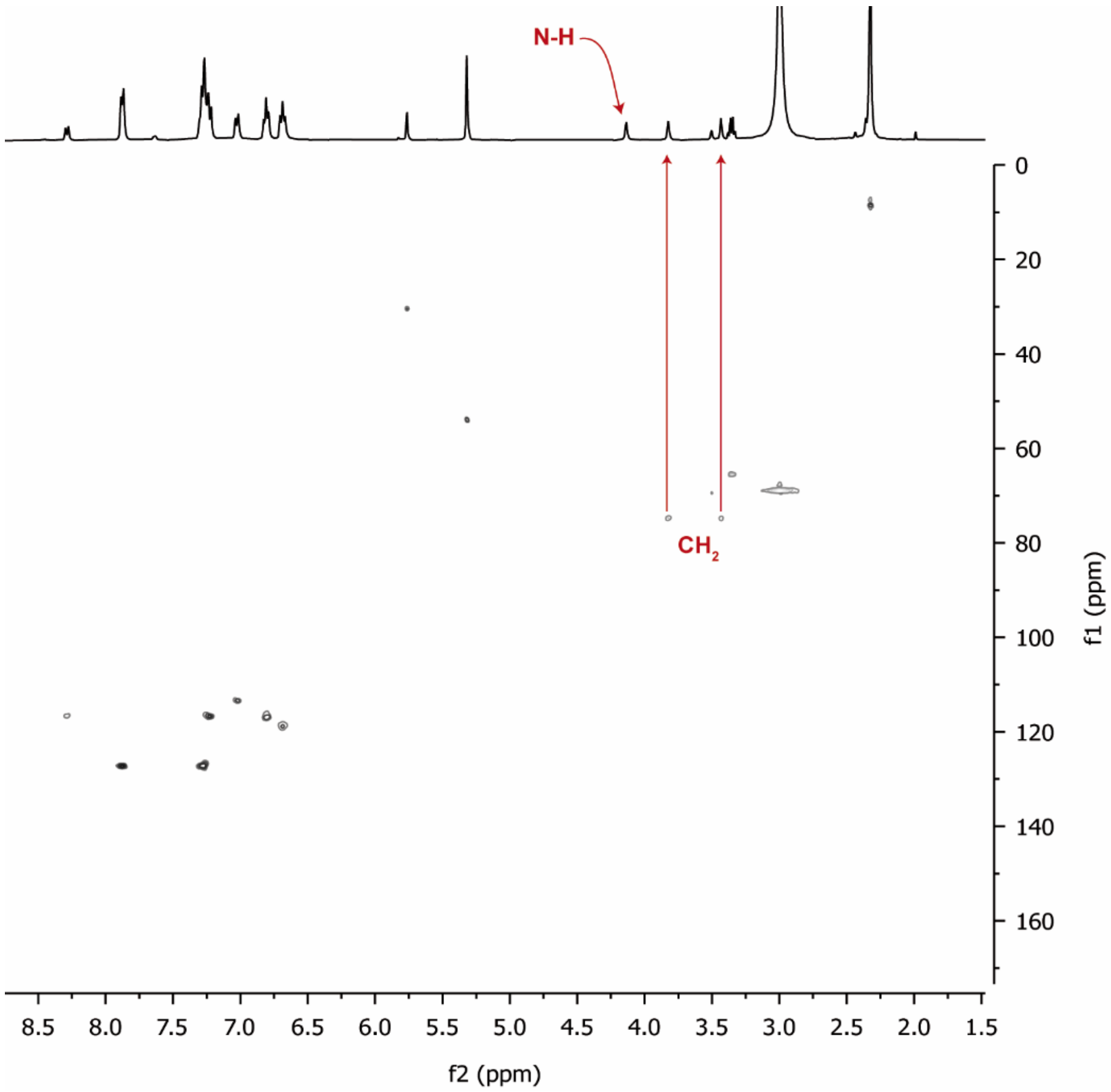

Figure S11. ^{1}H-^{13}C HSQC spectrum of compound **3** in CD_3CN at -85°C.

2.5. Reaction of Compound 1 with HBpin in CD_3CN

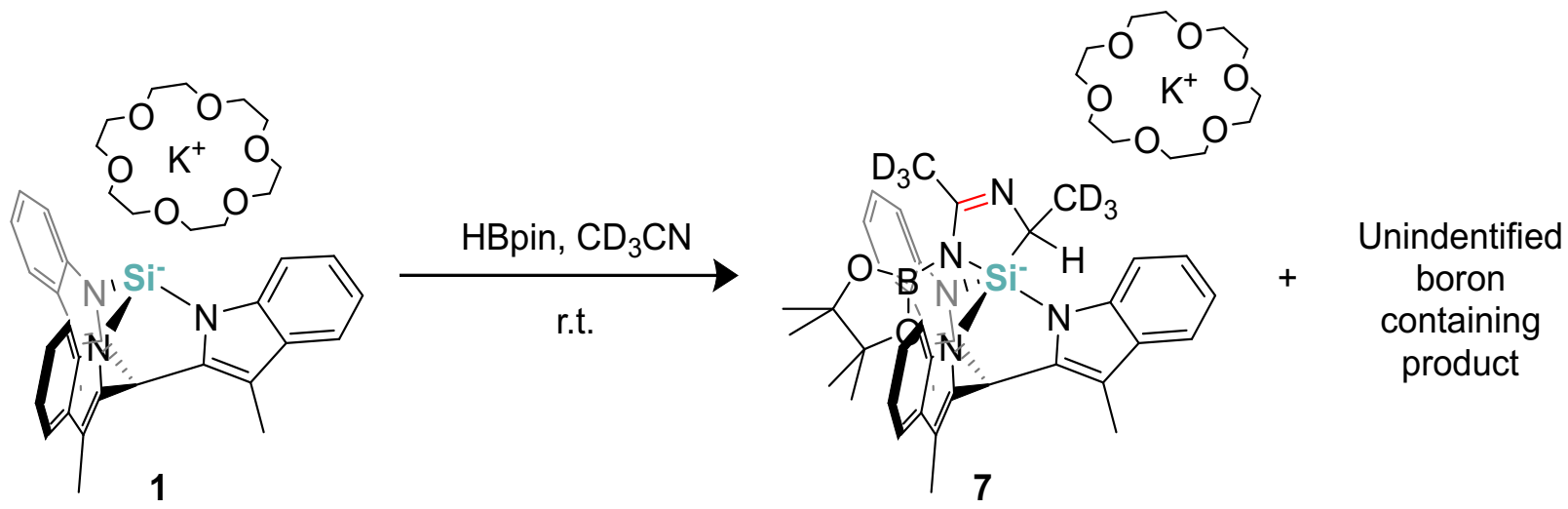

Scheme S3. Reaction of compound **1** with HBpin in MeCN-d_3.

A small amount of a crystalline derivative **7** were obtained from the reaction of compound **1** with HBpin in MeCN-d_3. Monitoring the in-situ reaction revealed the formation of a mixture of products (Figure S12) of which one (7) deposited x-ray quality crystals over time (see section 3.3). The crystal structure of **7** showed a 5-membered heterocycle that would be the result of a formal hydroboration of **6** and confirms the head to tail condensation of two acetonitrile molecules.

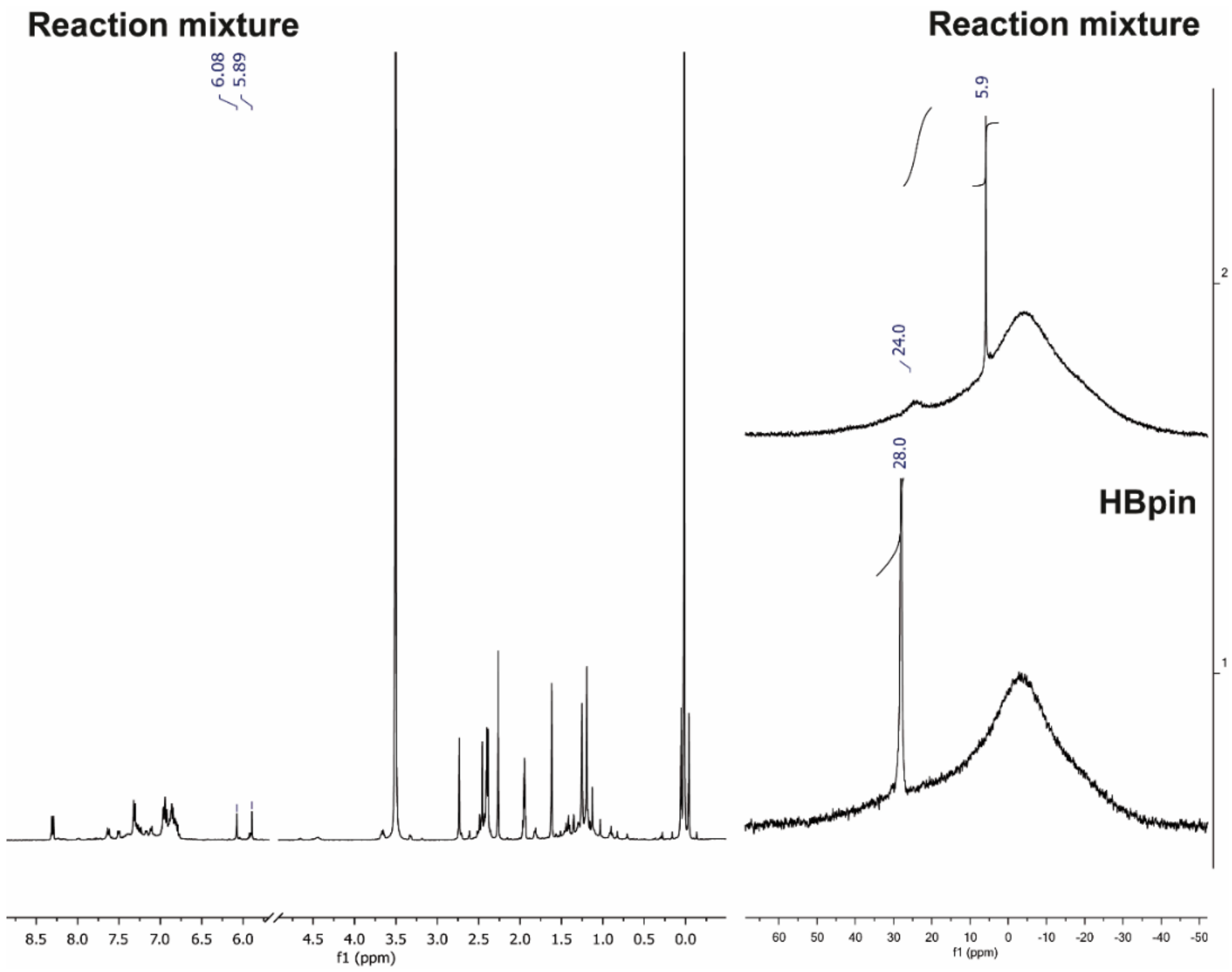

Figure S12. ^{1}H-NMR spectrum of the reaction mixture of **1** with HBpin in CD_3CN and ^{11}B-spectrum of the reaction mixture and HBpin in CD_3CN.

2.6. IR Analysis

ATR-FTIR analysis of compounds **3**, **4**, **5** and **6** (Figure S13, S14 and S15 and S16) were performed.

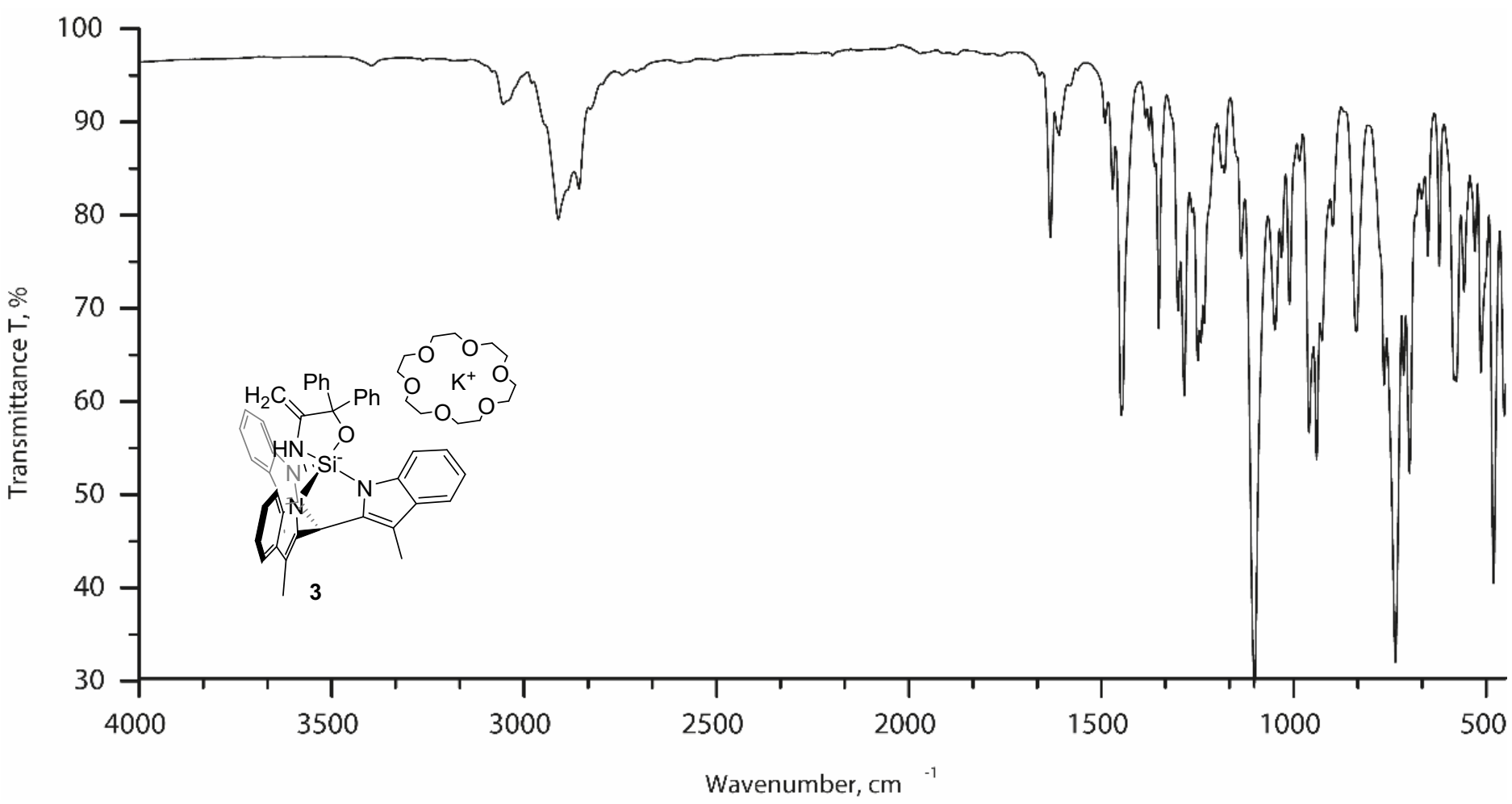

Figure S13. IR spectrum of compound **3**

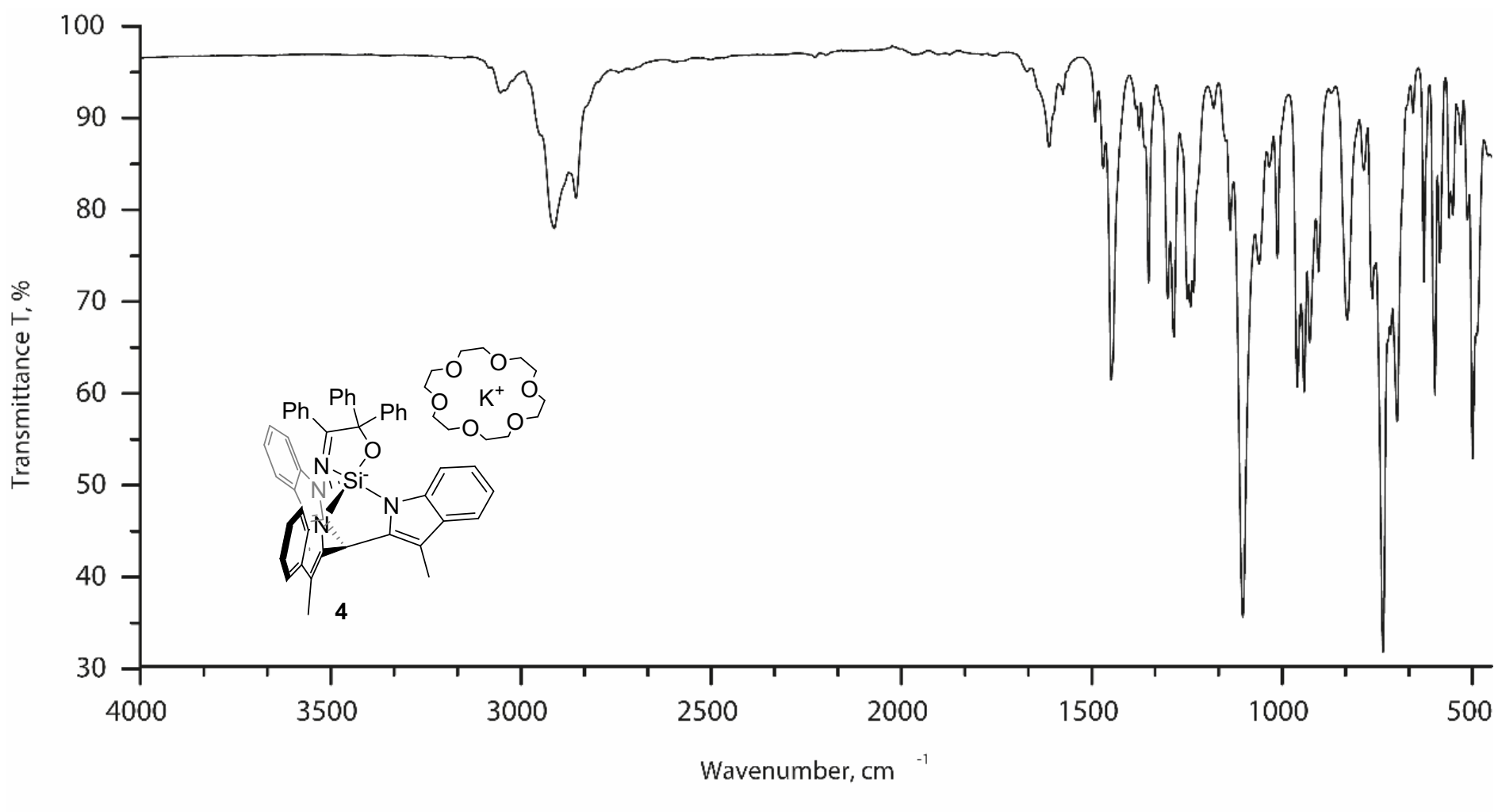

Figure S14. IR spectrum of compound **4**

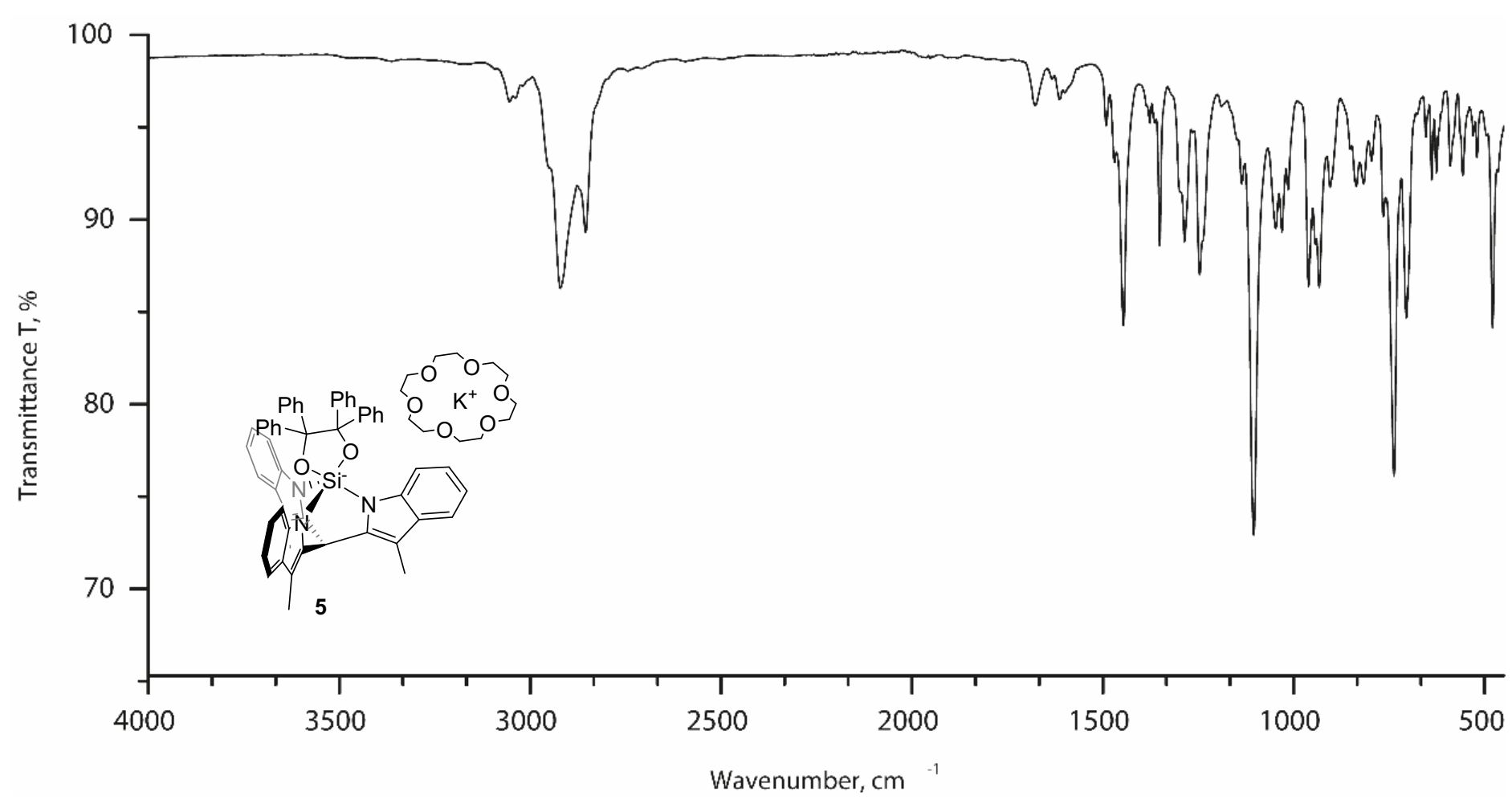

Figure S15. IR spectrum of compound **5**

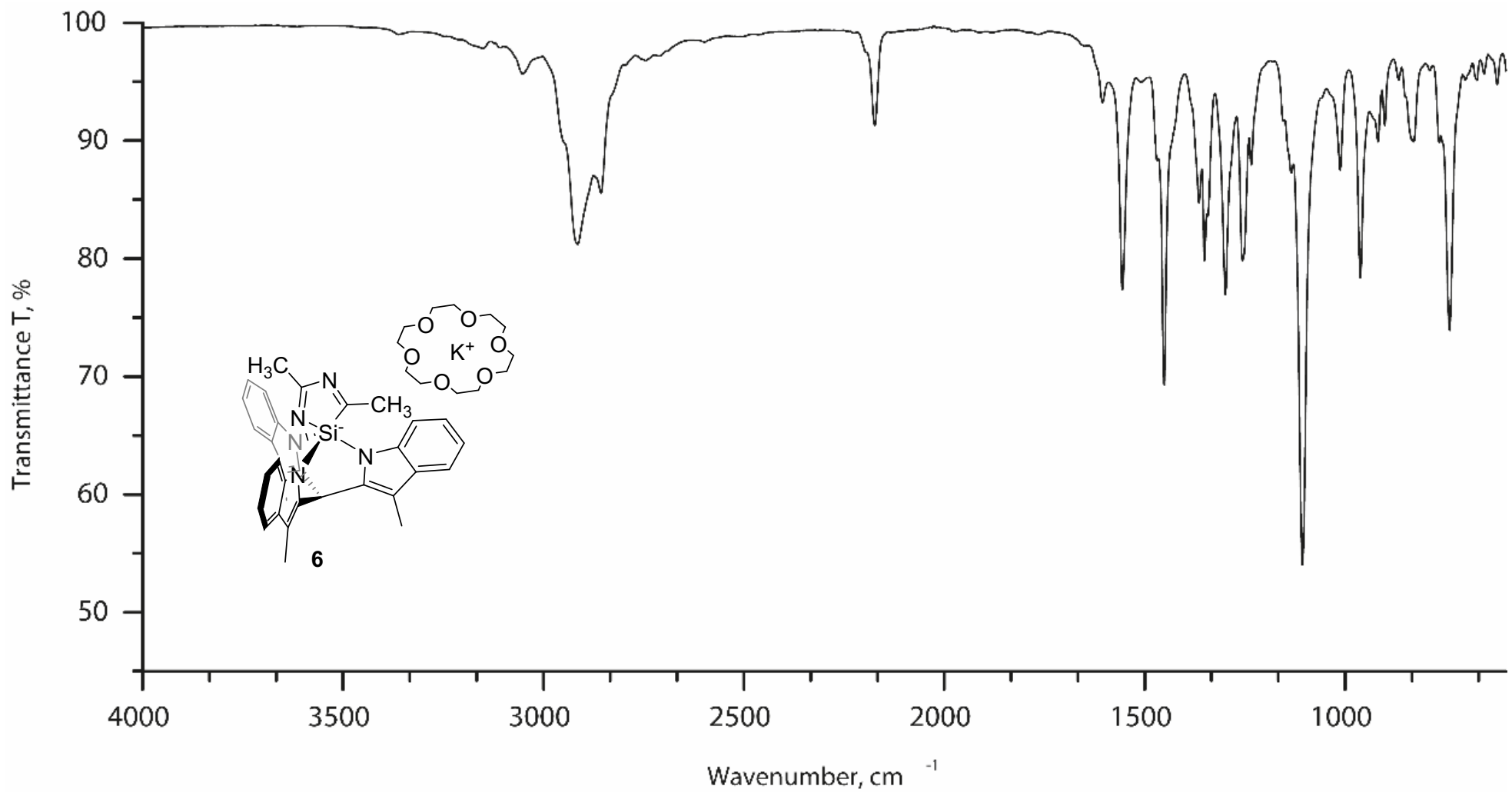

Figure S16. IR spectrum of compound **6,** the signal at 2173 cm^{-1} can be assigned to residual acetonitrile.

3. X-Ray Crystal Structure Determinations

2564771 (for **2**), 2564772 (for **3**), and 2564773 (for **7**) contain the supplementary crystallographic data for this paper. These data can be obtained free of charge from The Cambridge Crystallographic Data Centre via www.ccdc.cam.ac.uk/data_request/cif.

3.1. X-ray crystal Structure Determination of Compound 2

$C_{49}H_{55}KN_4O_7Si$, Fw = 879.16, colourless block, 0.36 × 0.21 × 0.06 mm^3, monoclinic, $P2_1/c$ (no. 14), a = 13.1842(5), b = 20.8663(9), c = 17.0273(9) Å, β = 103.320(2)°, V = 4558.3(3) $Å^3$, Z = 4, D_x = 1.281 g/cm^3, μ = 0.20 mm^{-1}. The diffraction experiment was performed on a Bruker Kappa ApexII diffractometer with sealed tube and Triumph monochromator (λ = 0.71073 Å) at a temperature of 150(2) K up to a resolution of $(\sin \theta/\lambda)_{max}$ = 0.61 $Å^{-1}$. The Eval15 software[4] was used for the intensity integration. The prediction of the reflection profiles involved a large isotropic mosaicity of 1.6°. A multi-scan absorption correction and scaling was performed with SADABS[5] (correction range 0.64-0.75). A total of 79944 reflections was measured, 8471 reflections were unique (R_{int} = 0.052), 6346 reflections were observed [I>2σ(I)]. The structure was solved with dual-space methods using SHELXT[6]. Structure refinement was performed with SHELXL-2018[7] on F^2 of all reflections. Non-hydrogen atoms were refined freely with anisotropic displacement parameters. The silicon-bound benzaldehyde was refined with a disorder model. Hydrogen atoms were introduced in calculated positions and refined with a riding model. 636 Parameters were refined with 176 restraints (geometry and displacement parameters of disordered groups). R1/wR2 [I > 2σ(I)]: 0.0458 / 0.1101. R1/wR2 [all refl.]: 0.0688 / 0.1252. S = 1.041. Residual electron density between -0.29 and 0.35 e/$Å^3$. Geometry calculations and checking for higher symmetry was performed with the PLATON program[8].

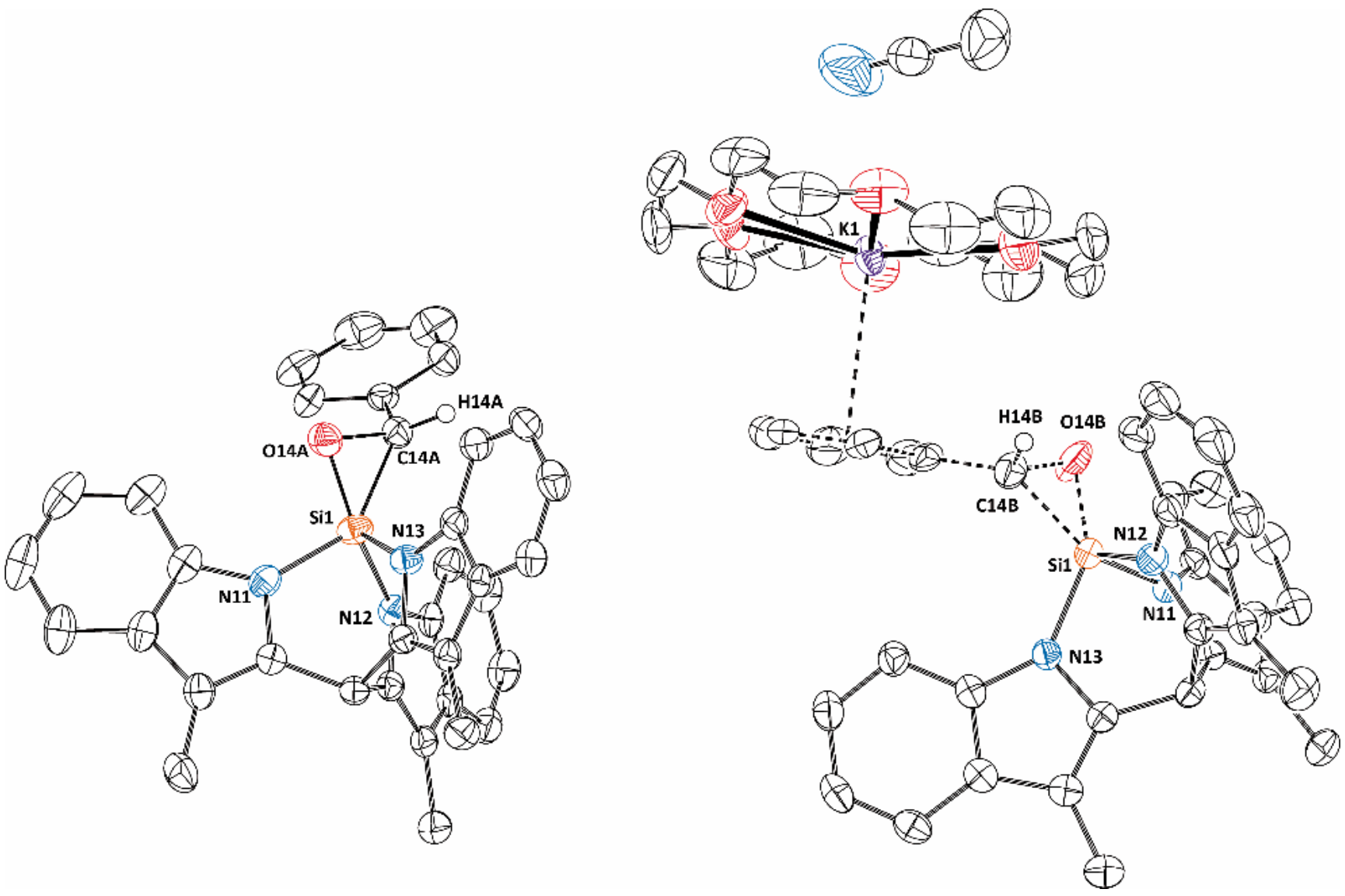

Figure S17. Molecular structure of **2** in the crystal (displacement ellipsoids are drawn at the 50% probability level). C-H hydrogen atoms are omitted for clarity. Only the major component of the disordered silicon-bound benzaldehyde is shown.

3.2. X-ray crystal Structure Determination of Compound 3

$[C_{16}H_{30}KN_2O_6][C_{43}H_{35}N_4OSi] \cdot 2(CH_3CN)$, Fw = 1119.46, colourless plate, 0.47 × 0.33 × 0.03 mm^3, monoclinic, $P2_1/c$ (no. 14), a = 13.5320(6), b = 23.3670(10), c = 19.3410(9) Å, β = 97.773(2) °, V = 6059.5(4) $Å^3$, Z = 4, D_x = 1.227 g/cm^3, μ = 0.16 mm^{-1}. The diffraction experiment was performed on a Bruker Kappa ApexII diffractometer with sealed tube and Triumph monochromator (λ = 0.71073 Å) at a temperature of 150(2) K up to a resolution of $(\sin \theta/\lambda)_{max}$ = 0.65 $Å^{-1}$. The crystal appeared to be non-merohedrally twinned with a twofold rotation about *hkl* = (0,0,1) as twin operation. Consequently, two orientation matrices were used for the intensity integration with the Eval15 software[4]. A multi-scan absorption correction and scaling was performed with TWINABS[9] (correction range 0.68-0.75). A total of 52718 reflections was measured, 13754 reflections were unique (R_{int} = 0.087), 8256 reflections were observed [I>2σ(I)]. The structure was solved with dual-space methods using SHELXT[6]. Structure refinement was performed with SHELXL-2018[7] on F^2 of all reflections. Non-hydrogen atoms were refined freely with anisotropic displacement parameters. One phenyl group was refined with a disorder model. The N-H hydrogen atom was located in difference Fourier maps and refined freely with an isotropic displacement parameter. C-H hydrogen atoms were introduced in calculated positions and refined with a riding model. 788 Parameters were refined with 163 restraints (geometry and displacement parameters of disordered phenyl group). R1/wR2 [I > 2σ(I)]:

0.0598 / 0.1138. R1/wR2 [all refl.]: 0.1199 / 0.1329. S = 1.016. Twin fraction BASF = 0.2335(9). Residual electron density between -0.31 and 0.28 e/Å^3. Geometry calculations and checking for higher symmetry was performed with the PLATON program[8].

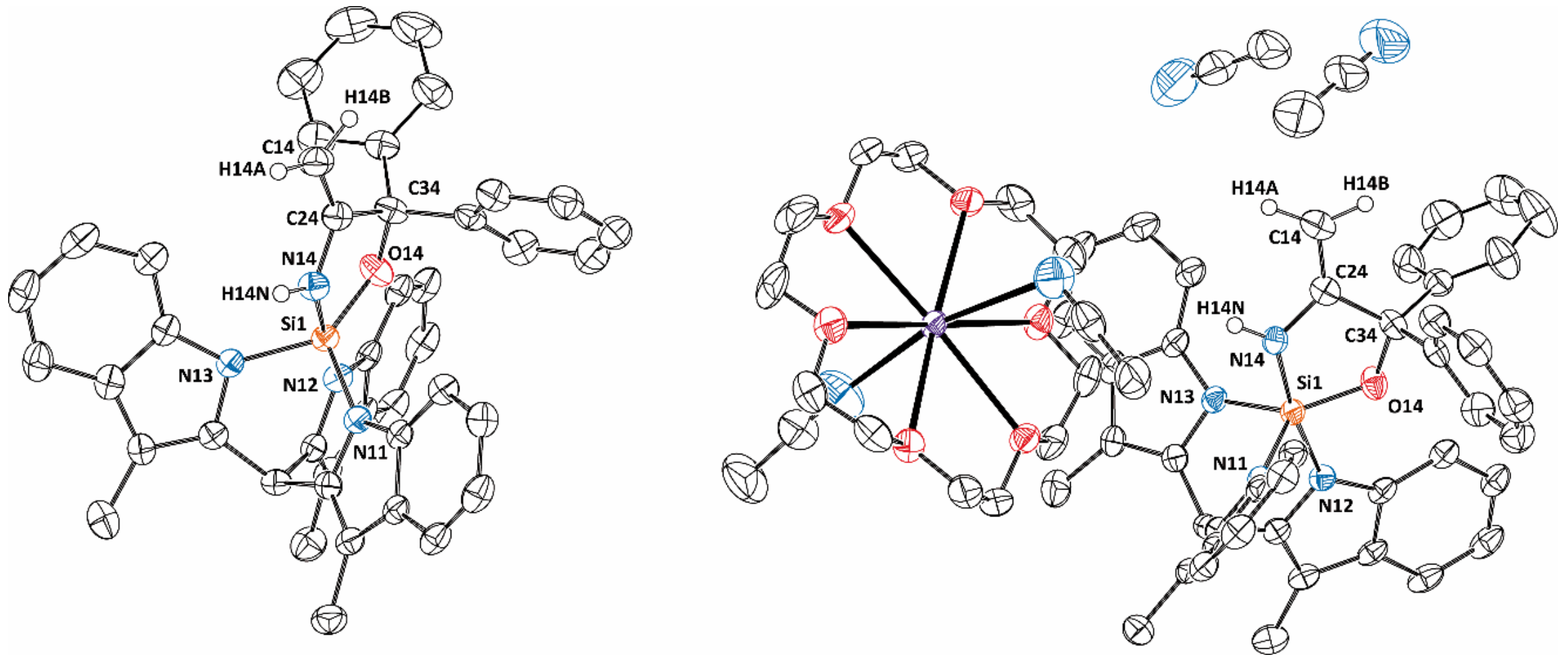

Figure S18. Molecular structure of **3** in the crystal (displacement ellipsoids are drawn at the 50% probability level). C-H hydrogen atoms are omitted for clarity.

3.3. X-ray crystal Structure Determination of Compound 7

$C_{50}H_{65}BKN_5O_8Si$, Fw = 942.07, colourless block, 0.44 × 0.36 × 0.25 mm^3, monoclinic, $P2_1/n$ (no. 14), a = 13.5035(9), b = 22.5395(11), c = 16.6983(14) Å, β = 101.111(3)°, V = 4987.1(6) Å^3, Z = 4, D_x = 1.255 g/cm^3, μ = 0.19 mm^{-1}. The diffraction experiment was performed on a Bruker Kappa ApexII diffractometer with sealed tube and Triumph monochromator (λ = 0.71073 Å) at a temperature of 150(2) K up to a resolution of $(\sin \theta/\lambda)_{max}$ = 0.61 Å^{-1}. The crystal appeared to be cracked into two fragments with an angle of 3.9° between them. Consequently, two orientation matrices were used for the intensity integration with the Eval15 software[4]. A multi-scan absorption correction and scaling was performed with TWINABS[9] (correction range 0.53-0.75). A total of 60334 reflections was measured, 9337 reflections were unique (R_{int} = 0.057), 6844 reflections were observed [I>2σ(I)]. The structure was solved with dual-space methods using SHELXT[6]. Structure refinement was performed with SHELXL-2018[7] on F^2 of all reflections. Non-hydrogen atoms were refined freely with anisotropic displacement parameters. One methyl-indole moiety, the Bpin group and the potassium coordinated 18-crown-6 were refined with disorder models. Hydrogen atoms were introduced in calculated positions and refined with a riding model. 902 Parameters were refined with 1808 restraints (geometry and displacement parameters of disordered groups). R1/wR2 [I > 2σ(I)]: 0.0578 / 0.1433. R1/wR2 [all refl.]: 0.0834 / 0.1577. S = 1.042. Batch scale factor of the second crystal fragment BASF = 0.275(4). Residual electron density between -0.43 and 0.61 e/Å^3. Geometry calculations and checking for higher symmetry was performed with the PLATON program[8].

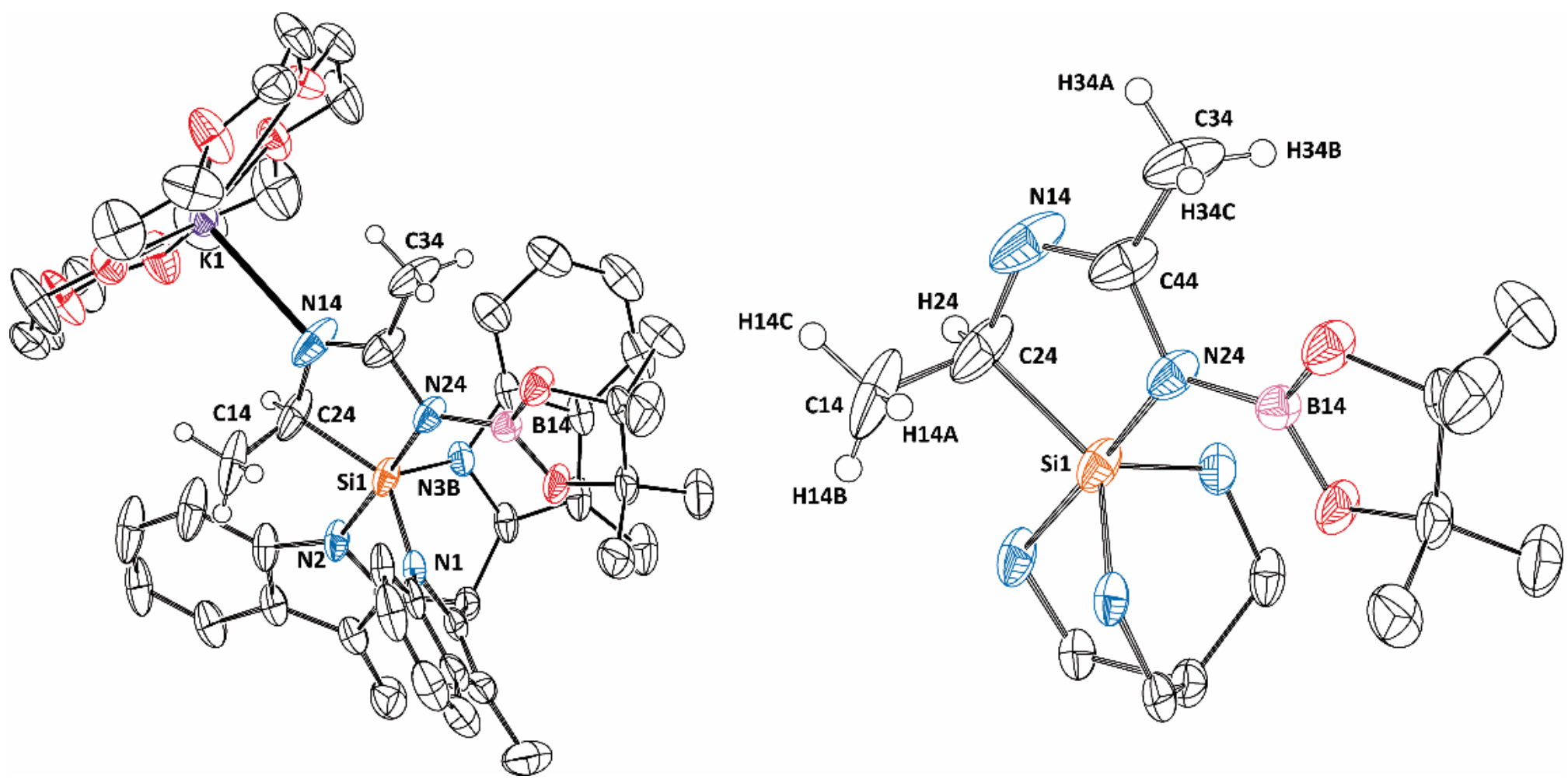

Figure S19. Molecular structure of **7** in the crystal (displacement ellipsoids are drawn at the 50% probability level). C-H hydrogen atoms are omitted for clarity.

4. DFT Studies

4.1. Computational Methods

All calculations were performed using Gaussian 16, Revision C.02[10] without the K+(18-crown-6) cation. Geometry optimizations were carried out in vacuum at the B3LYP-GD3BJ/6-311++G(d,p) level of theory. Frequency analyses on all stationary points were used to ensure that they are minima (no imaginary frequency) or transition states (one imaginary frequency) Transition states were calculated using the synchronous transit-guided quasi-Newton number 3 (QST3) method or using the opt= TS (Berny algorithm) keywords. The guess structure proposed for each transition state calculation was based on the results of relaxed potential energy surface (PES) scans. Single point (SP) calculations were carried at B3LYP-GD3BJ/6-311++G(d,p) with implicit solvation using the SDM model for both THF and MeCN. ΔG° was calculated by taking the energy from the SP at B3LYP-GD3BJ/6-311++G(d,p)/SMD(MeCN or THF) level of theory and adjusting the value with the thermal correction obtained at B3LYP-GD3BJ/6-311++G(d,p) level of theory at 298.15 K and 1 atm. NBO analysis was donde using NBO 7.0 program[11]. NLMOs orbitals and structure of the TS in Figure S_ were visualized using the program Jmol[12].

4.2. Comparison of the Gibbs Energy Using SMD= MeCN or THF

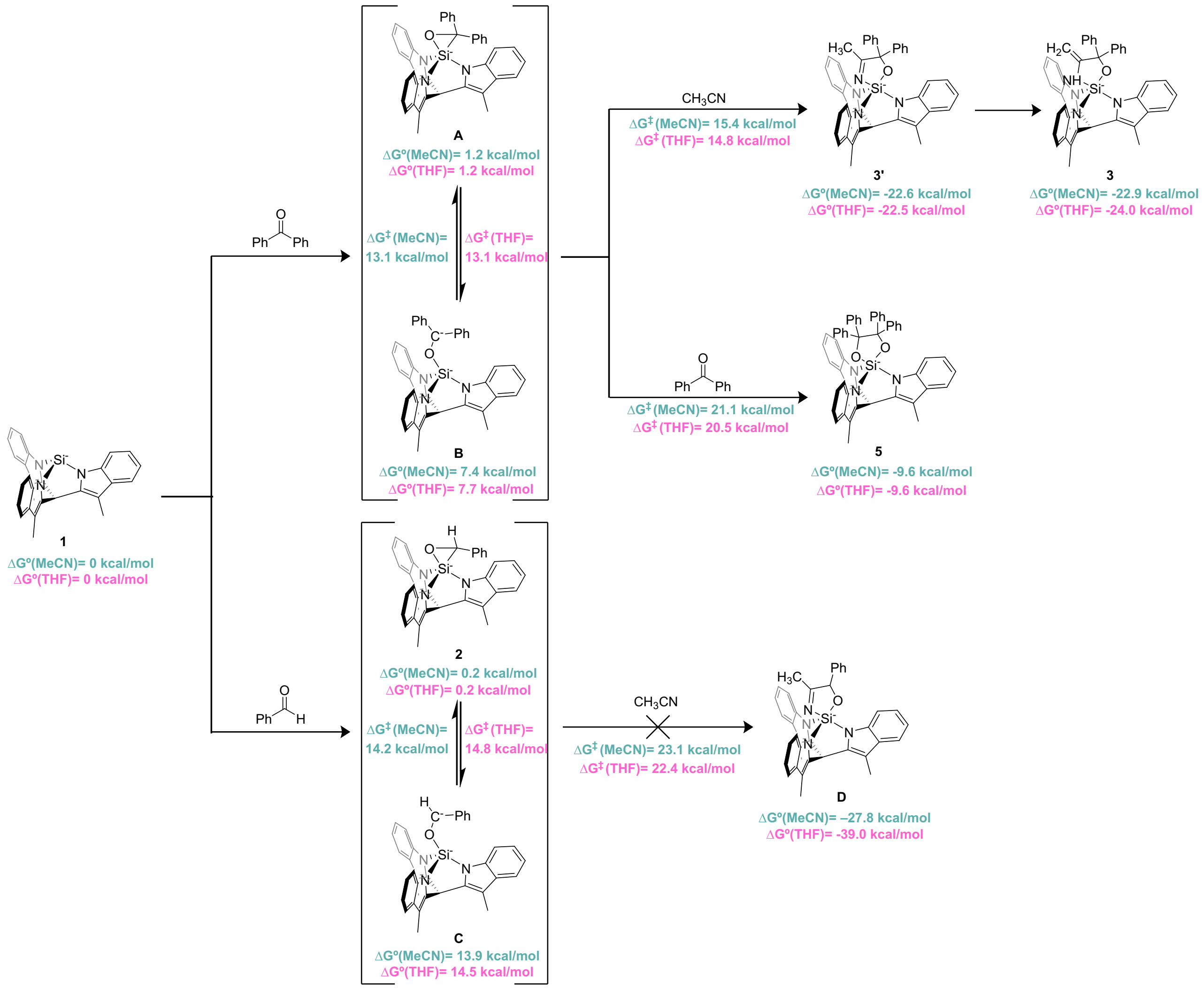

Figure S20. Gibbs energy for the formation of silacyles, calculated at the B3LYP-GD3BJ//6-311++g(d,p)/SMD(Solvent)//B3LYP-GD3BJ//6-311++g(d,p) level of theory. Solvent = MeCN (in blue) and THF (in violet).

To assess the effect of solvent on geometry optimization, selected ground-state structures and transition states were re-optimized with the implicit solvent model for acetonitrile (the most polar of the considered solvents). The results, summarized in the table below, show only very minor deviations (< 1kcal/mol) that would not impact any of the conclusions of the study.

Compound	ΔGº respect to compound 1	
	ΔGº at b3lyp-D3BJ/6-311++g(d,p)/SMD(MeCN)// b3lyp-D3BJ/6-311++g(d,p) level of theory	ΔGº at b3lyp-D3BJ/6-311++g(d,p)/SMD(MeCN) level of theory
1	0	0
A	1.2	1.2
B	7.4	7.2
TS A to B	13.4	13.1
2	0.2	-0.2
C	13.9	13.6
TS 2 to C	14.2	14.0
3’	-22.6	-22.0
TS B to 3’	15.4	14.5
D	-27.8	-27.8

4.3. Calculations on the Formation of MeCN Silacycle (6)

Calculations on the formation of silacycle **6** started computing the interaction of silanide **1** with MeCN. η^1 type of coordination showed to be energetically prohibited at 29.8 kcal/mol using SMD=MeCN as solvent and 30.0 kcal/mol using SMD=THF; for η^2 type coordination, intermediate E is located at 20.3 kcal/mol using SMD=MeCN as solvent and 19.8 kcal/mol using SMD=THF. These energy values are remarkably different than the values obtained from the coordination of benzophenone and benzaldehyde. Nonetheless, we explored three different mechanisms that could lead to silacycle **6** as presented in Figure S21. A one-step concerted mechanism, without forming an acetonitrile adduct intermediate, resulted not to be feasible at 46.1 kcal/mol using SMD=MeCN as solvent and 44.8 kcal/mol using SMD=THF. From intermediates **E** and **F** a one-step transition state resembling the one found for benzophenone was found to be prohibitively high in energy at 57.7 kcal/mol using SMD=MeCN as solvent and 56.5 kcal/mol using SMD=THF. A comparison of both one-step transition states towards **6** is depicted in Figure S21. A stepwise mechanism was contemplated through an open-cycle intermediate (**6-open**), however this pathway also resulted to be too high energetically for the reaction conditions at 47.9 kcal/mol using SMD=MeCN as solvent and 48.3 kcal/mol using SMD=THF. Other possible intermediates were considered (Figure S23), for instance the interaction of two silanide molecules with acetonitrile (**G**) or pathways where one of the indole arms is detached from the silicon atom, ultimately all these intermediates resulted to present a high energy that did not match the current reaction conditions for the formation of **6**.

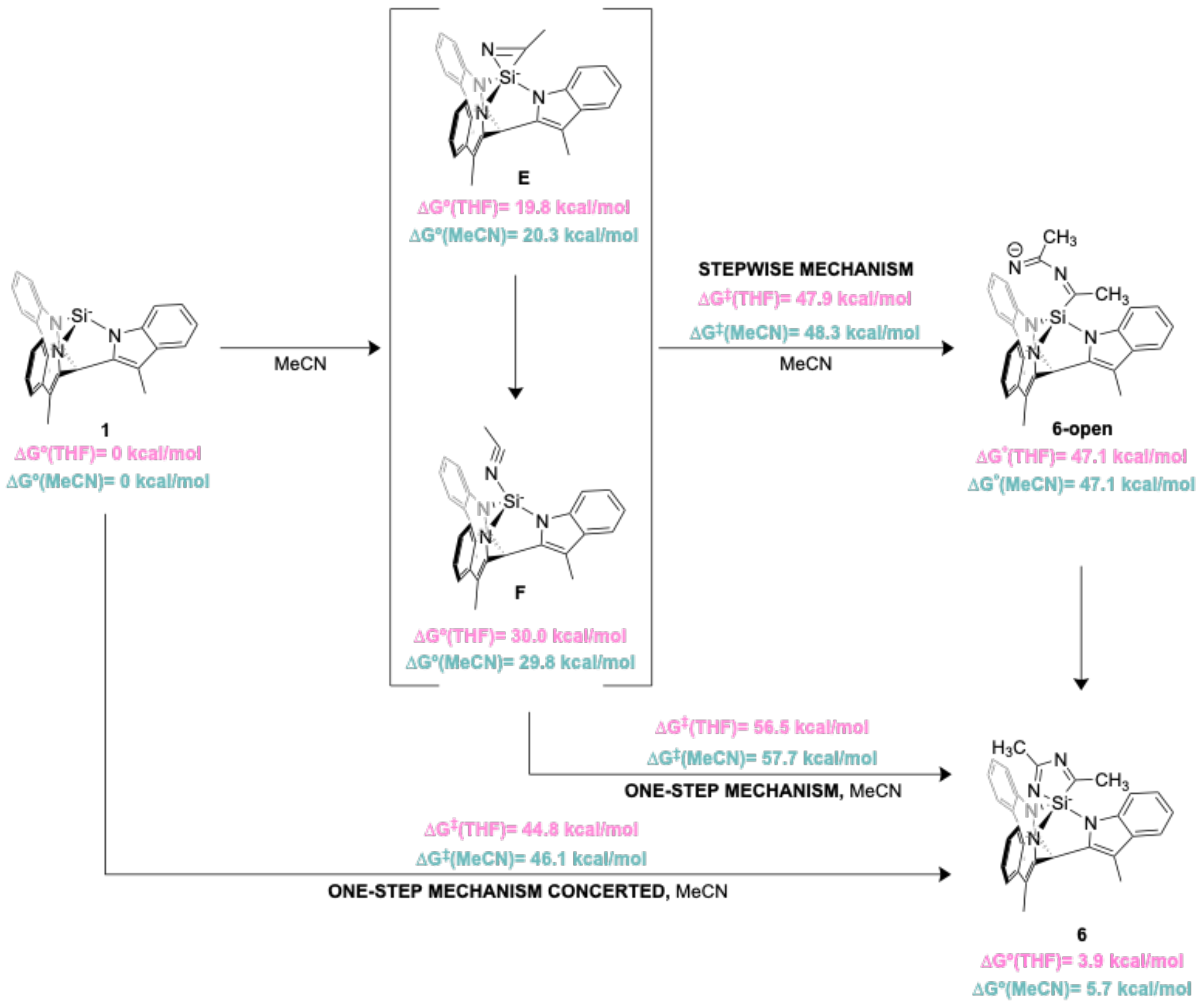

Figure S21. Gibbs energy for the formation of silacyles, calculated at the B3LYP-GD3BJ//6-311++g(d,p)/ SMD(Solvent)//B3LYP-GD3BJ//6-311++g(d,p) level of theory. Solvent= MeCN (in blue) and THF (in violet).

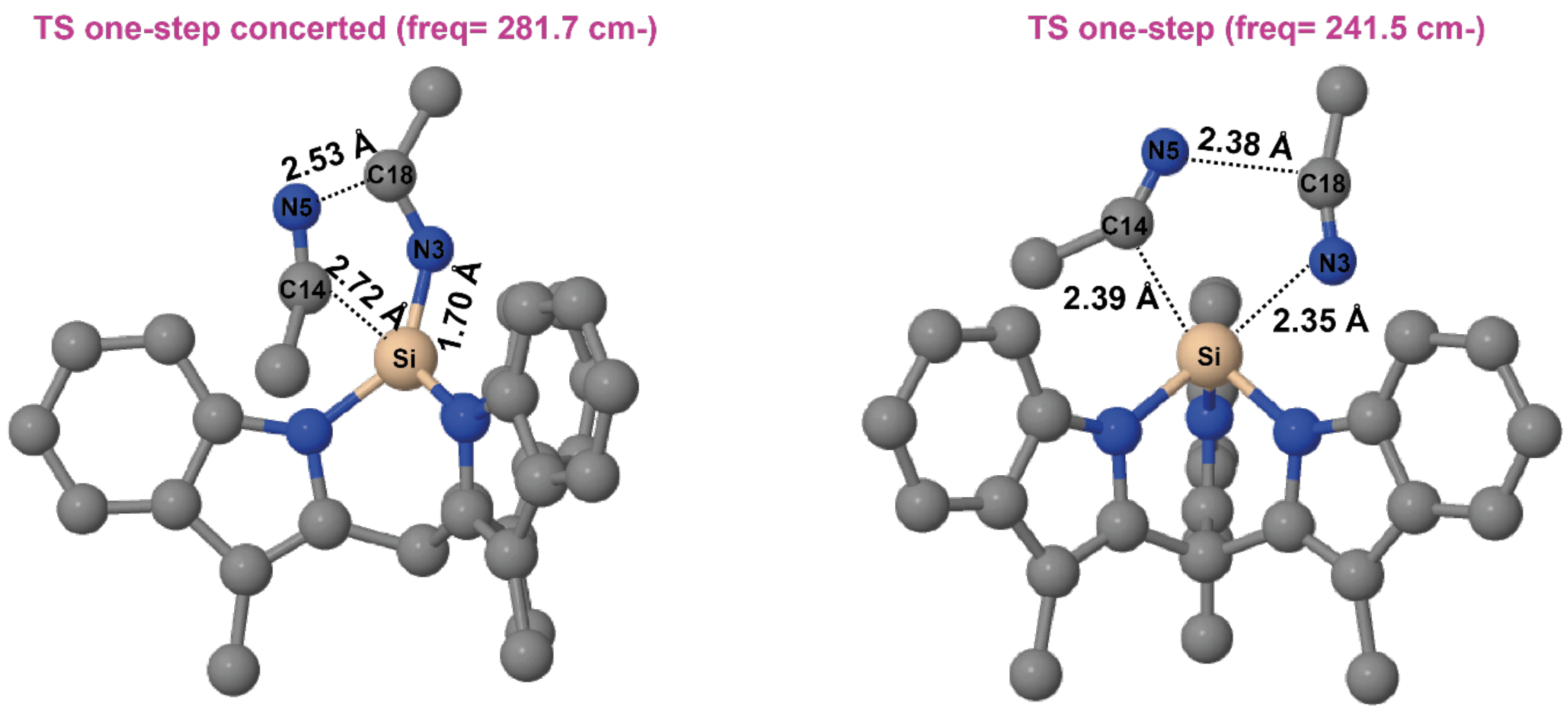

Figure S22. Comparison of the transition states (TS) in one-step for the synthesis of silacycle **6** optimized at the B3LYP-GD3BJ//6-311++g(d,p) level of theory.

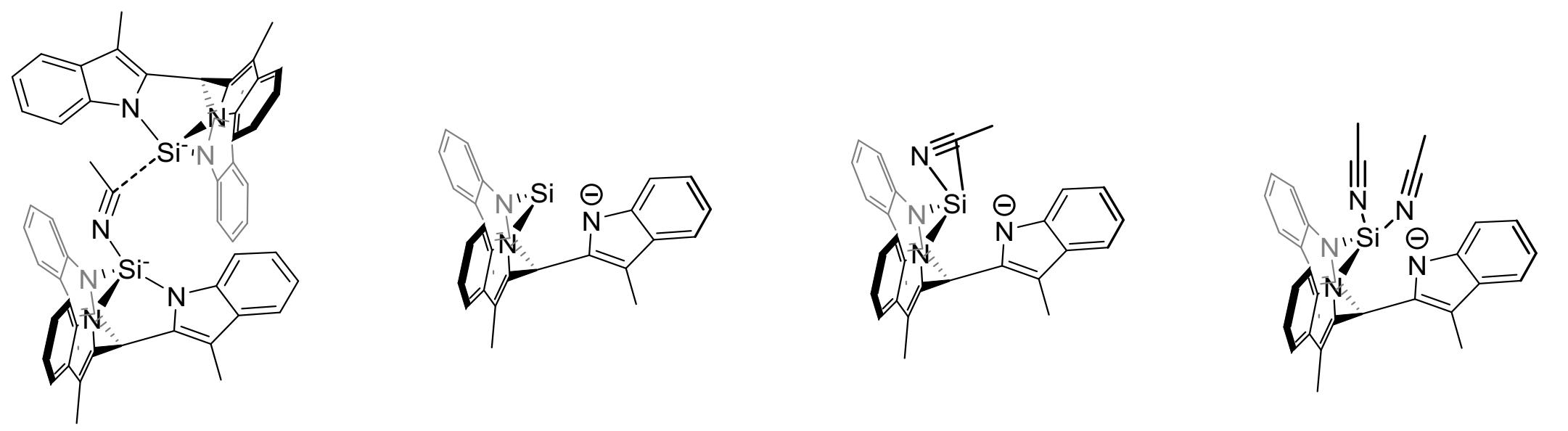

G	1-noNarm	1-noNarm-1MeCN	1-noNarm-2MeCN
ΔGº(THF)= 29.6 kcal/mol	ΔGº(THF)= 36.7 kcal/mol	ΔGº(THF)= 46.1 kcal/mol	ΔGº(THF)= 73.6 kcal/mol
ΔGº(MeCN)= 24.7 kcal/mol	ΔGº(MeCN)= 35.8 kcal/mol	ΔGº(MeCN)= 46.1 kcal/mol	ΔGº(MeCN)= 73.4 kcal/mol

Figure S23. Other proposed intermediates for the formation of silacycle **7.** Gibbs energy was calculated at the B3LYP-GD3BJ//6-311++g(d,p)/SMD(Solvent)//B3LYP-GD3BJ//6-311++g(d,p) level of theory. Solvent= MeCN (in blue) and THF (in violet).

4.4. Additional Calculations on the Formation of the Silacycles Derived from Benzophenone

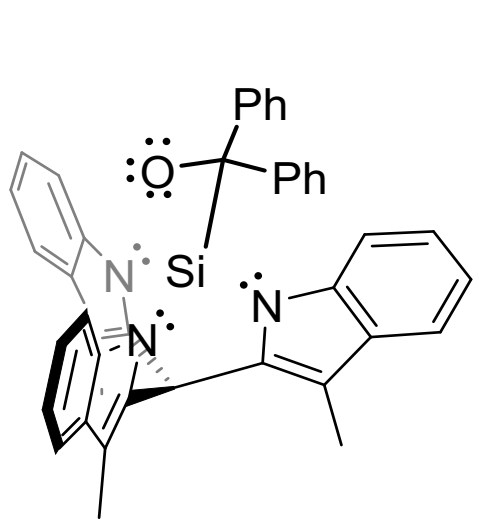

A

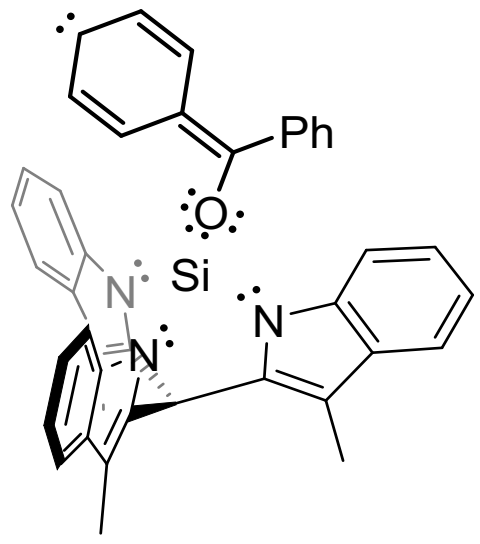

B

Figure S24. Lewis structures of intermediates **A** and **B** from the NBO calculation calculated at the B3LYP-GD3BJ//6-311++g(d,p)//B3LYP-GD3BJ//6-311++g(d,p) level of theory.

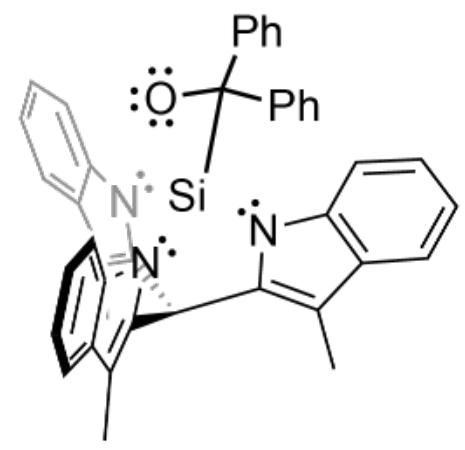

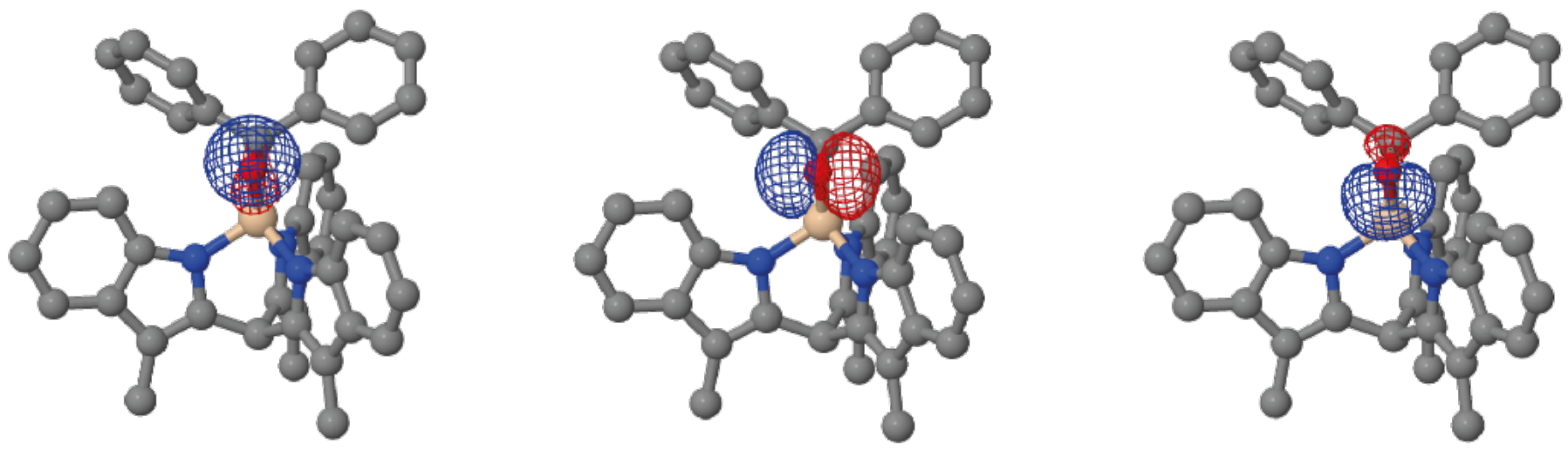

LP (1) O2
98.7% O2

LP (2) O2
3.3 % Si
93.3% O2

LP (3) O2
11.6 % Si
86.7% O2

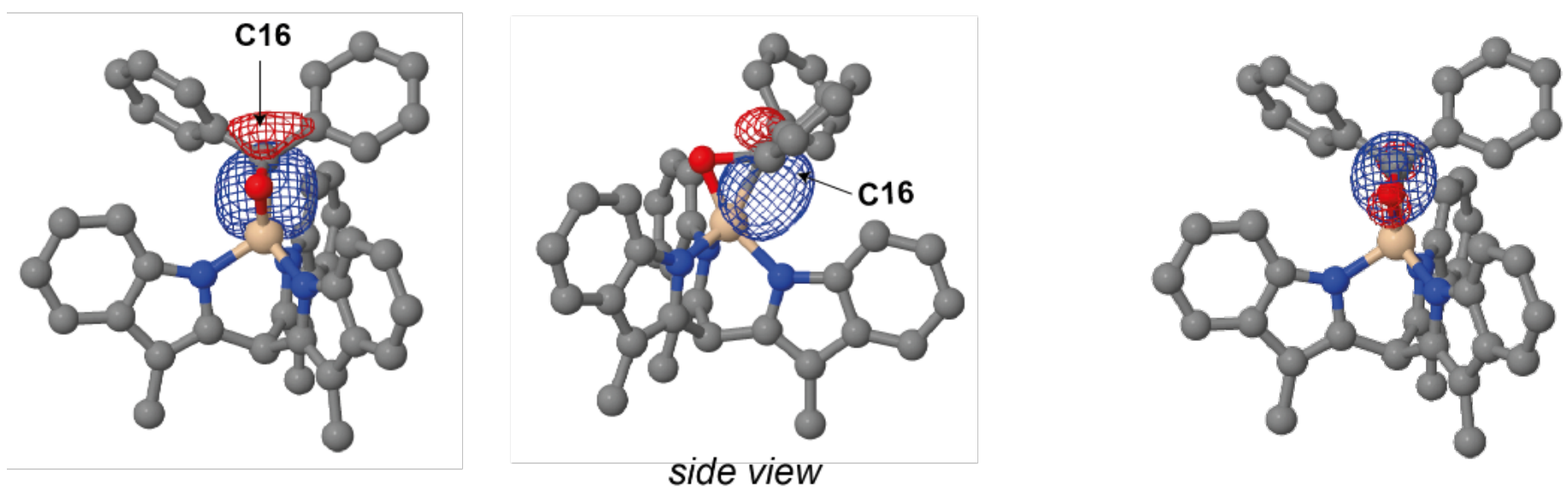

BD (1) Si1 - C16
22.4% Si1
73.0% C16

BD (1) O2 - C16
64.1% O2
34.0% C16

Figure S25. Representative NLMOs of intermediate **A** from the NBO calculation calculated at the B3LYP-GD3BJ//6-311++g(d,p)//B3LYP-GD3BJ//6-311++g(d,p) level of theory.

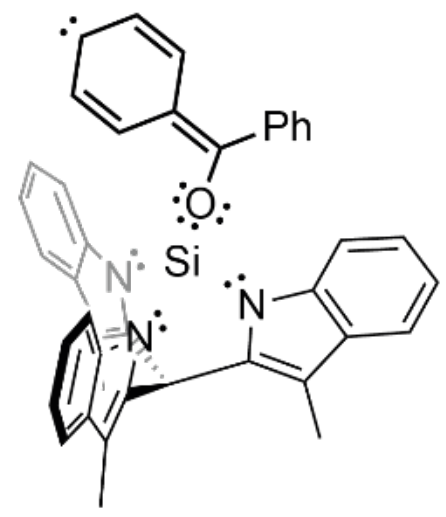

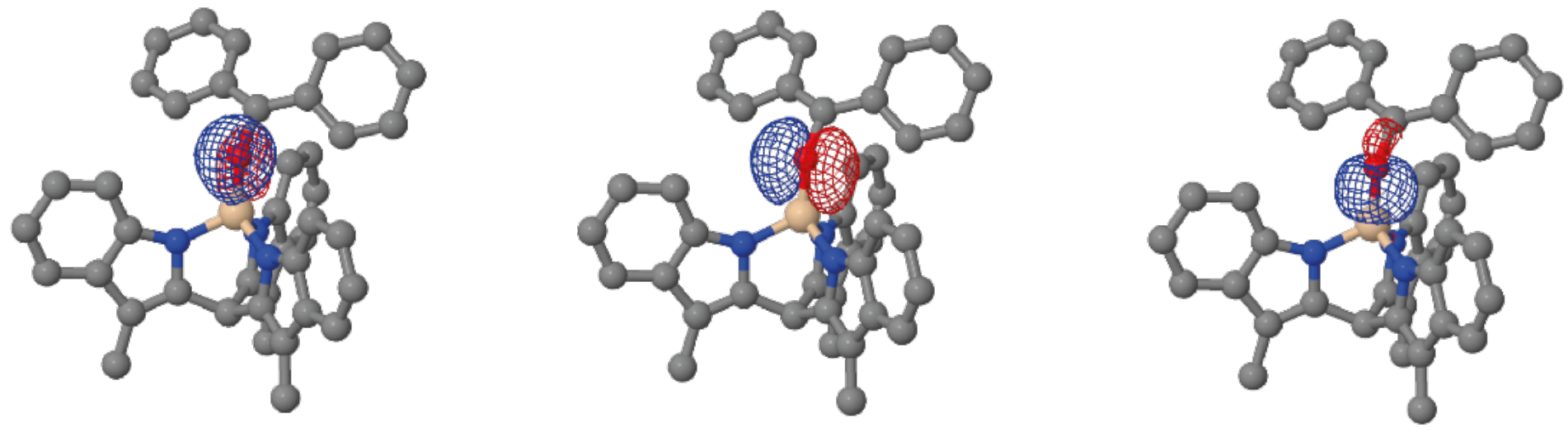

LP (1) O2
3.2 % Si
95.1% O2

LP (2) O2
3.7 % Si
94.4% O2

LP (3) O2
14.6 % Si
83.7% O2

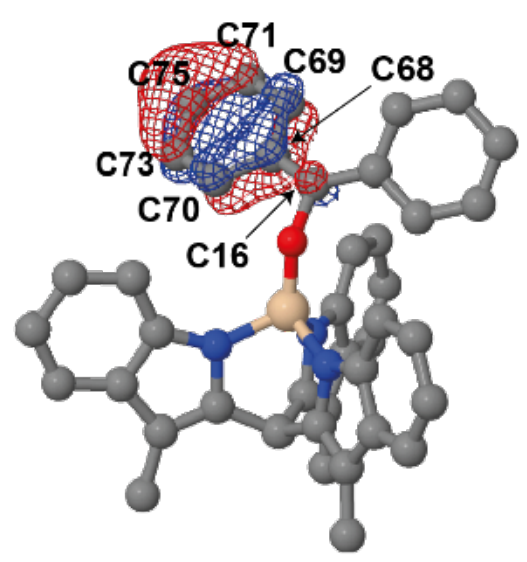

LP (1) C75
3.0 % C16
11.3% C68
7.0% C69
7.3 C70
8.9% C71
9.6% C73
49.0% C75

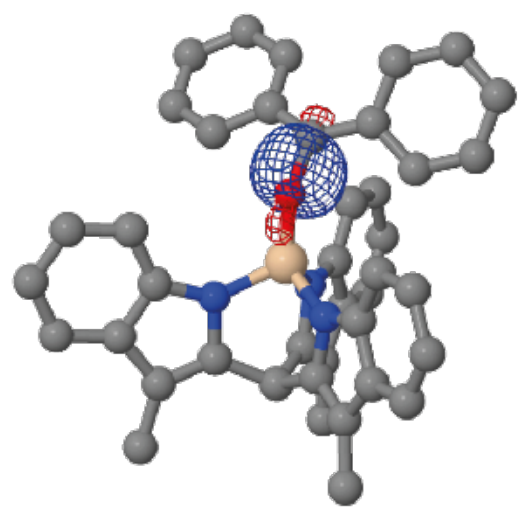

BD (1) O2 - C16
71.7% O2
27.5% C16

Figure S26. Representative NLMOs of intermediate **B** from the NBO calculation calculated at the B3LYP-GD3BJ//6-311++g(d,p)//B3LYP-GD3BJ//6-311++g(d,p) level of theory.

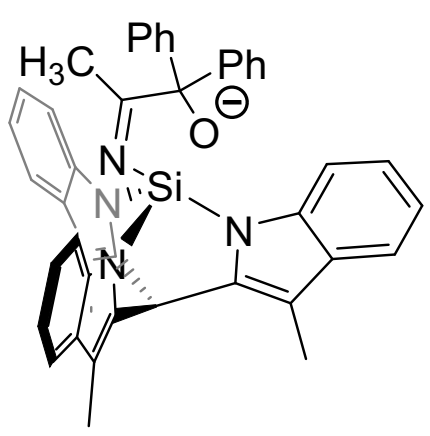

3'-openO
ΔGº(THF)= 13.2 kcal/mol
ΔGº(MeCN)= 13.3 kcal/mol

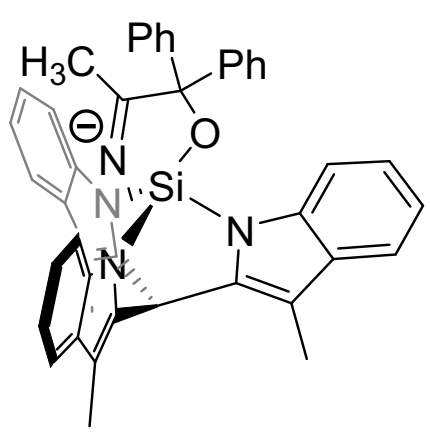

3'-openN
ΔGº(THF)= 27.1 kcal/mol
ΔGº(MeCN)= 25.7 kcal/mol

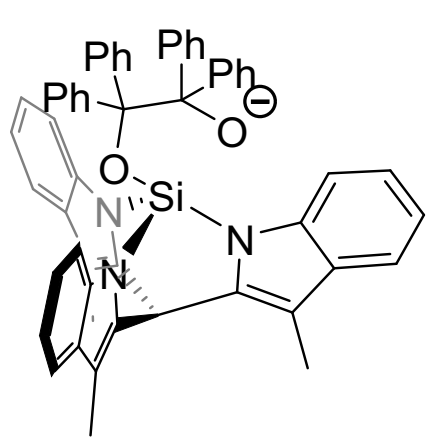

5-open
ΔGº(THF)= 22.0 kcal/mol
ΔGº(MeCN)= 21.3 kcal/mol

Figure S27. Other proposed intermediates for the formation of silacycle **3'** and **5**. Gibbs energy was calculated at the B3LYP-GD3BJ//6-311++g(d,p)/SMD(Solvent)//B3LYP-GD3BJ//6-311++g(d,p) level of theory. Solvent= MeCN (in blue) and THF (in violet).

4.5. Table of Energies

Compound	ΔGº energy from SP calculated at B3LYP-GD3BJ/ 6-311++G(d,p)/SMD(MeCN)	ΔGº energy from SP calculated at B3LYP-GD3BJ/ 6-311++G(d,p)/SMD(THF)	Thermal correction calculated at B3LYP-GD3BJ/6-311++G(d,p)
**Benzophenone**	-576.851897969	-576.850566603	0.153186
**MeCN**	-132.811810839	-132.809957529	0.022175
**Benzaldehyde**	-345.706595259	-345.705686283	0.078852
**1**	-1534.70905895	-1534.69940360	0.369714
**2**	-1880.439977	-1880.429358	0.473246
**3**	-2244.46088192	-2244.44987007	0.596722
**3’**	-2244.45882575	-2244.44576500	0.595042
**5**	-2688.48608454	-2688.47374362	0.733937
**6**	-1800.36960772	-1800.35908666	0.460017
**A**	-2111.58492646	-2111.57394240	0.548748
**B**	-2111.57195813	-2111.56057788	0.545735
**C**	-1880.41379121	-1880.40228105	0.468946
**D**	-2013.32098254	-2013.30819964	0.519934
**1-noNarm**	-1534.64791917	-1534.63690020	0.365668
**1-noNarm-1MeCN**	-1667.46508733	-1667.45347634	0.409528
**1-noNarm-2MeCN**	-1800.24680082	-1800.23314040	0.445180
**E**	-1667.49150128	-1667.47962659	0.409994
**F**	-1667.50975413	-1667.49907968	0.413120
**G**	-3202.23768458	-3202.20853991	0.808638
**6-open**	-1800.29894789	-1800.28556686	0.455344
**3’-openN**	-2244.37697632	-2244.36192329	0.590302
**3’-openO**	-2244.39813173	-2244.38540972	0.591652
**5-open**	-2688.43348627	-2688.42010707	0.730669
**TS A to B**	-2111.56448028	-2111.55354345	0.547342
**TS B to 3’**	-2244.39537641	-2244.38352477	0.592285
**TS 2 to C**	-1880.413687	-1880.402214	0.469315
**TS B to 5**	-2688.43182923	-2688.42045497	0.728691
**TS C to D**	-2013.23595271	-2013.22449139	0.515965
**TS E to 6 concerted**	-1800.29913584	-1800.28777591	0.453999
**TS E to 6**	-1800.27916576	-1800.26776006	0.452544
**TS E to 6-open**	-1800.29519039	-1800.28236178	0.453523

5. Spectra of the Isolated Compounds

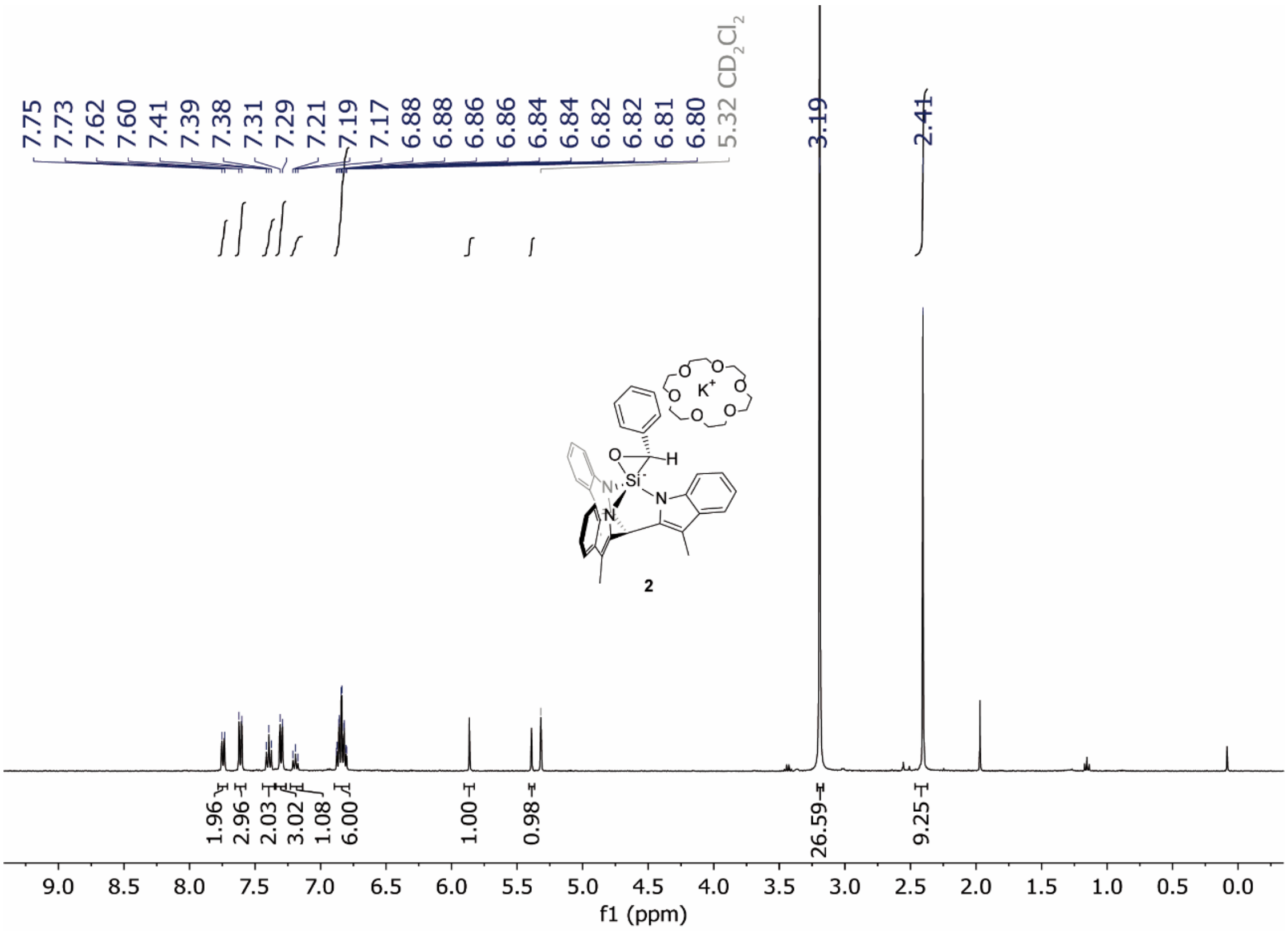

Figure S28. ^{1}H-NMR (400 MHz, CD_2Cl_2, 25°C) spectrum of compound **2**: δ 7.74 (d, *J* = 8.0 Hz, 2H, CPhHO-H_{Ar}), 7.61 (d, *J* = 8.9 Hz, 3H, Indole-*H*), 7.39 (t, *J* = 7.7 Hz, 2H, CPhHO-H_{Ar}), 7.30 (d, *J* = 7.6 Hz, 3H, Indole-*H*), 7.19 (t, *J* = 7.4 Hz, 1H, CPhHO-H_{Ar}), 6.84 (m, *J* = 7.0, 1.5 Hz, 6H, Indole-*H*), 5.86 (s, 1H, Indole-C*H* linker), 5.39 (s, 1H, CPh*H*O), 3.19 (s, 24H, 18-crown-6), 2.41 (s, 9H, Indole-CH_3). Impurities: 1.12 and 3.42 ppm – Et_2O, 1.96 ppm– CH_3CN.

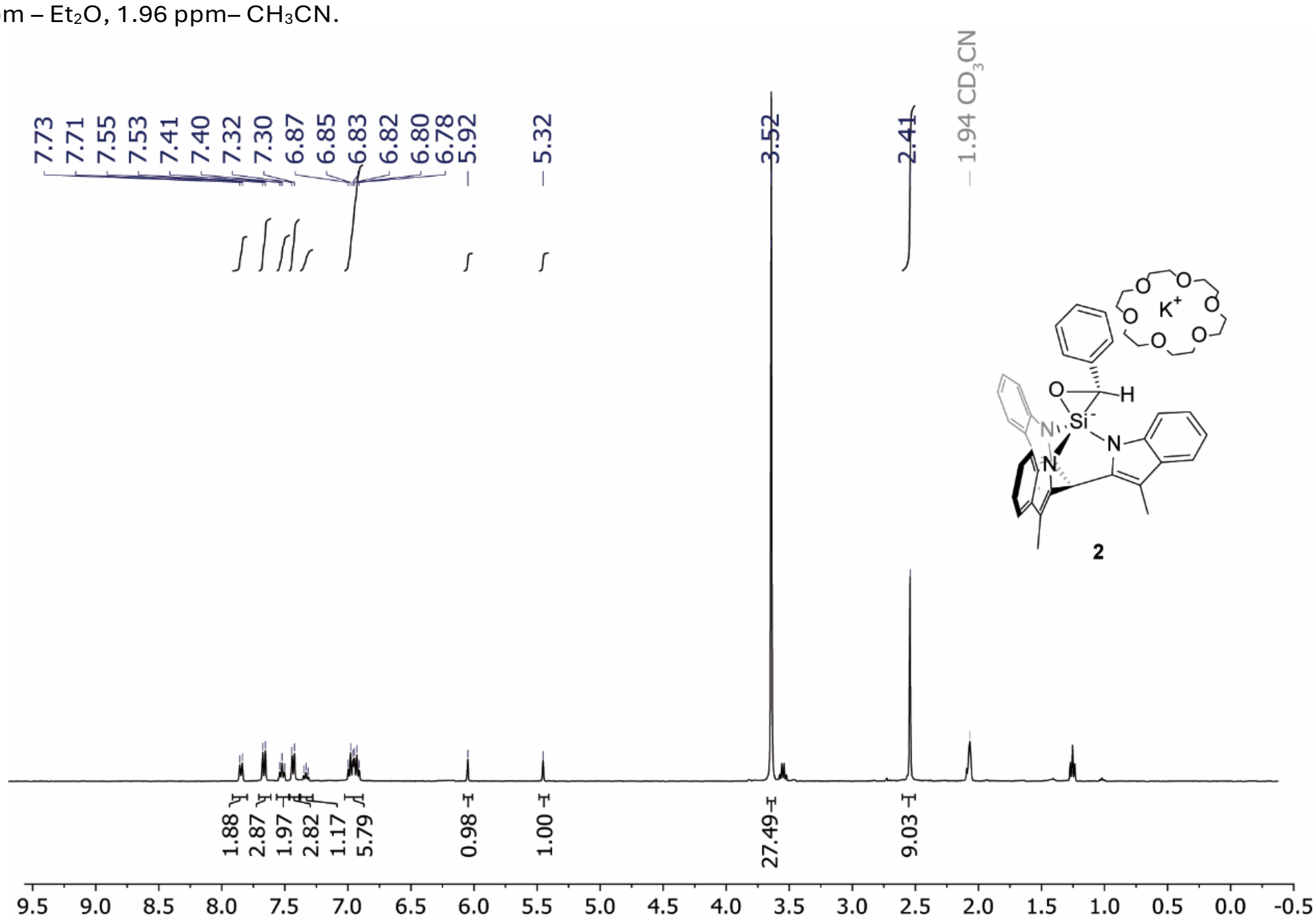

Figure S29. ^{1}H-NMR (400 MHz, CD_3CN, 25°C) spectrum of compound **2**: δ 7.72 (d, *J* = 8.0 Hz, 2H, CPhHO-H_{Ar}), 7.54 (d, *J* = 8.1 Hz, 3H, Indole-*H*), 7.39 (t, *J* = 7.9 Hz, 2H, CPhHO-H_{Ar}), 7.31 (d, *J* = 8.1 Hz, 3H, Indole-*H*), 7.20 (t, *J* = 7.4 Hz, 1H, CPhHO-H_{Ar}), 6.83 (m, *J* = 20.0, 7.1 Hz, 6H, Indole-*H*), 5.92 (s, 1H, Indole-C*H* linker), 5.32 (s, 1H, CPh*H*O), 3.52 (s, 24H,18-crown-6), 2.41 (s, 9H, Indole-CH_3).

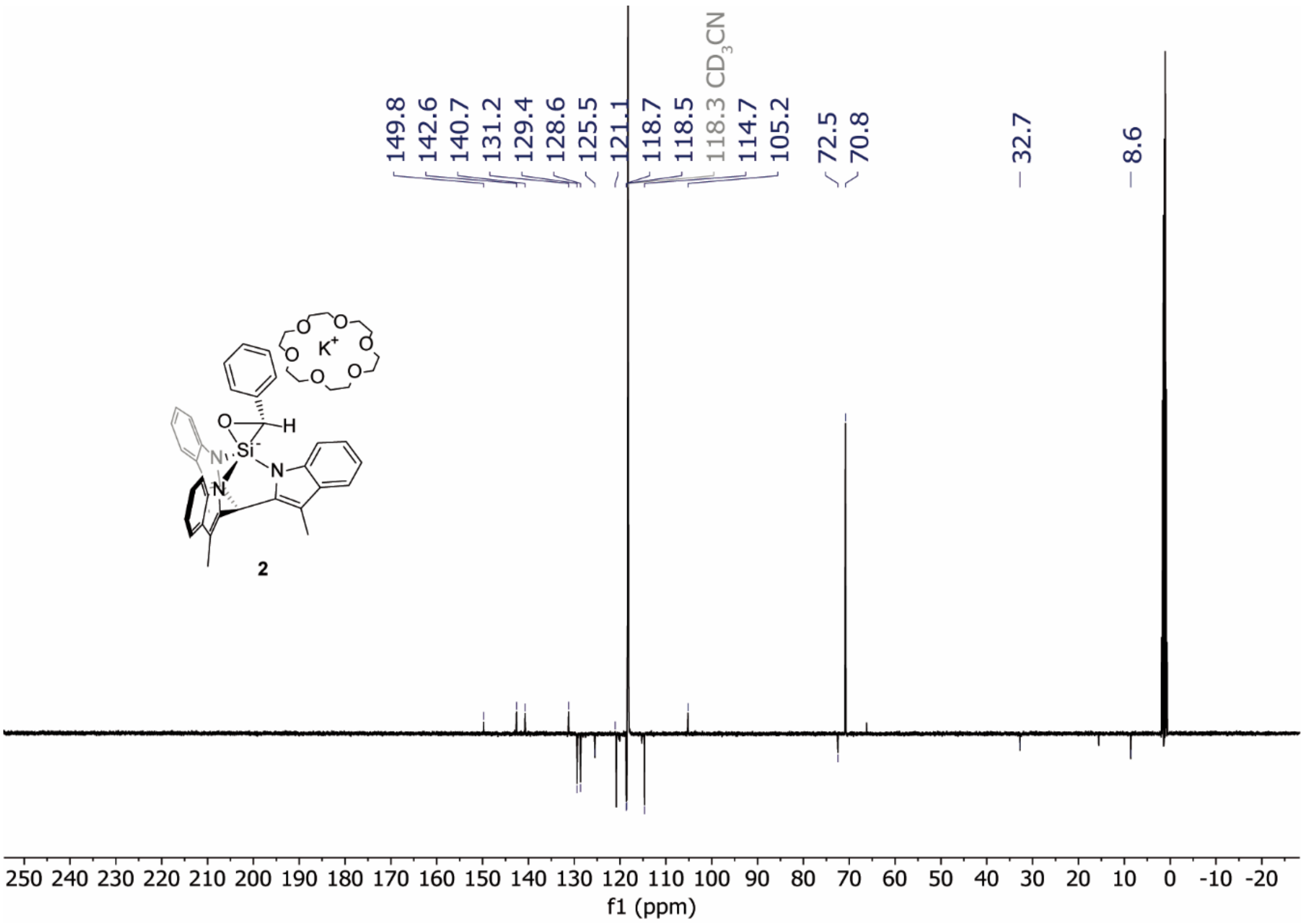

Figure S30. ^{13}C-APT-NMR (APT, 101 MHz, CD_3CN, 25°C) spectrum of compound **2**: δ 149.8 (s, CPhHO-C_{Ar}), 142.6 (s, C_{Indole}), 140.7(s, C_{Indole}), 131.2 (s, C_{Indole}), 129.4 (s, CPhHO-CH_{Ar}), 128.6(s, CPhHO-CH_{Ar}), 125.5 (s, CPhHO-CH_{Ar}), 121.1 (s, CH_{Indole}), 118.7 (s, CH_{Indole}), 118.5 (s, CH_{Indole}), 114.7 (s, CH_{Indole}), 105.2 (s, C_{Indole}), 72.5 (s, CPhHO), 70.8 (s, CH_2,18-crown-6), 32.7 (s, CH_{Indole} linker), 8.6 (s, $CH_{3\text{-}Indole}$).

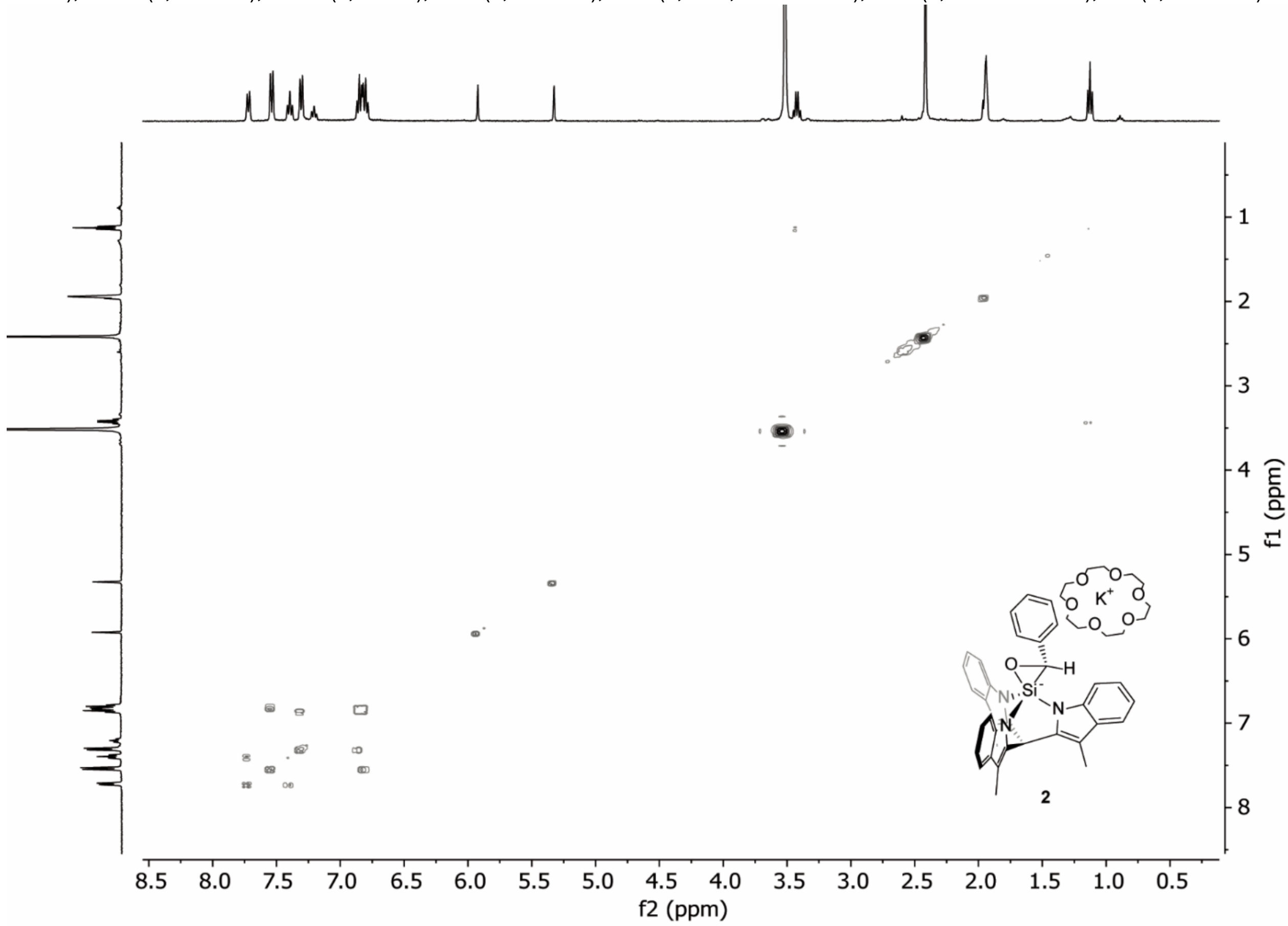

Figure S31. ^{1}H-COSY (400 MHz, CD_3CN, 25°C) spectrum of compound **2**.

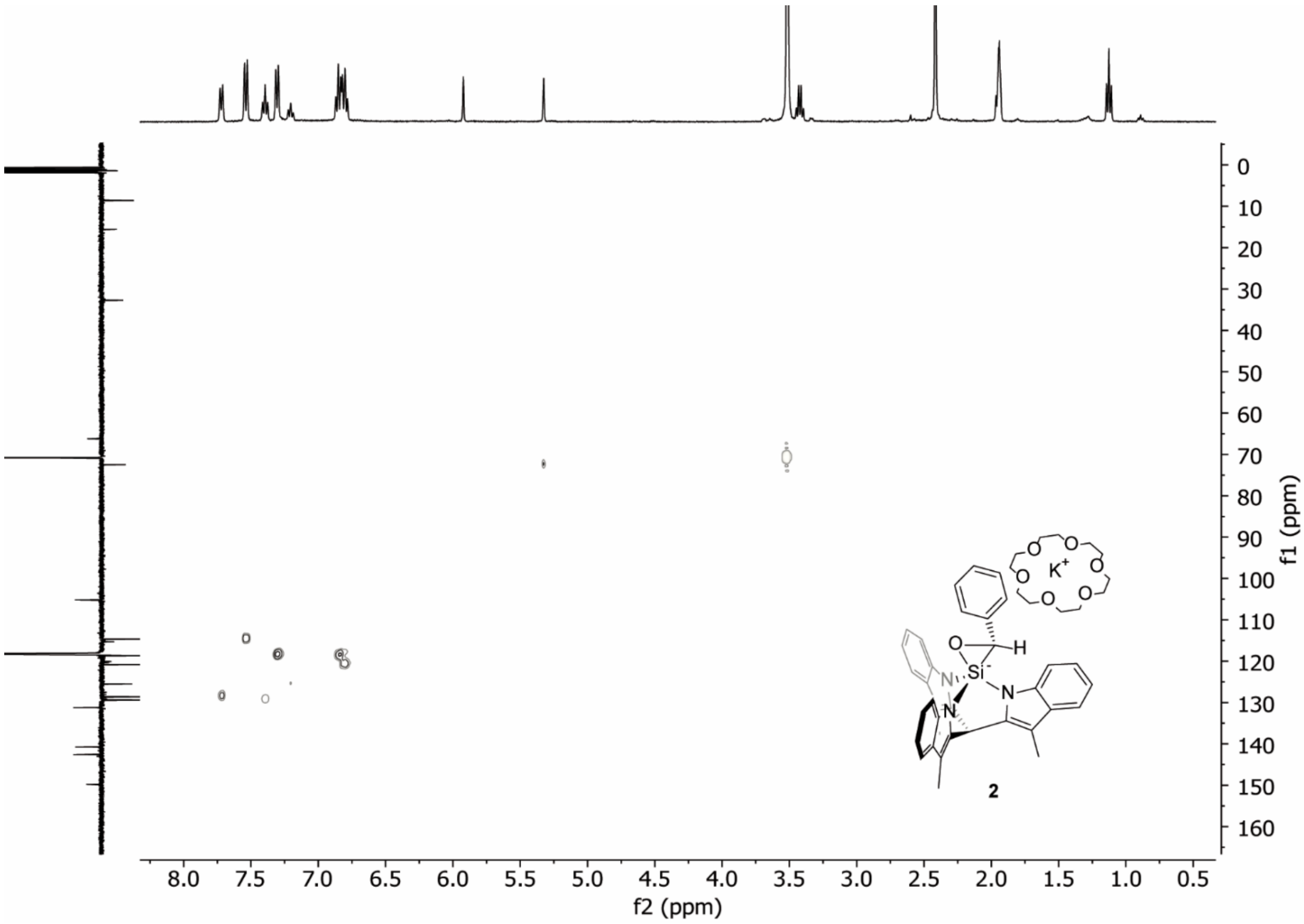

Figure S32. ^{1}H-^{13}C HSQC (400 MHz/100 MHz, CD_3CN, 25°C) spectrum of compound **2**.

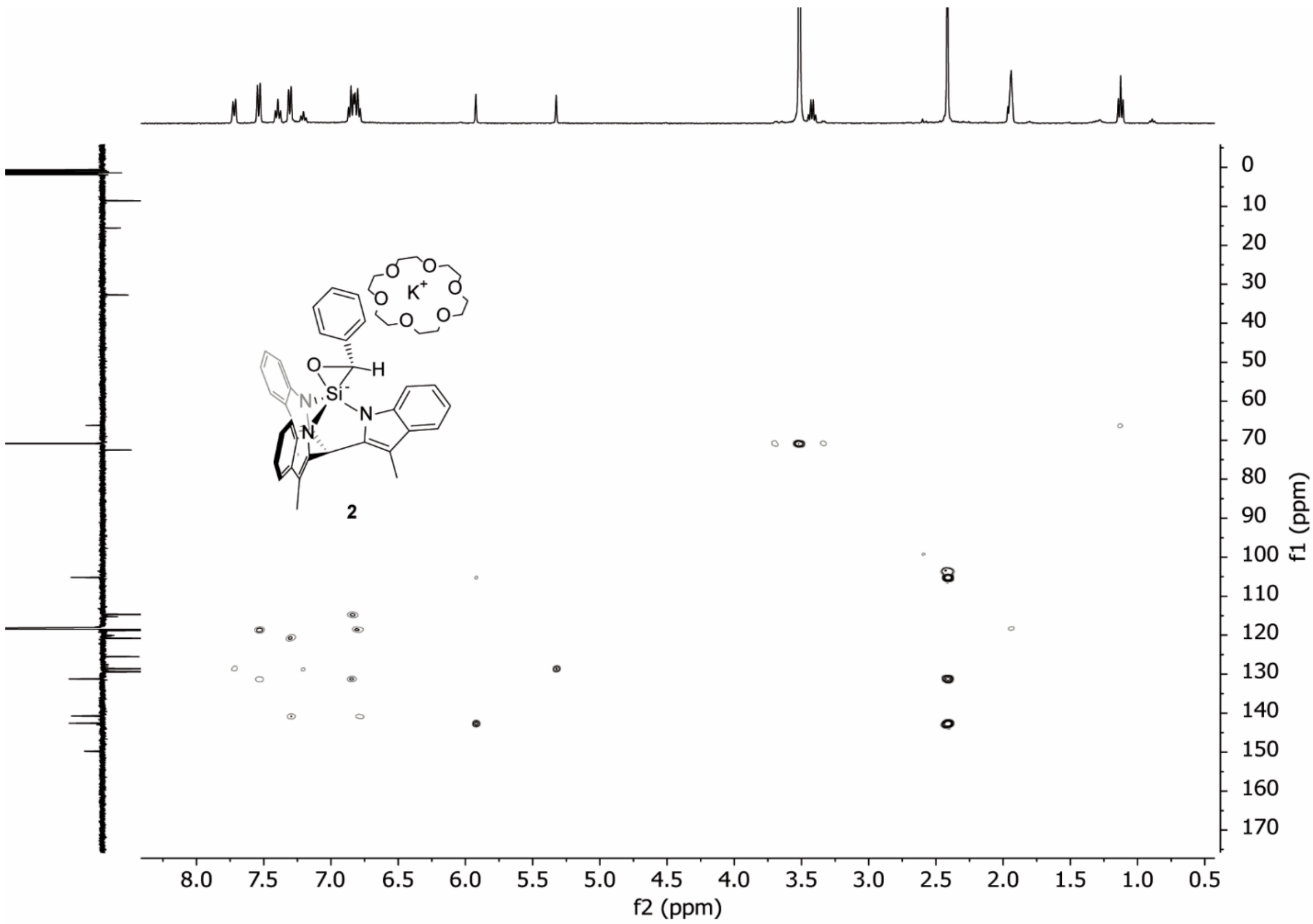

Figure S33. ^{1}H-^{13}C HMBC (400 MHz/100 MHz, CD_3CN, 25°C) spectrum of compound **2**.

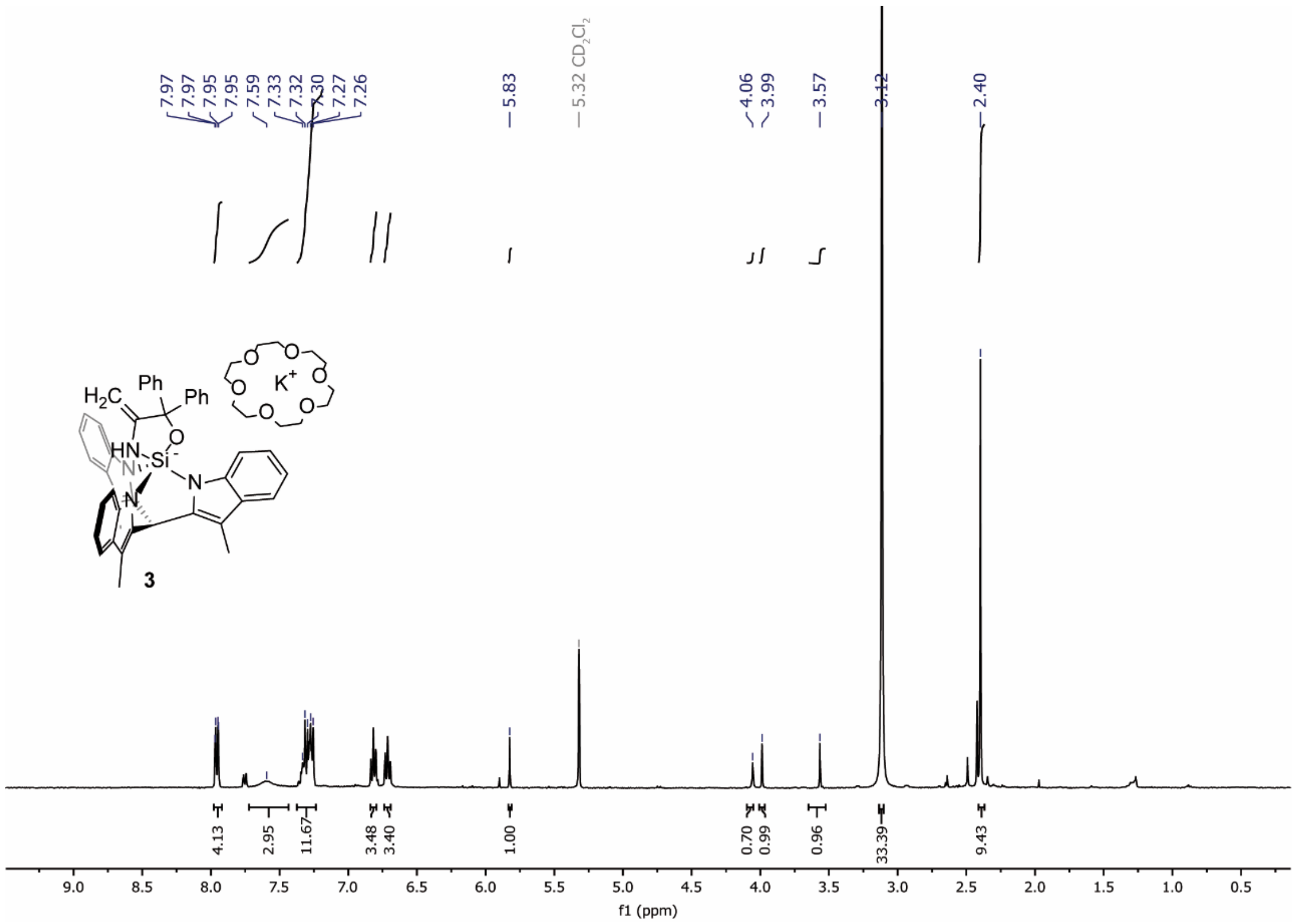

Figure S34. ^{1}H-NMR (400 MHz, CD_2Cl_2, 25°C) spectrum of compound **3**:. δ 7.98 – 7.92 (m, 4H, Ph_2CO-H_{Ar}), 7.59 (s, 3H, Indole-*H*), 7.37 – 7.24 (m, 12H, Indole-*H* and Ph_2CO-H_{Ar}), 6.84 – 6.80 (m, 3H, Indole-*H*), 6.71 (ddd, *J* = 8.3, 6.9, 1.4 Hz, 3H, Indole-*H*), 5.83 (s, 1H C*H* linker), 4.06 (s, 1H, N*H*CCH$_2$), 3.99 (s, 1H, NHCC*H*$_2$), 3.57 (s, 1H, NHCC*H*$_2$), 3.12 (s, 24H, 18-Crown-6), 2.40 (s, 9H, Indole-C*H*$_3$).

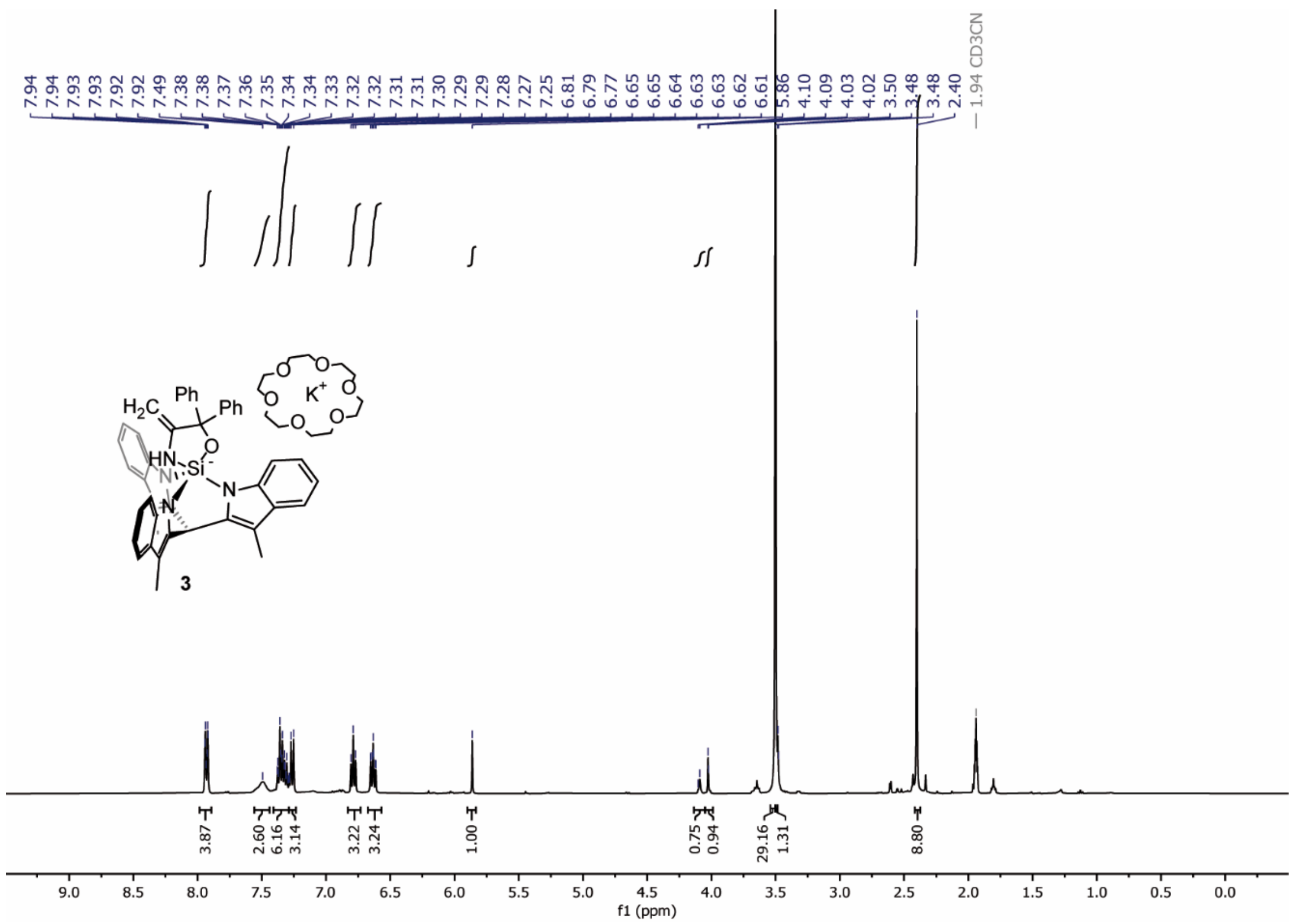

Figure S35. ^{1}H-NMR (400 MHz, CD_3CN, 25°C) spectrum of compound **3**: δ 7.93 (dt, *J* = 6.3, 1.5 Hz, 4H, Ph_2CO-H_{Ar}), 7.49 (br, 3H, Indole-*H*), 7.41 – 7.29 (m, 6H, Ph_2CO-H_{Ar}), 7.26 (d, *J* = 7.8 Hz, 3H, Indole-*H*), 6.79 (t, *J* = 7.3 Hz, 3H, Indole-*H*), 6.63 (ddd, *J* = 8.3, 6.9, 1.3 Hz, 3H, Indole-*H*), 5.86 (s, 1H, Indole-C*H* linker), 4.10 (d, *J* = 5.5 Hz, 1H, NHCC*H*$_2$), 4.02 (d, *J* = 1.7 Hz, 1H, N*H*CCH$_2$), 3.50 (s, 24H, 18-Crown-6), 3.48 (d, J = 1.8 Hz, 1H, NHCC*H*$_2$), 2.40 (s, 9H, Indole-C*H*$_3$).

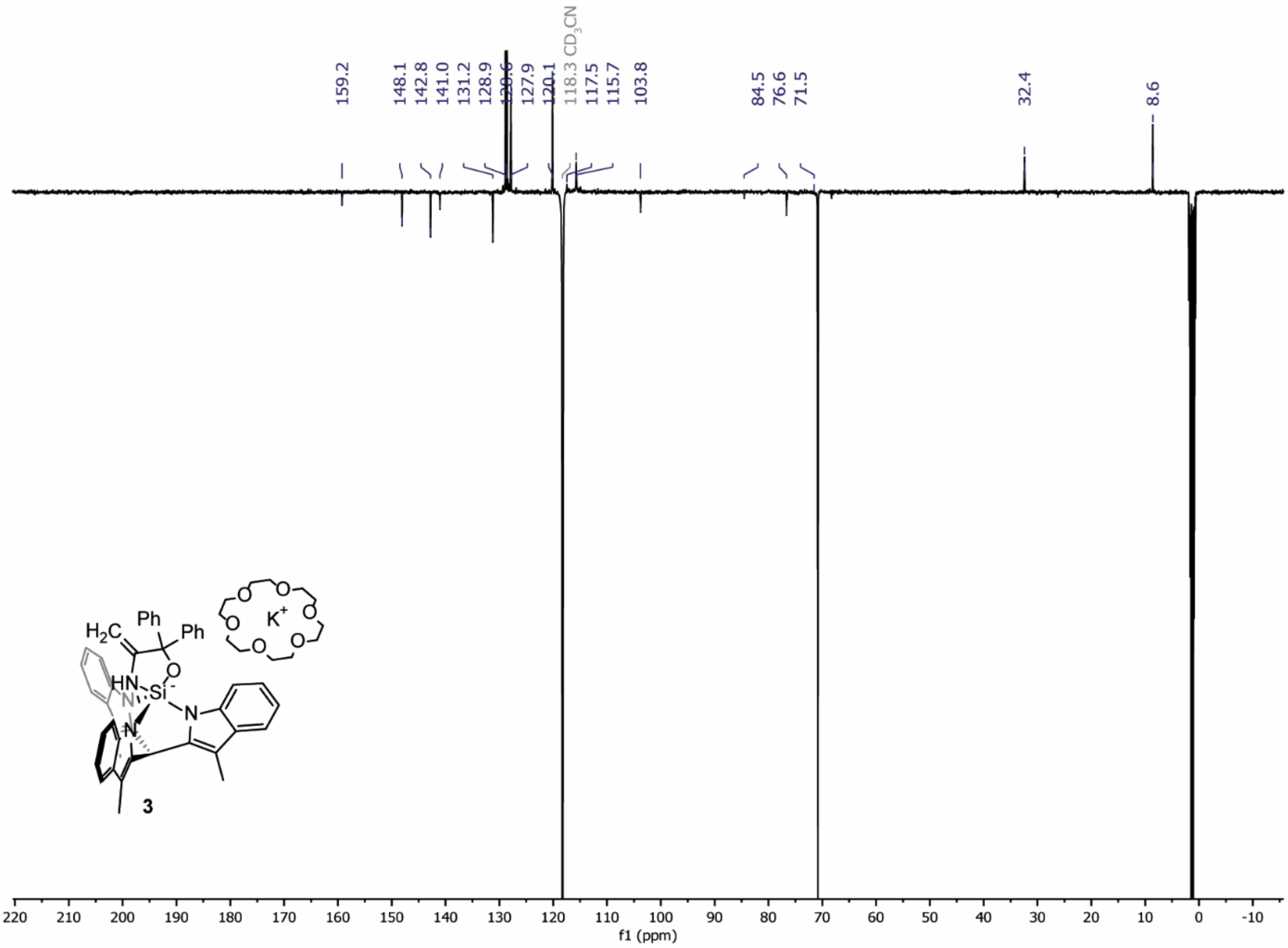

Figure S36. ^{13}C-APT- NMR (101 MHz, CD_3CN, 25°C) spectrum of compound **3**: δ 159.2 (s, Ph*C*N), 148.1 (s, NH*C*=CH_2), 142.8 (s, C_{Indole}), 141.0 (s, C_{Indole}), 131.2 (s, C_{Indole}), 128.9 (s, Ph_2CO-CH_{Ar}), 128.6(s, Ph_2CO-CH_{Ar}), 127.9(s, NH*C*CH_2), 120.1(s, CH_{Indole}), 117.5(s, CH_{Indole}), 115.7(s, CH_{Indole}), 103.8(s, C_{Indole}), 84.5 (s, Ph_2*C*O), 76.6(s, NHC=CH_2), 71.5 (s, CH_2,18-crown-6), 32.4 (s, CH_{Indole} linker), 8.6(s, $CH_{3\text{-}Indole}$). (CH_{Indole} around 118.3 ppm covered by solvent signal).

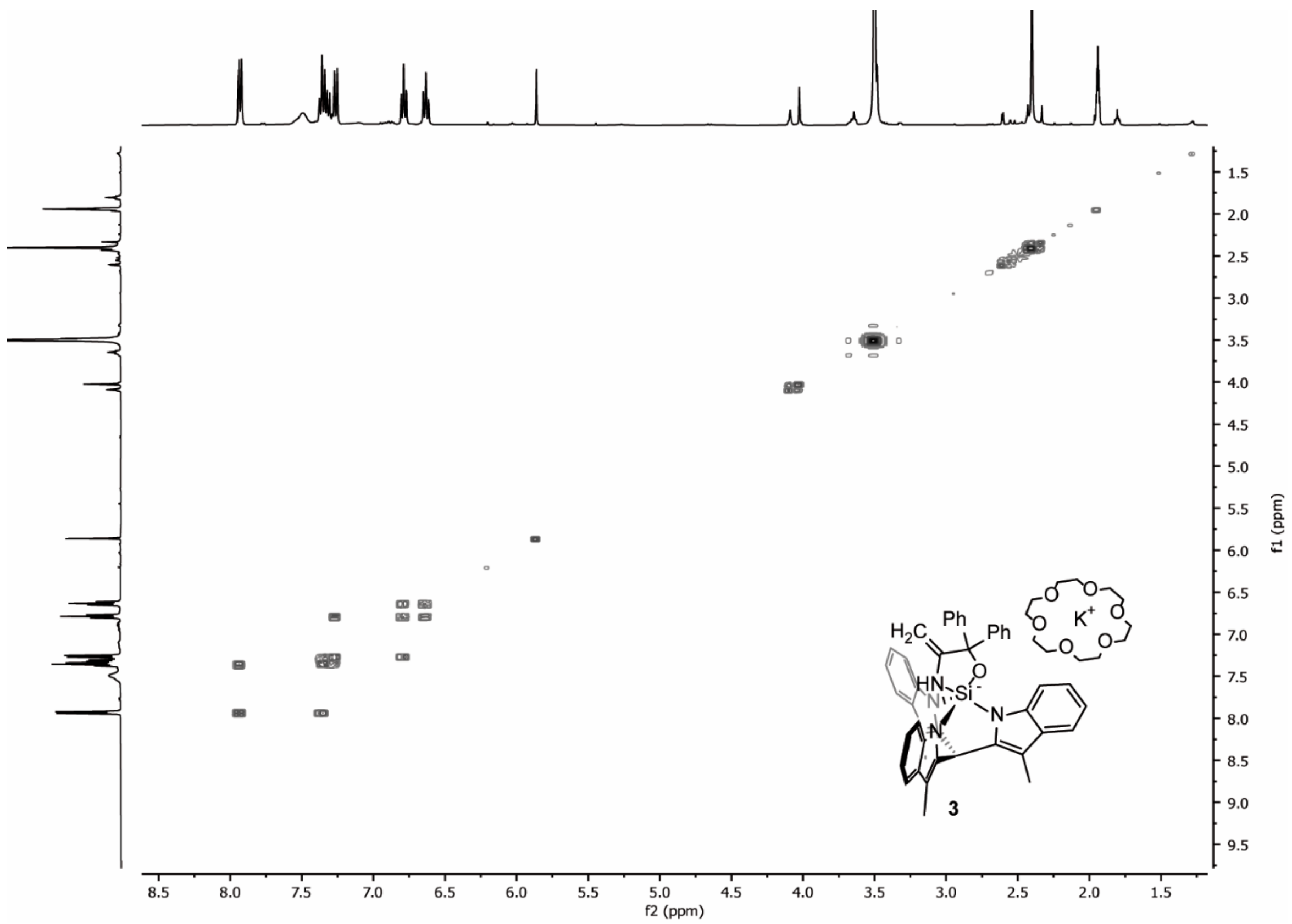

Figure S37. ^{1}H-COSY (400 MHz, CD_3CN, 25°C) spectrum of compound **3**.

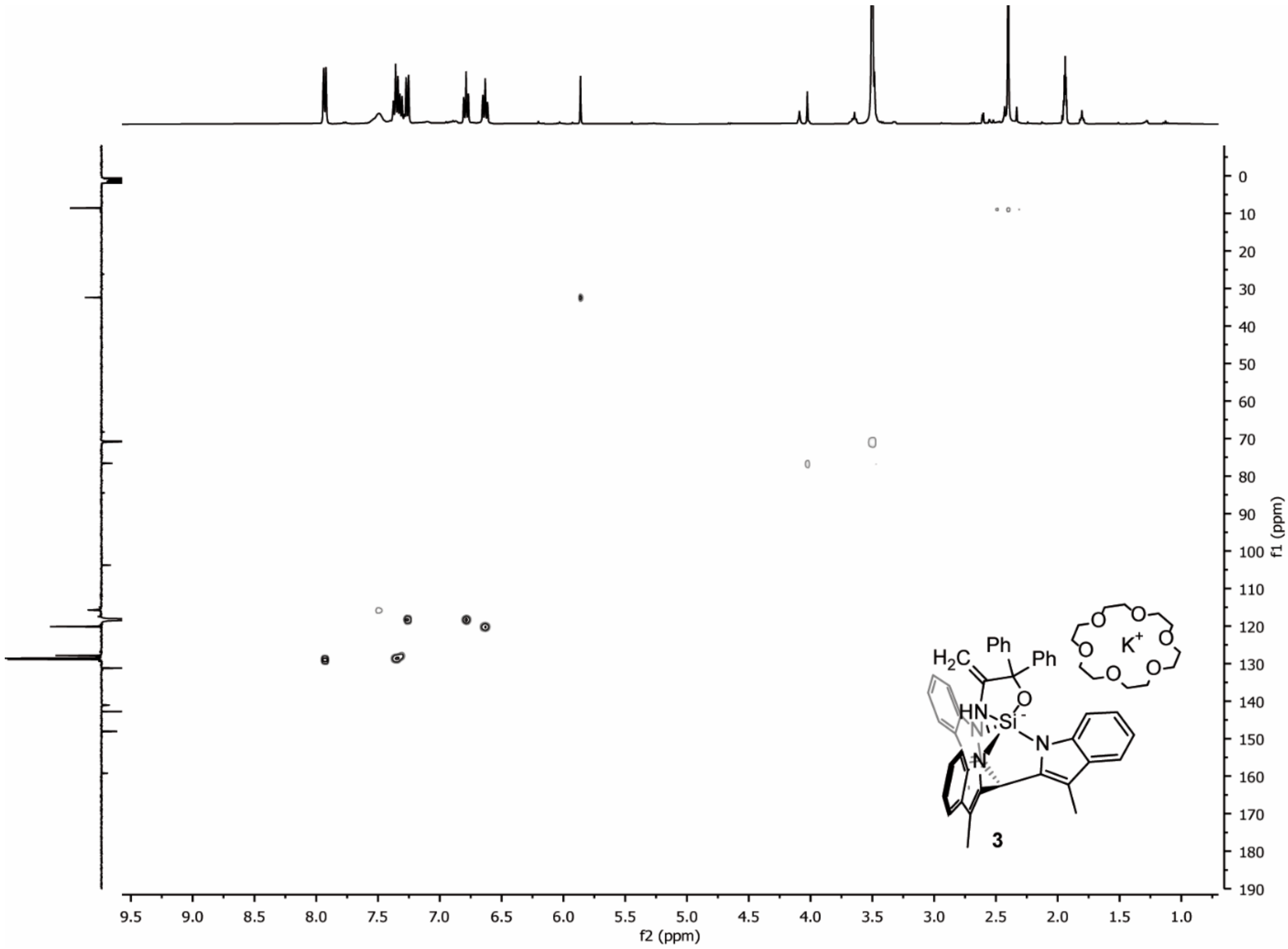

Figure S38. ^{1}H-^{13}C HSQC (400 MHz/100 MHz, CD_3CN, 25°C) spectrum of compound **3**.

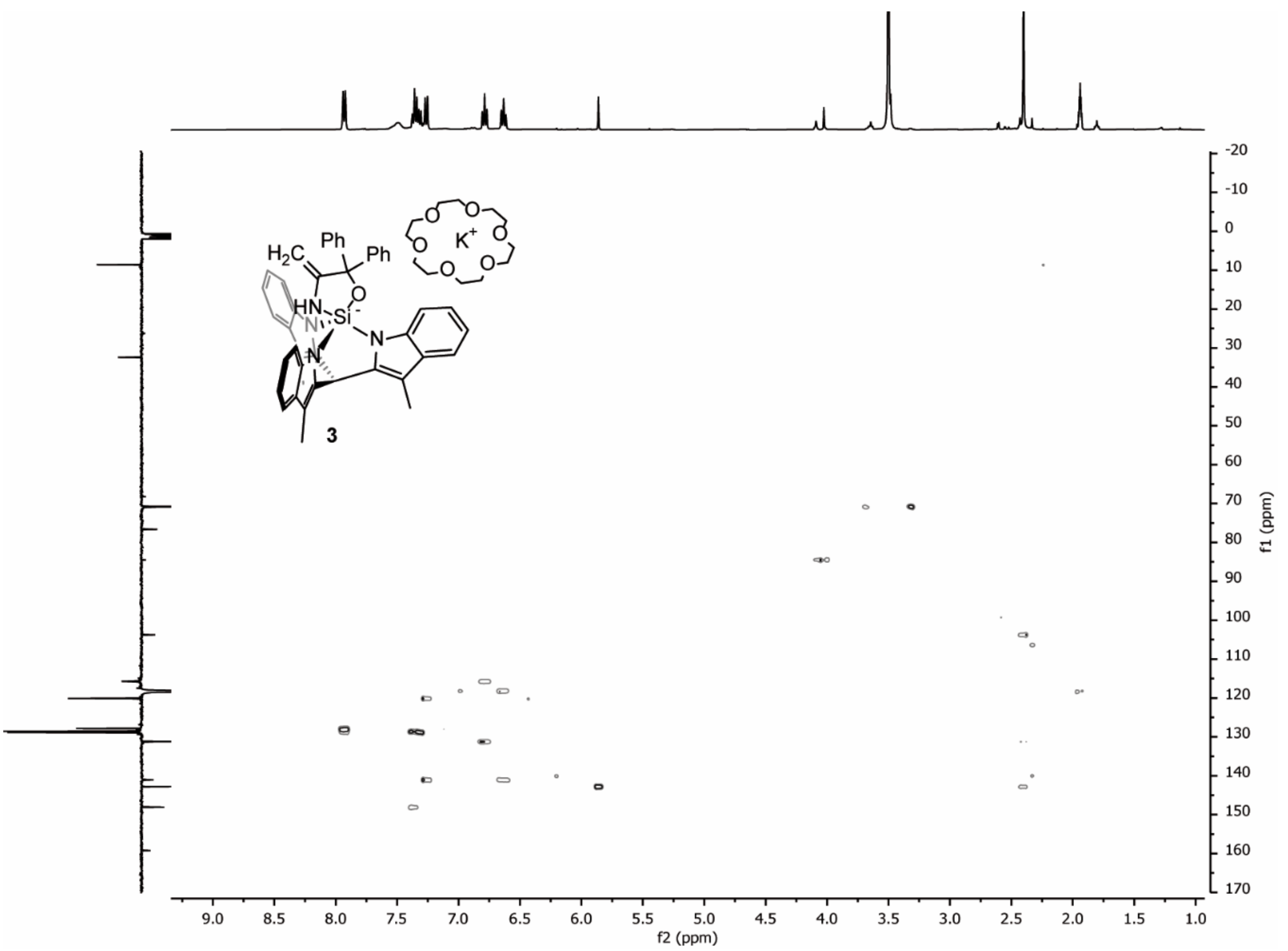

Figure S39. ^{1}H-^{13}C HMBC (400 MHz/100 MHz, CD_3CN, 25°C) spectrum of compound **3**.

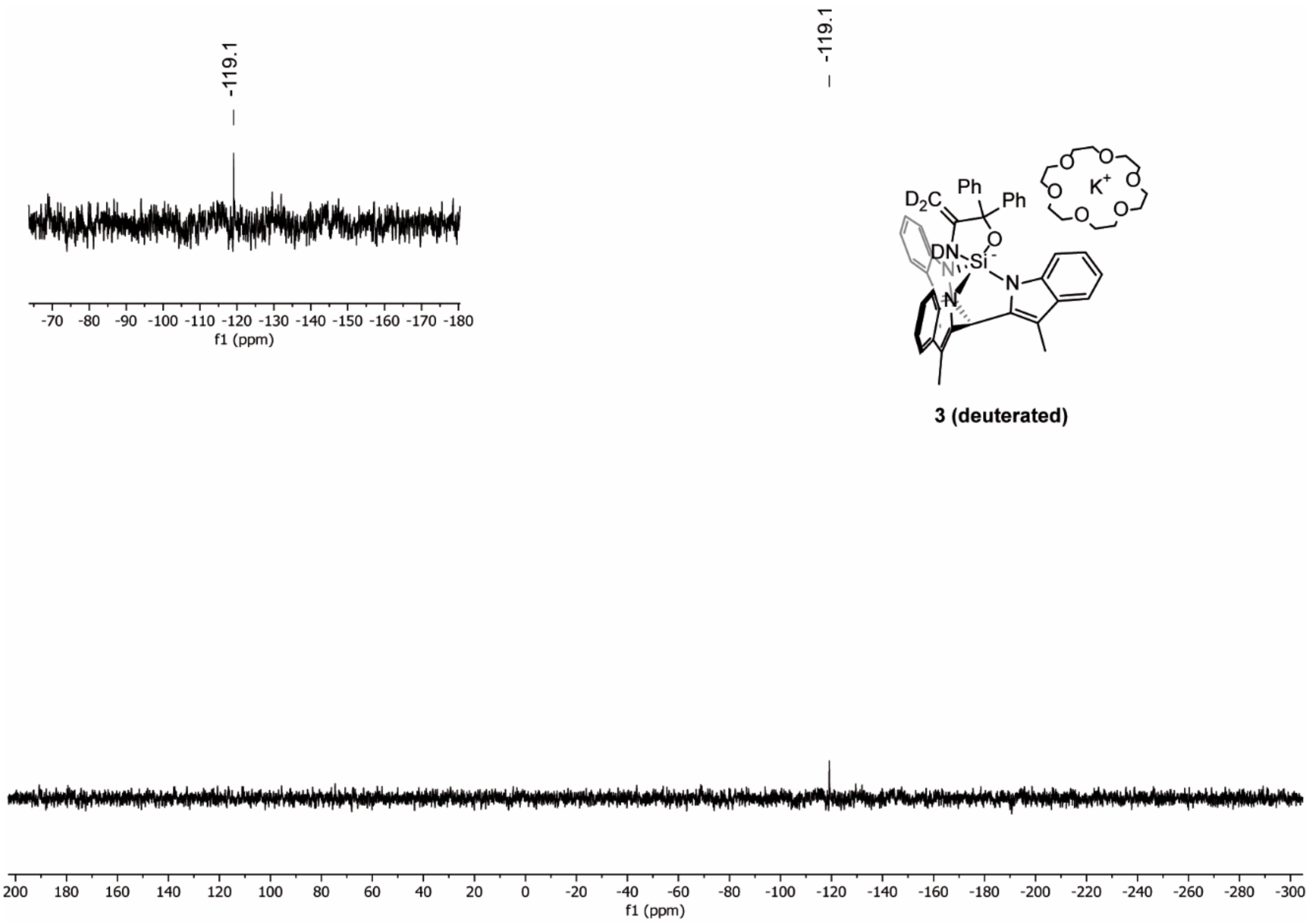

Figure S40. ^{29}Si-NMR (79 MHz, CD_2Cl_2, 25°C) spectrum of compound 3(deuterated) in: δ -119.11 (this NMR spectrum was recorded on the partially deuterated compound derived from CD_3CN).

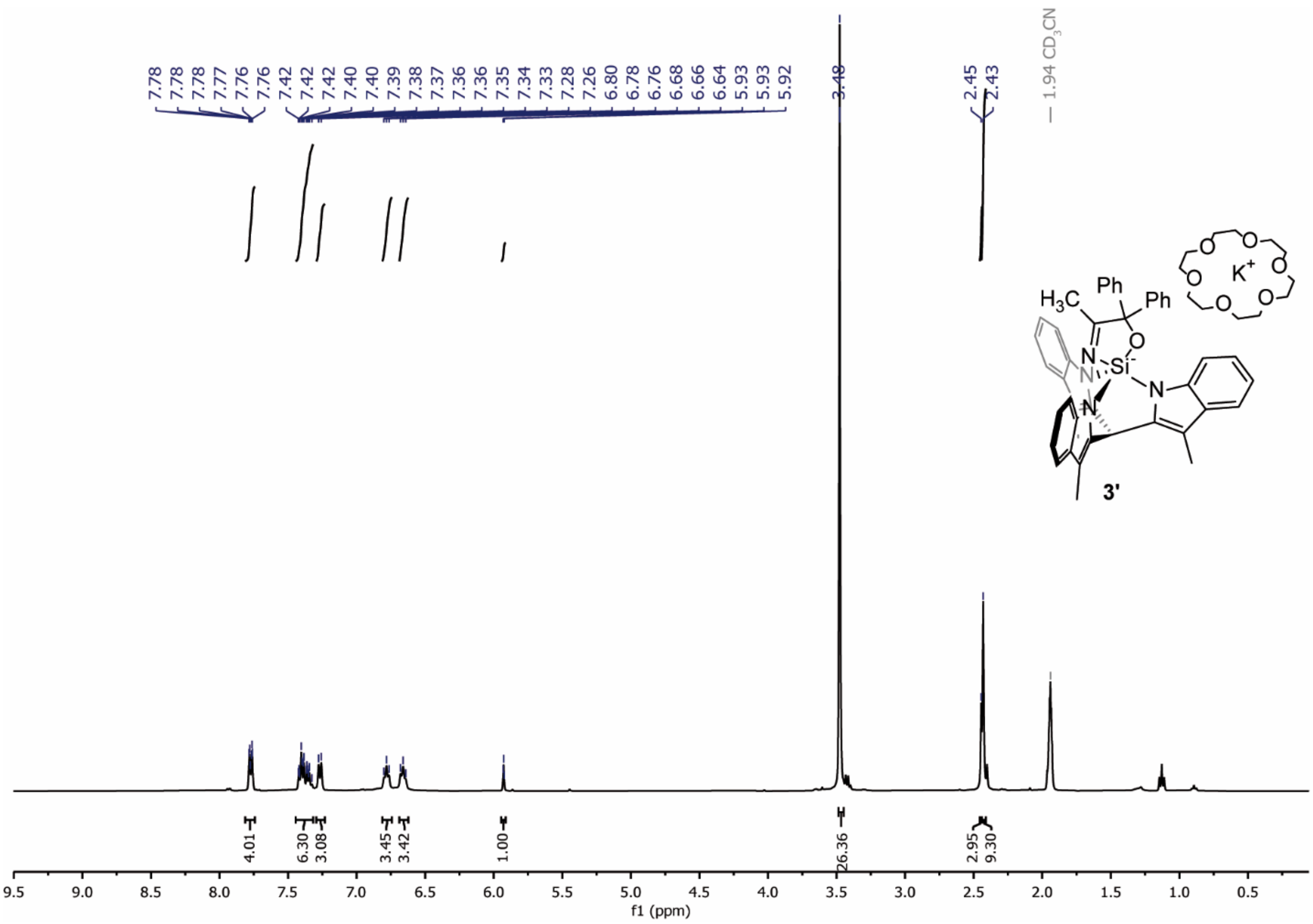

Figure S41. ^{1}H-NMR (400 MHz CD_3CN, 25°C) spectrum of compound **3’:** δ 7.77 (dt, *J* = 6.3, 1.5 Hz, 4H, Ph_2CO-H_{Ar}), 7.44 – 7.32 (m, 6H, Ph_2CO-H_{Ar}), 7.27 (d, *J* = 7.8 Hz, 3H, Indole-*H*), 6.78 (t, *J* = 7.5 Hz, 3H, Indole-*H*), 6.67 (d, *J* = 7.7 Hz, 3H, Indole-*H*), 5.93 (d, *J* = 1.5 Hz, 1H, Indole-C*H* linker), 3.48 (s, 24H, 18-Crown-6), 2.45 (s, 3H $NCCH_3$), 2.43 (s, 9H, Indole-CH_3), one Indole-*H* probably very broad.

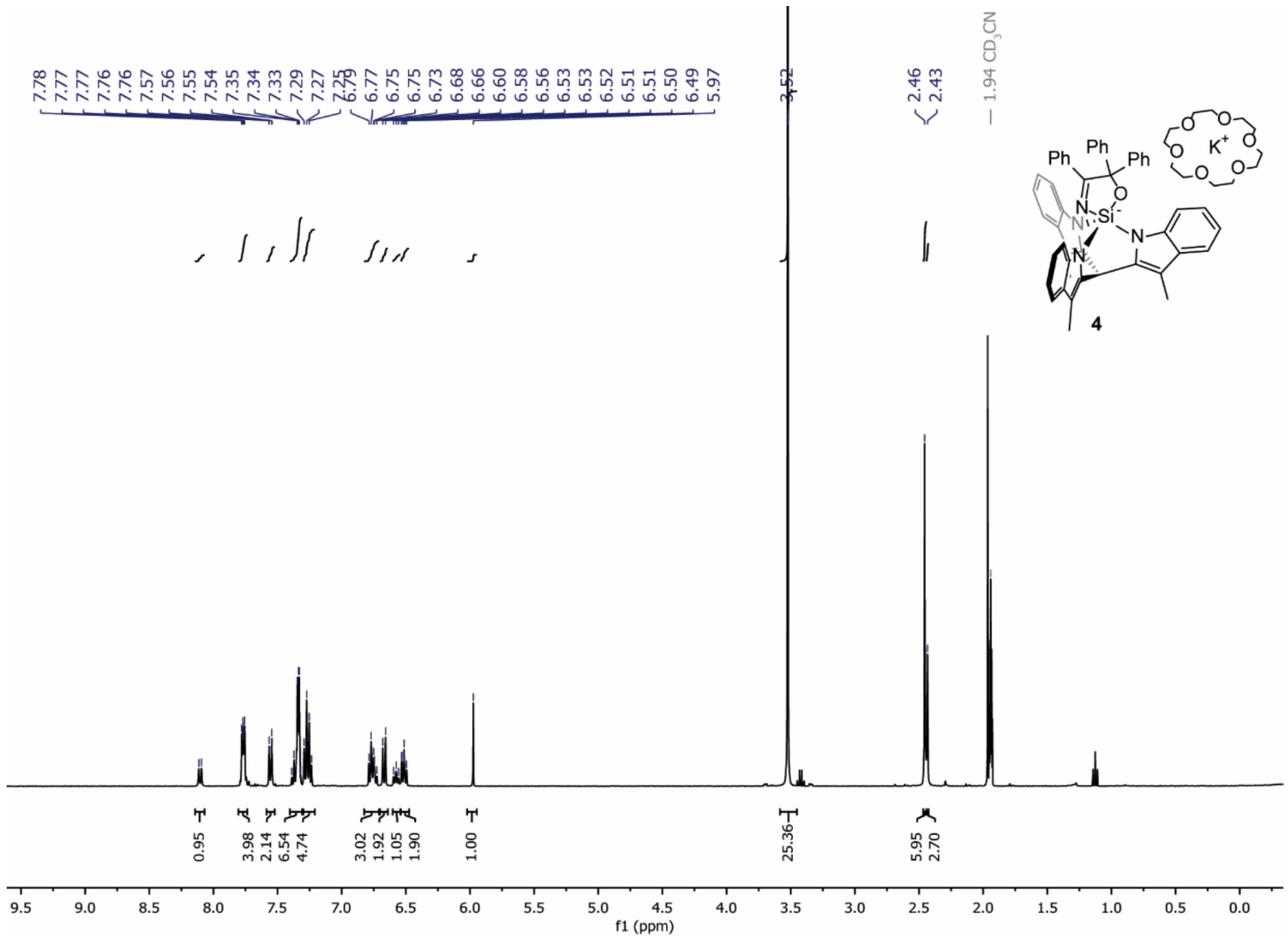

Figure S42. ^{1}H-NMR (400 MHz, CD_3CN, 25°C) spectrum of compound **4**: δ 8.10 (d, *J* = 8.3 Hz, 1H, Indole-*H*), 7.81 – 7.72 (m, 4H, Ph_2CO-H_{Ar}), 7.57 (d, *J* = 0.9 Hz, 1H, PhCN-H_{Ar}), 7.55 (t, *J* = 1.6 Hz, 1H, PhCN-H_{Ar}), 7.39 – 7.30 (m, 7H, Ph_2CO-H_{Ar} and PhCN-H_{Ar}), 7.26 (m, *J* = 7.9 Hz, 5H, Indole-*H* and PhCN-H_{Ar}), 6.77 (t, *J* = 7.3 Hz, 3H, Indole-*H*), 6.74 (t, *J* = 6.9 Hz, 1H, Indole-*H*), 6.67 (d, *J* = 8.3 Hz, 2H, Indole-*H*), 6.58 (t, *J* = 7.6 Hz, 1H, Indole-*H*), 6.51 (ddd, *J* = 8.2, 6.8, 1.3 Hz, 2H, Indole-*H*), 5.97 (s, 1H, Indole-C*H* linker), 3.52 (s, 24H, 18-crown-6), 2.46 (s, 6H, Indole-CH_3), 2.43 (s, 3H, Indole-CH_3).

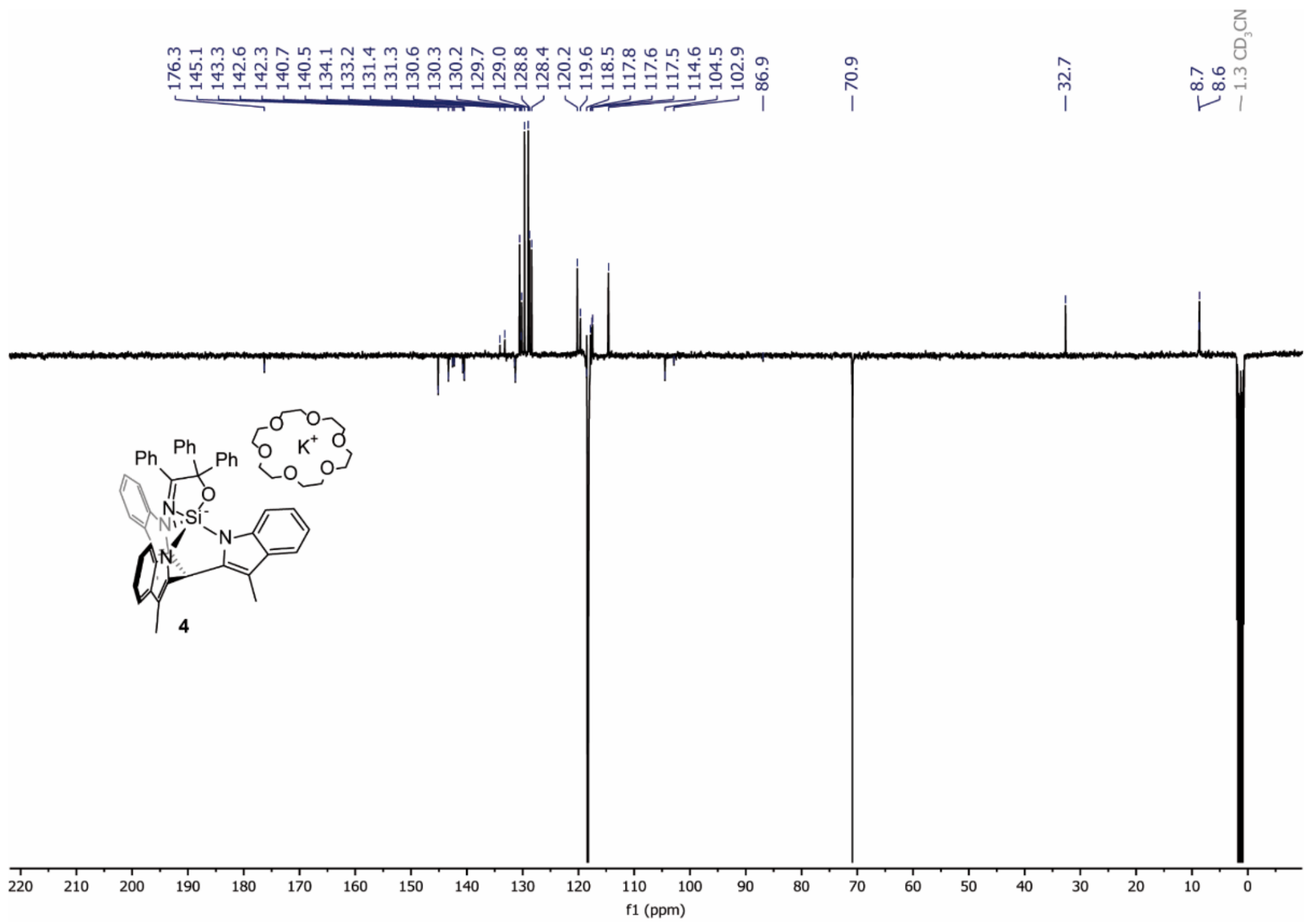

Figure S43. ^{13}C-APT- NMR (101 MHz) spectrum of compound **4**: δ 176.3 (s, Ph*C*N), 145.1 (s, Ph_2CO-*C*), 143.3 (s, C_{Indole}), 142.6(s, C_{Indole}), 142.3(s, PhCN-*C*), 140.7(s, C_{Indole}), 140.5(s, C_{Indole}), 131.4(s, CH_{Indole}), 131.3(s, CH_{Indole}), 130.6(s, PhCN-CH_{Ar}), 130.3(s, PhCN-CH_{Ar}), 130.2(s, Ph_2CO-CH_{Ar}), 129.0(s, Ph_2CO-CH_{Ar}), 128.8(s, Ph_2CO-CH_{Ar}), 128.4 (s, Ph_2CO-CH_{Ar}), 120.2(s, CH_{Indole}), 119.6(s, CH_{Indole}), 118.5(s, CH_{Indole}), 117.8(s, CH_{Indole} or PhCN-CH_{Ar}), 117.6(s, CH_{Indole} or PhCN-CH_{Ar}), 117.5(s, CH_{Indole} or PhCN-CH_{Ar}), 114.6(s, CH_{Indole}), 104.5(s, C_{Indole}), 102.9(s, C_{Indole}), 86.9 (s, Ph_2CO) 70.9(s, CH_2,18-crown-6), 32.7(s, CH_{Indole} linker), 8.7(s, $CH_{3\text{-}Indole}$), 8.6(s, $CH_{3\text{-}Indole}$).

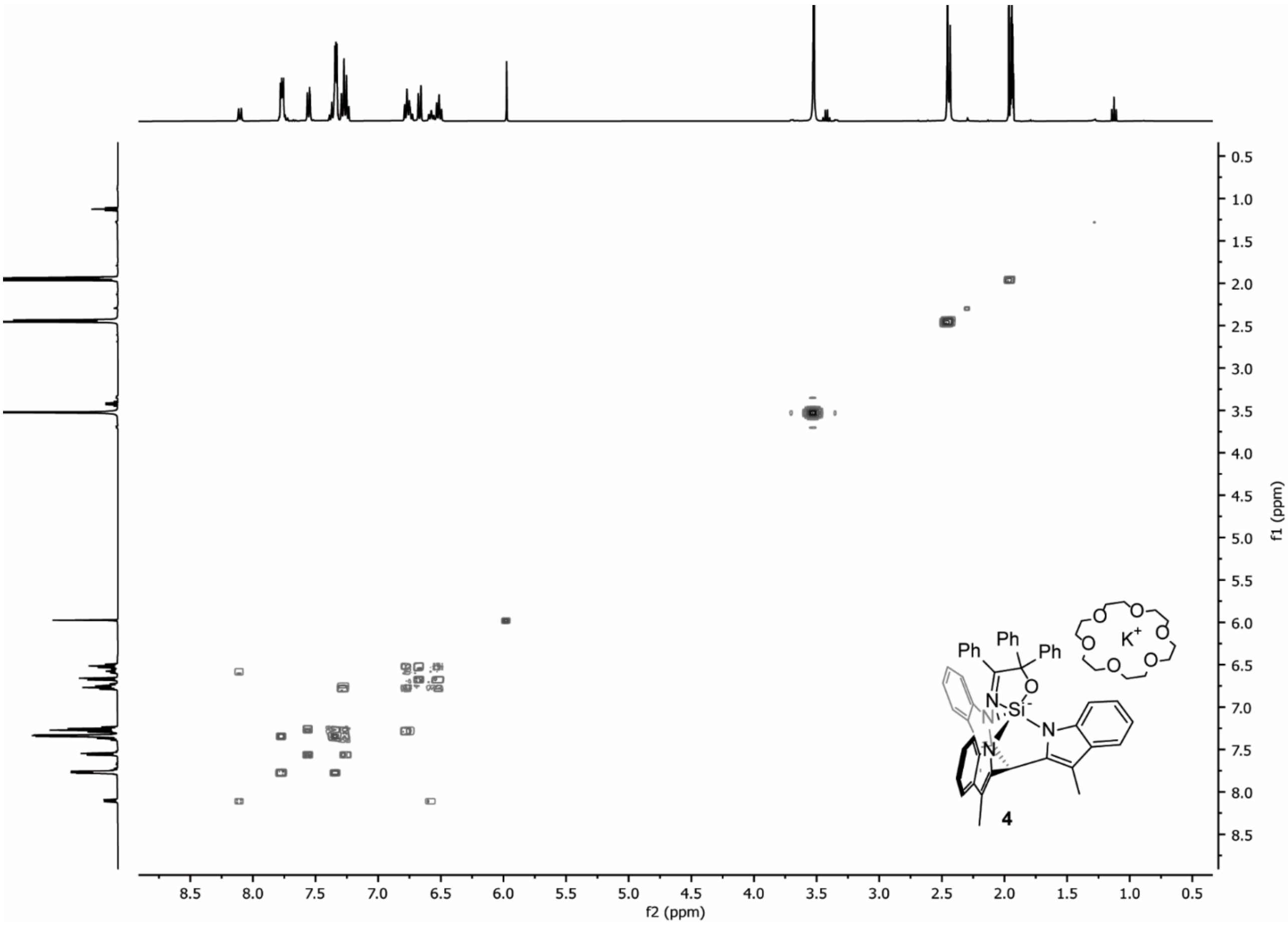

Figure S44. ^{1}H-COSY (400 MHz, CD_3CN, 25°C) spectrum of compound **4**.

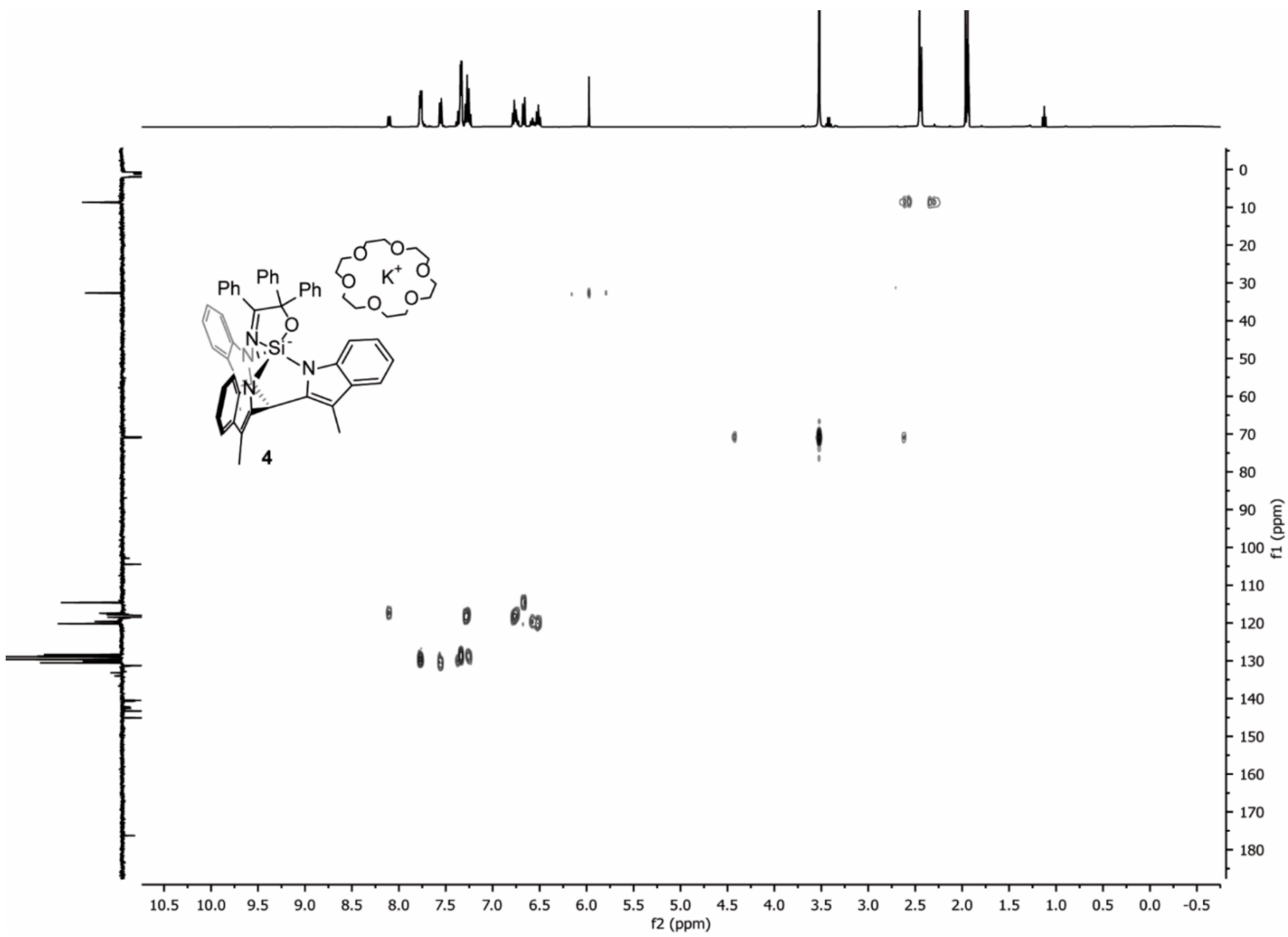

Figure S45. ^{1}H-^{13}C HSQC (400 MHz/100 MHz, CD_3CN, 25°C) spectrum of compound **4**.

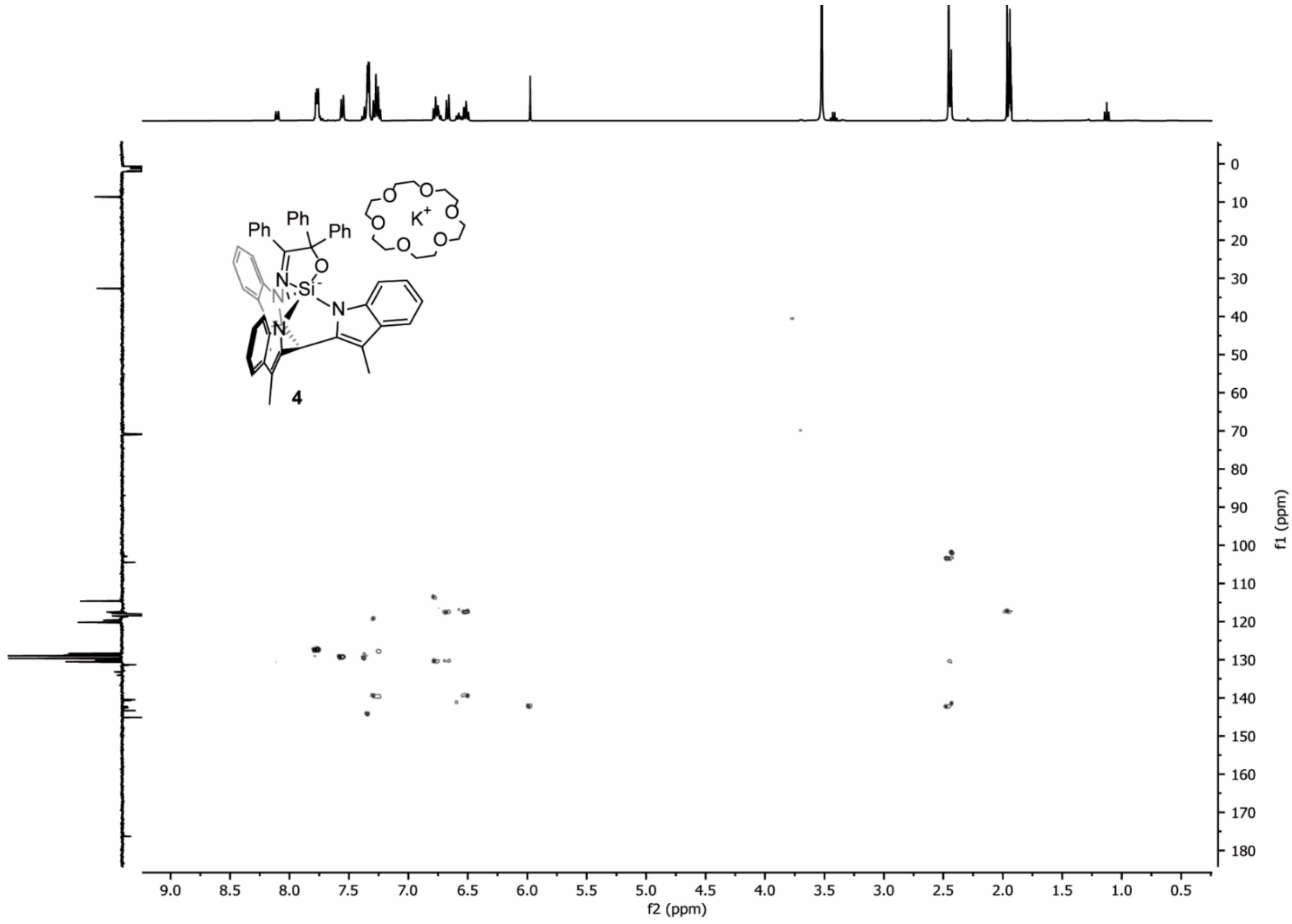

Figure S46. ^{1}H-^{13}C HMBC (400 MHz/100 MHz, CD_3CN, 25°C) spectrum of compound **4**.

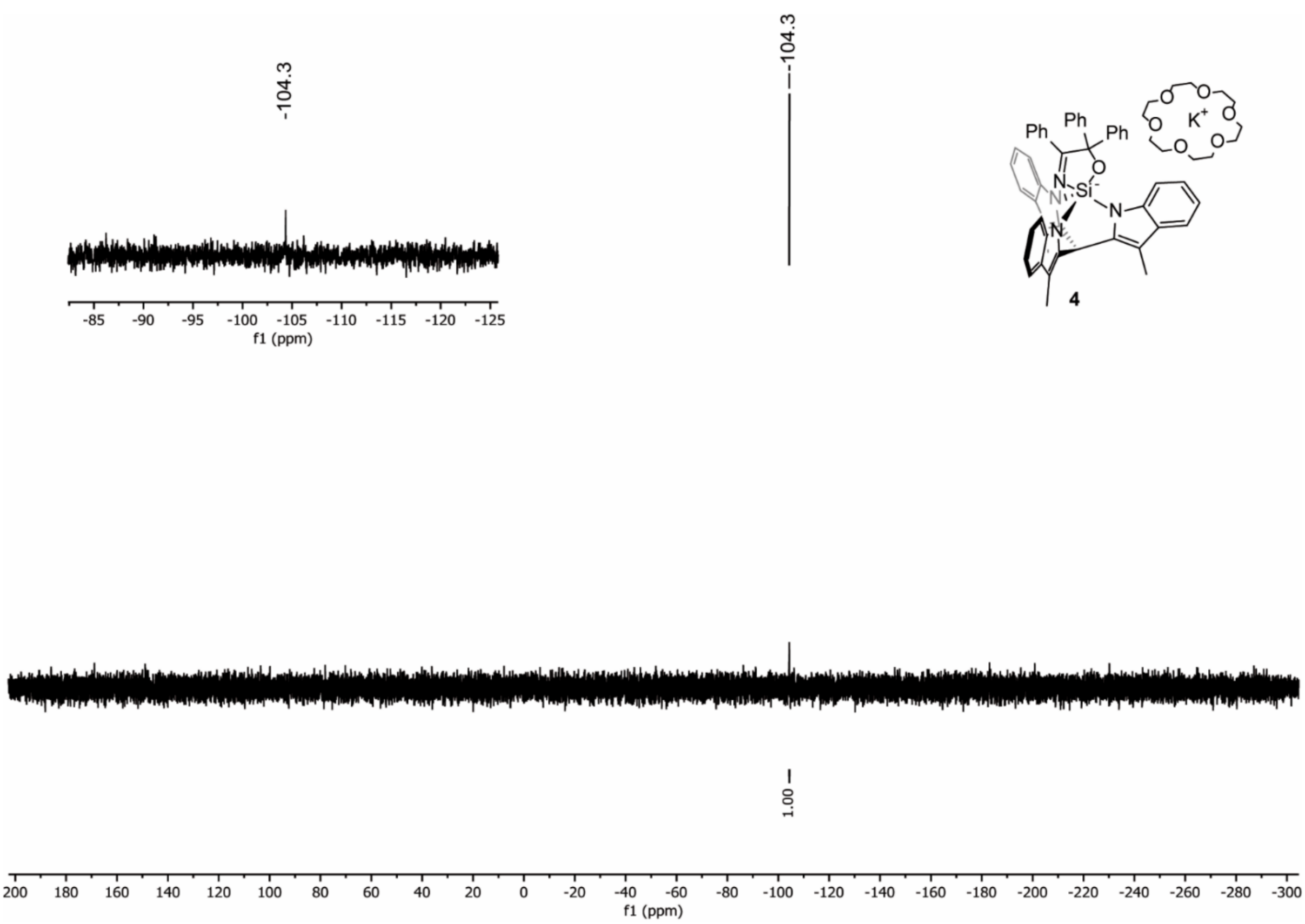

Figure S47. ^{29}Si-NMR (79 MHz, CD_3CN, 25°C) spectrum of compound **4**.

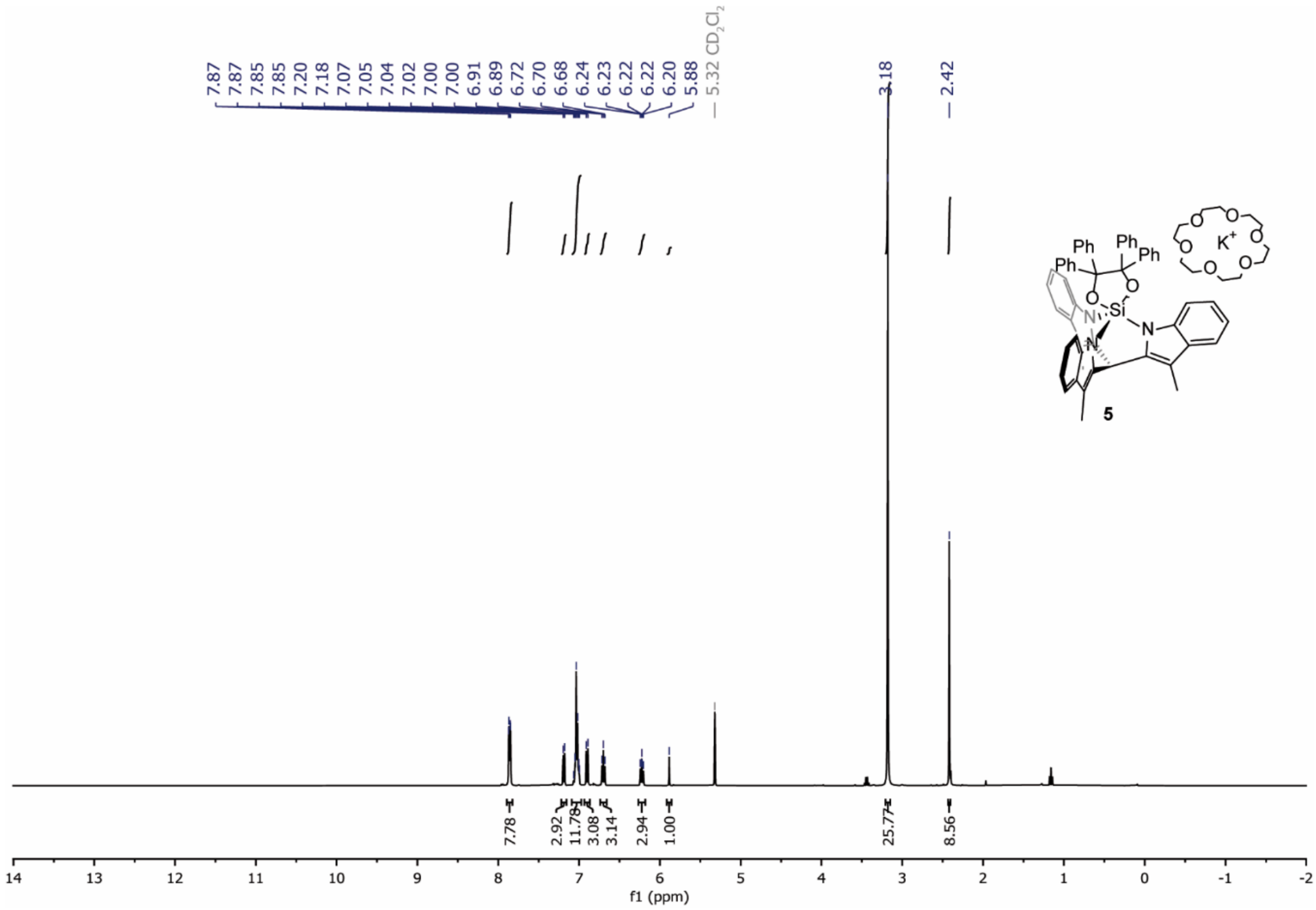

Figure S48. ^{1}H-NMR (400 MHz, CD_2Cl_2, 25°C) spectrum of compound **5**: δ 7.92 – 7.82 (m, 8H, Ph_2CO-*H$_{Ar}$*), 7.19 (d, *J* = 7.7 Hz, 3H, Indole-*H*), 7.07 – 6.98 (m, 12H, Ph_2CO-*H$_{Ar}$*), 6.90 (d, *J* = 8.5 Hz, 3H, Indole-*H*), 6.70 (t, *J* = 7.3 Hz, 3H, Indole-*H*), 6.29 – 6.16 (m, 3H, Indole-*H*), 5.88 (s, 1H, Indole-C*H* linker), 3.18 (s, 24H, 18-crown-6), 2.42 (s, 9H, Indole-C*H$_3$*).

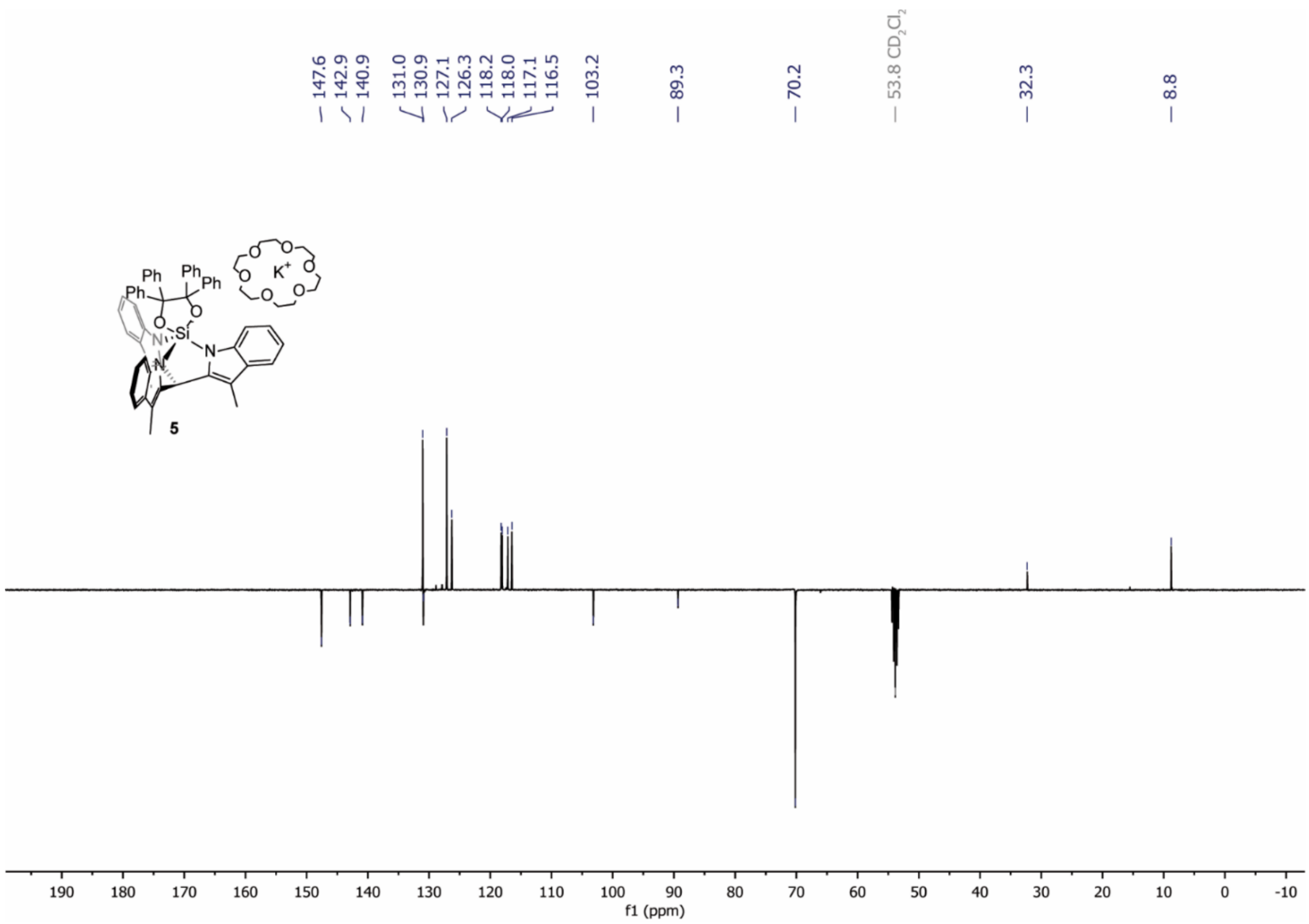

Figure S49. ^{13}C-APT-NMR (APT, 101 MHz, CD_2Cl_2, 25°C) spectrum of compound **5**: δ 147.6(s, Ph_2CO-*C*), 142.9(s, *C*$_{Indole}$), 140.9(s, *C*$_{Indole}$), 131.0(s, Ph_2CO-*CH$_{Ar}$*), 130.9(s, *C*$_{Indole}$), 127.1(s, Ph_2CO-*CH$_{Ar}$*), 126.3(s, Ph_2CO-*CH$_{Ar}$*), 118.2(s, *C*H$_{Indole}$), 118.0(s, *C*H$_{Indole}$), 117.1(s, *C*H$_{Indole}$), 116.5(s, *C*H$_{Indole}$), 103.2(s, *C*$_{Indole}$), 89.3 (Ph_2*C*O), 70.2 (s, *C*H_2,18-crown-6), 32.3 (s, *C*H$_{Indole}$ linker), 8.8(s, *C*H$_{3\text{-}Indole}$).

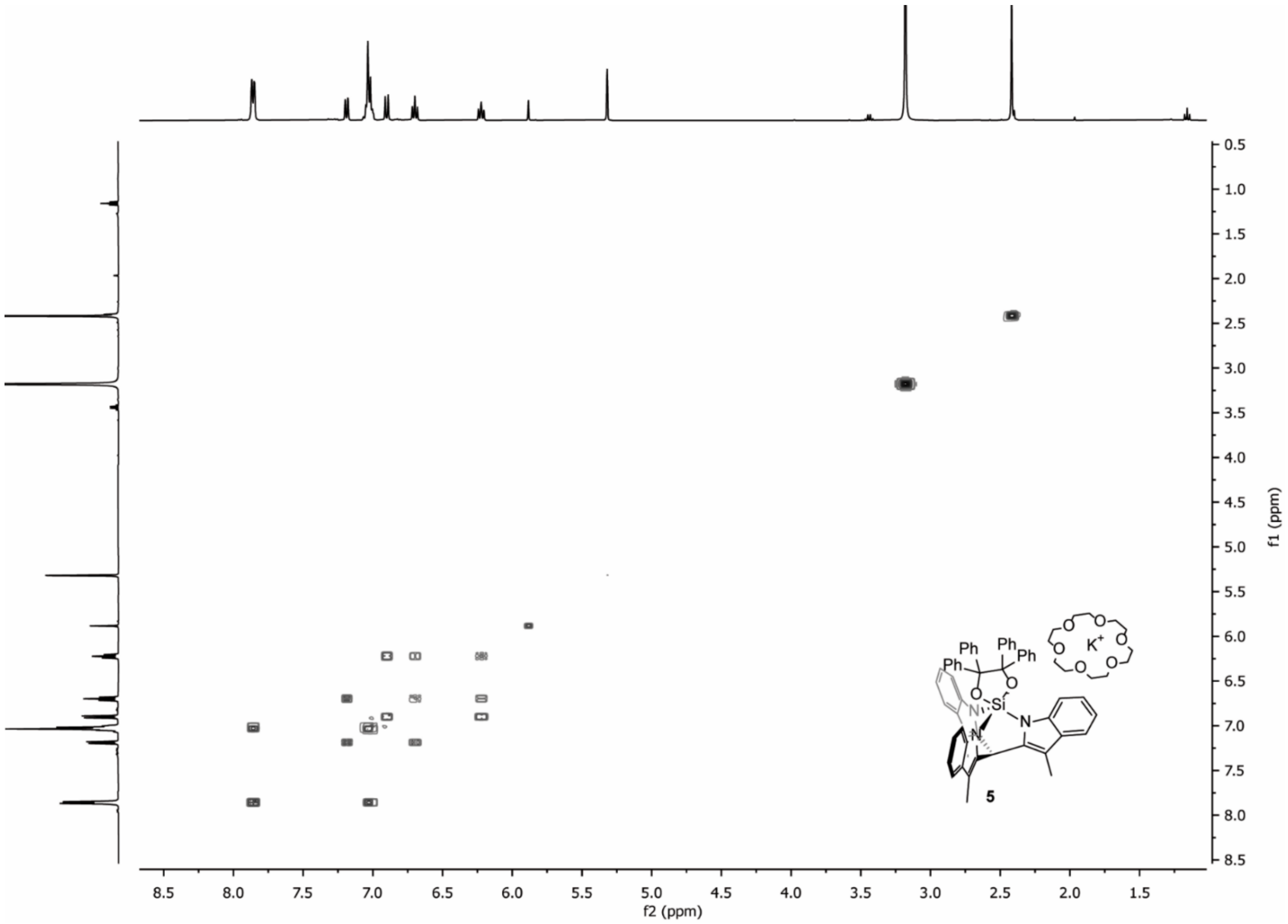

Figure S50. ^{1}H-COSY (400 MHz, CD_3CN, 25°C) spectrum of compound **5**.

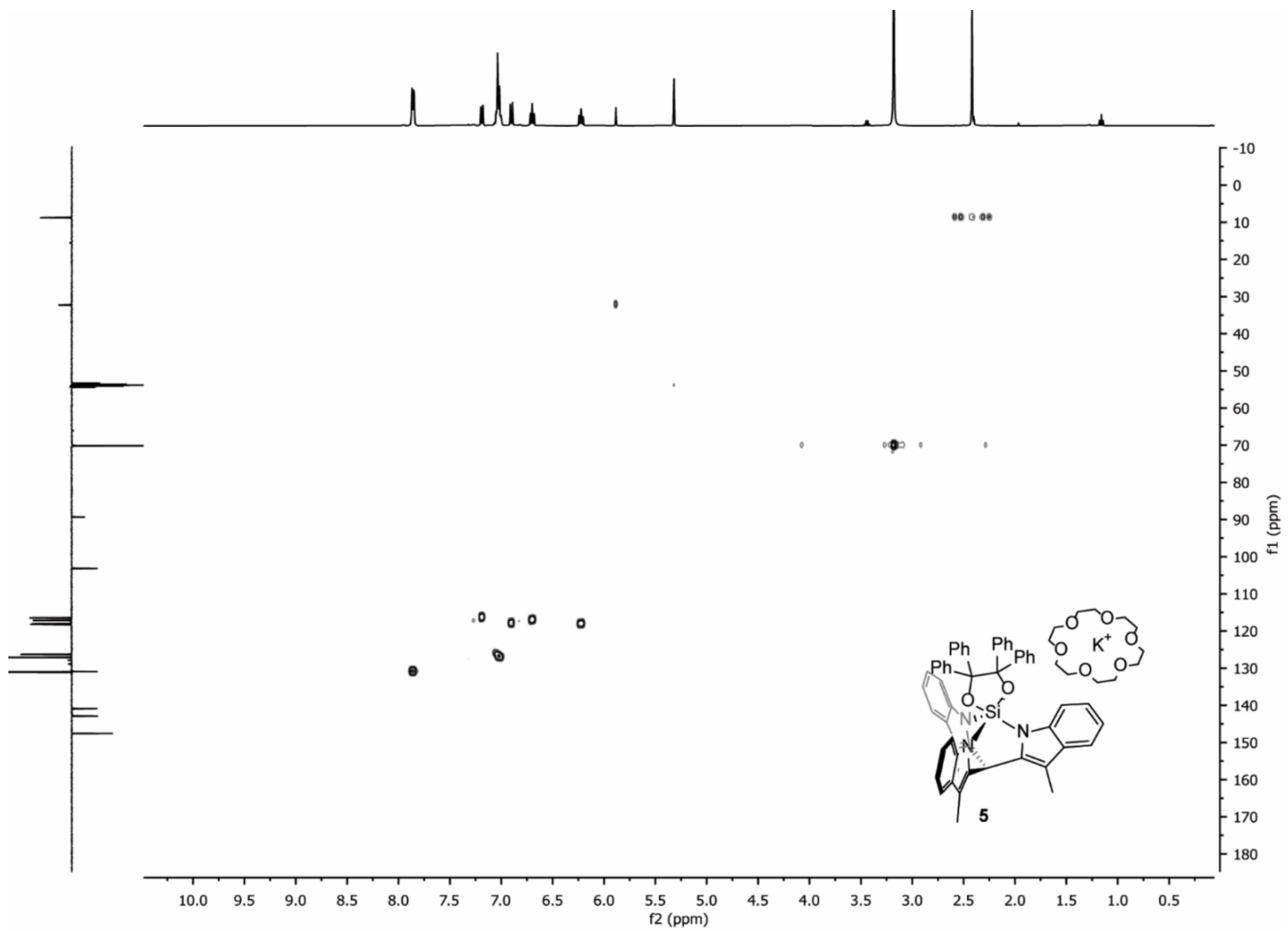

Figure S51. ^{1}H-^{13}C HSQC spectrum (400 MHz/100 MHz, CD_2Cl_2, 25°C) of compound **5**.

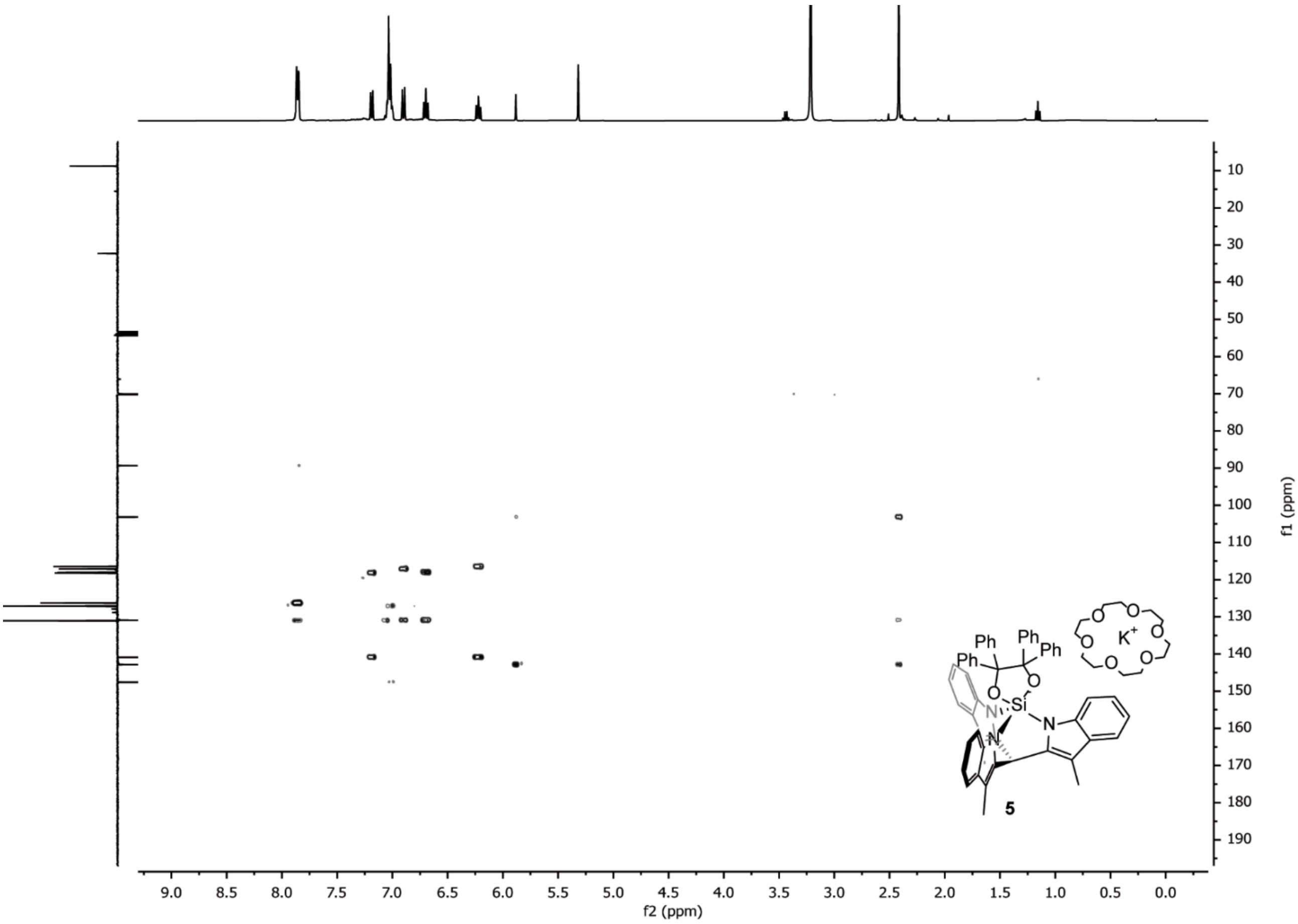

Figure S52. ^{1}H-^{13}C HMBC(400 MHz/100 MHz, CD_2Cl_2, 25°C) spectrum of compound **5**.

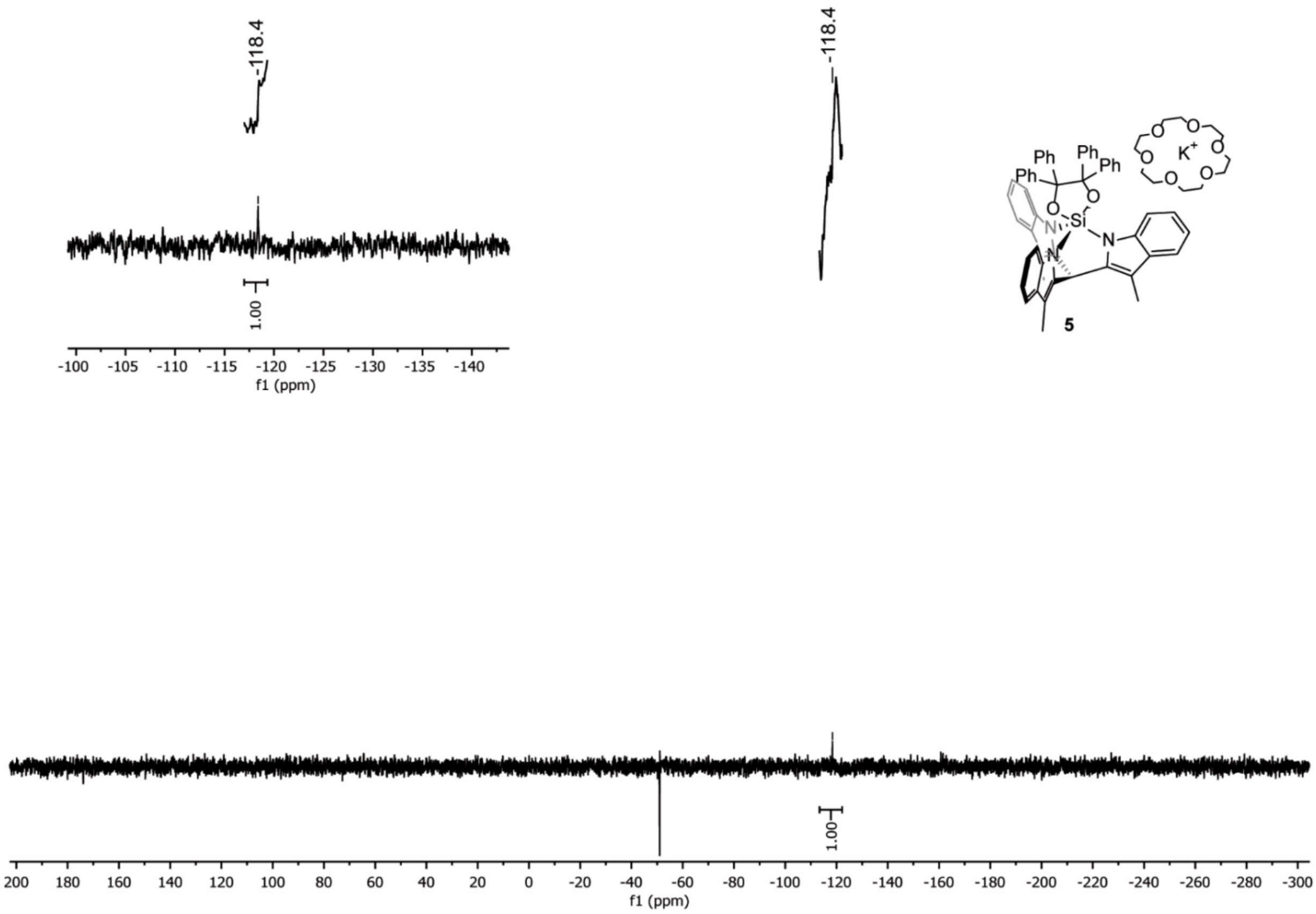

Figure S53. ^{29}Si-NMR (79 MHz, CD_2Cl_2, 25°C) spectrum of compound **5**. Peak around -50 ppm: Center glitch artifact at the center of the measurement window.

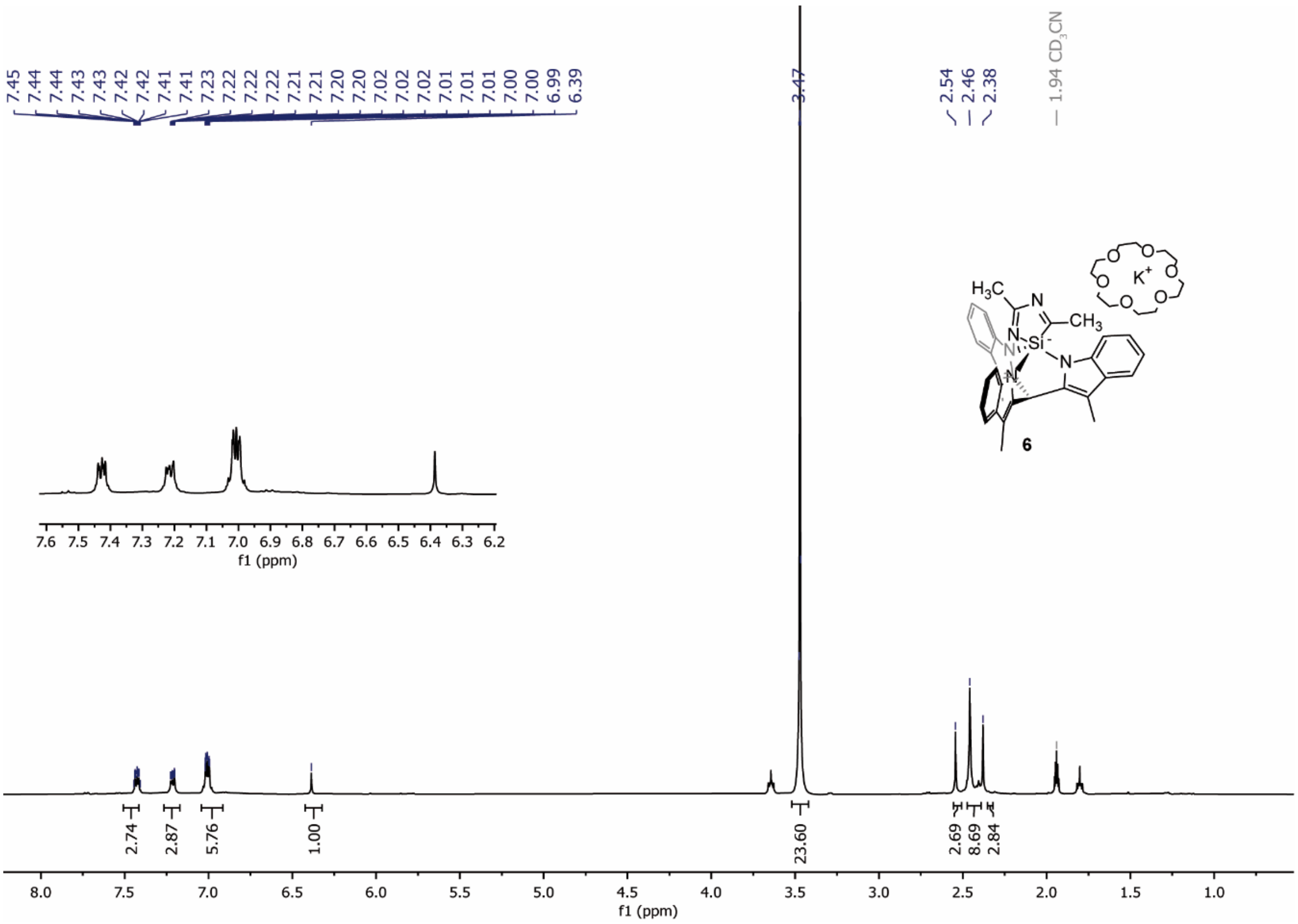

Figure S54. ^{1}H-NMR (400 MHz, CD_3CN, 25°C) spectrum of compound **6**: δ 7.42 (ddt, J = 6.2, 4.0, 2.0 Hz, 3H, Indole-*H*), 7.25 – 7.18 (m, 3H, Indole-*H*), 7.04 – 6.96 (m, 6H, Indole-*H*), 6.39 (s, 1H, Indole-C*H* linker), 3.47 (s, 24H,18-crown-6), 2.54 (s, 3H, Cycle-CH_3), 2.46 (s, 9H, Indole-CH_3), 2.38 (s, 3H, Cycle- CH_3).

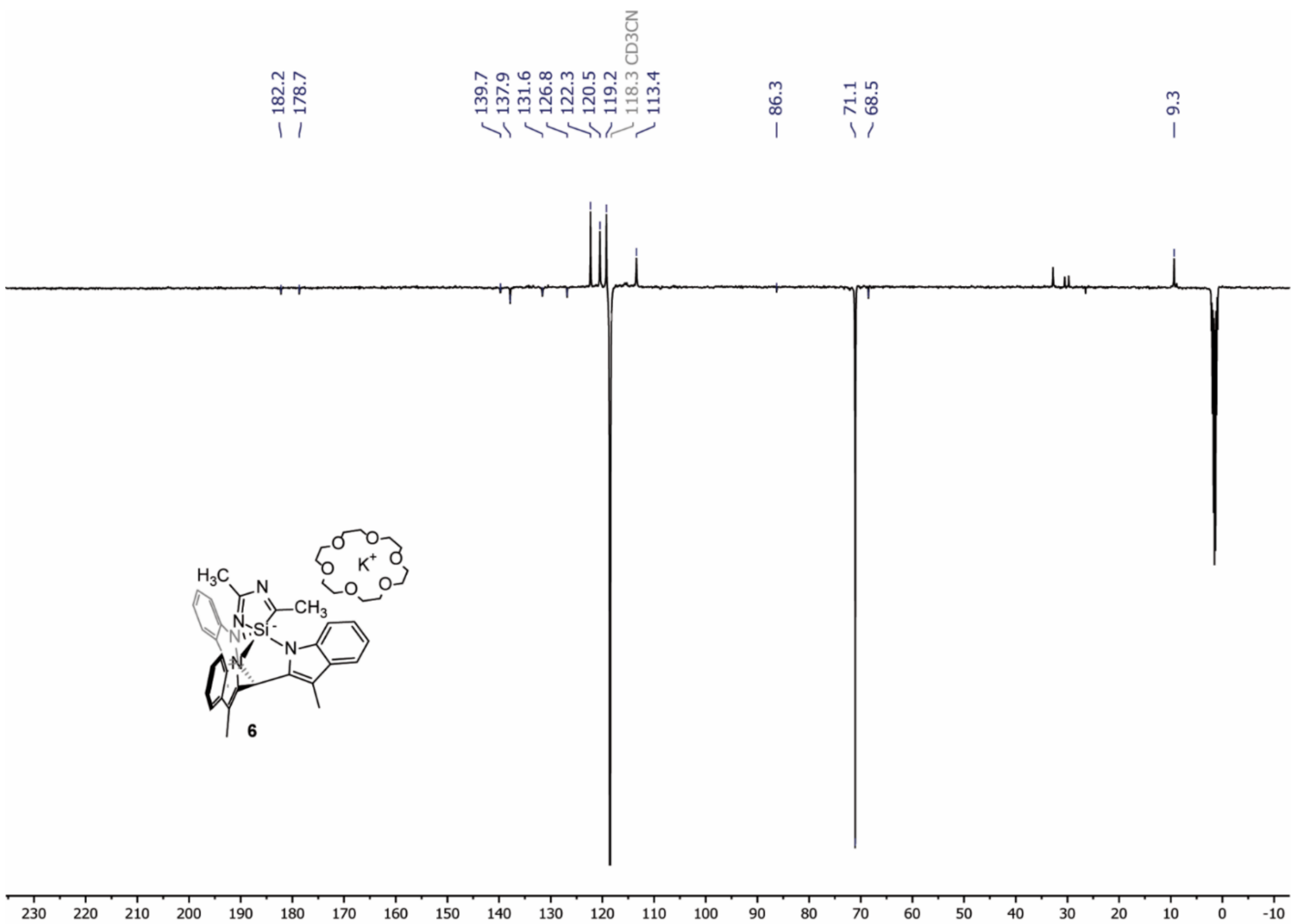

Figure S55. ^{13}C-APT- NMR (APT, 101 MHz, CD_3CN, 25°C) spectrum of compound **6:** δ 182.2 (s, Cycle *C*=N), 178.7 (s, Cycle *C*=N), 139.7(s, C_{Indole}), 137.9(s, C_{Indole}), 131.6(s, C_{Indole}), 122.3 (s, CH_{Indole}), 120.5 (s, CH_{Indole}), 119.2(s, CH_{Indole}), 113.4 (s, CH_{Indole}), 71.1(s, CH_2,18-crown-6), 32.8(s, CH_{Indole} linker), 30.5(s, Cycle CH_3), 29.7(s, Cycle CH_3), 9.3(s, $CH_{3\text{-}Indole}$) ($C_{Indole\ at}$ 108.5 ppm detected via HMBC measurement).

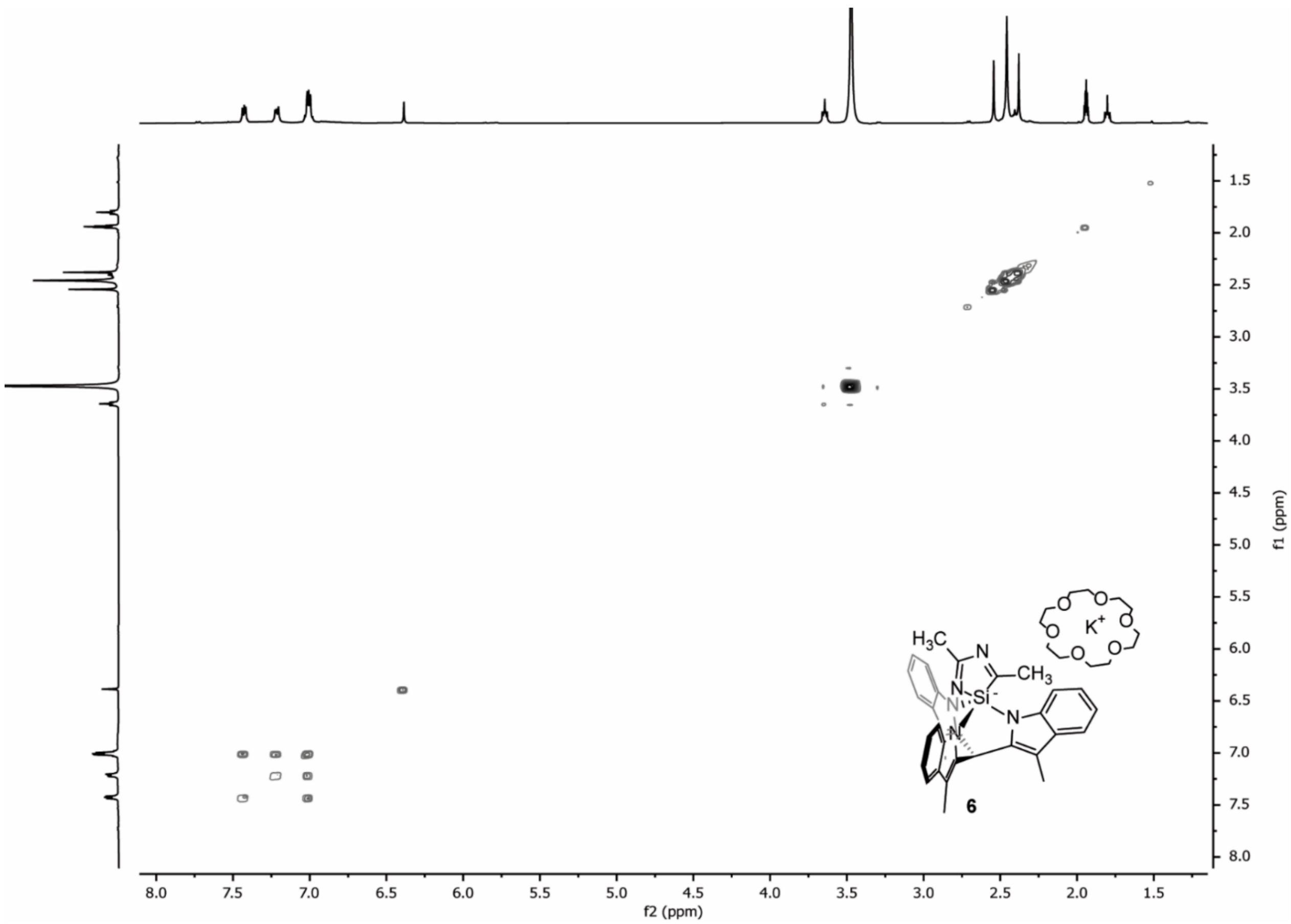

Figure S56. ^{1}H-COSY (400 MHz, CD_3CN, 25°C) spectrum of compound **6**.

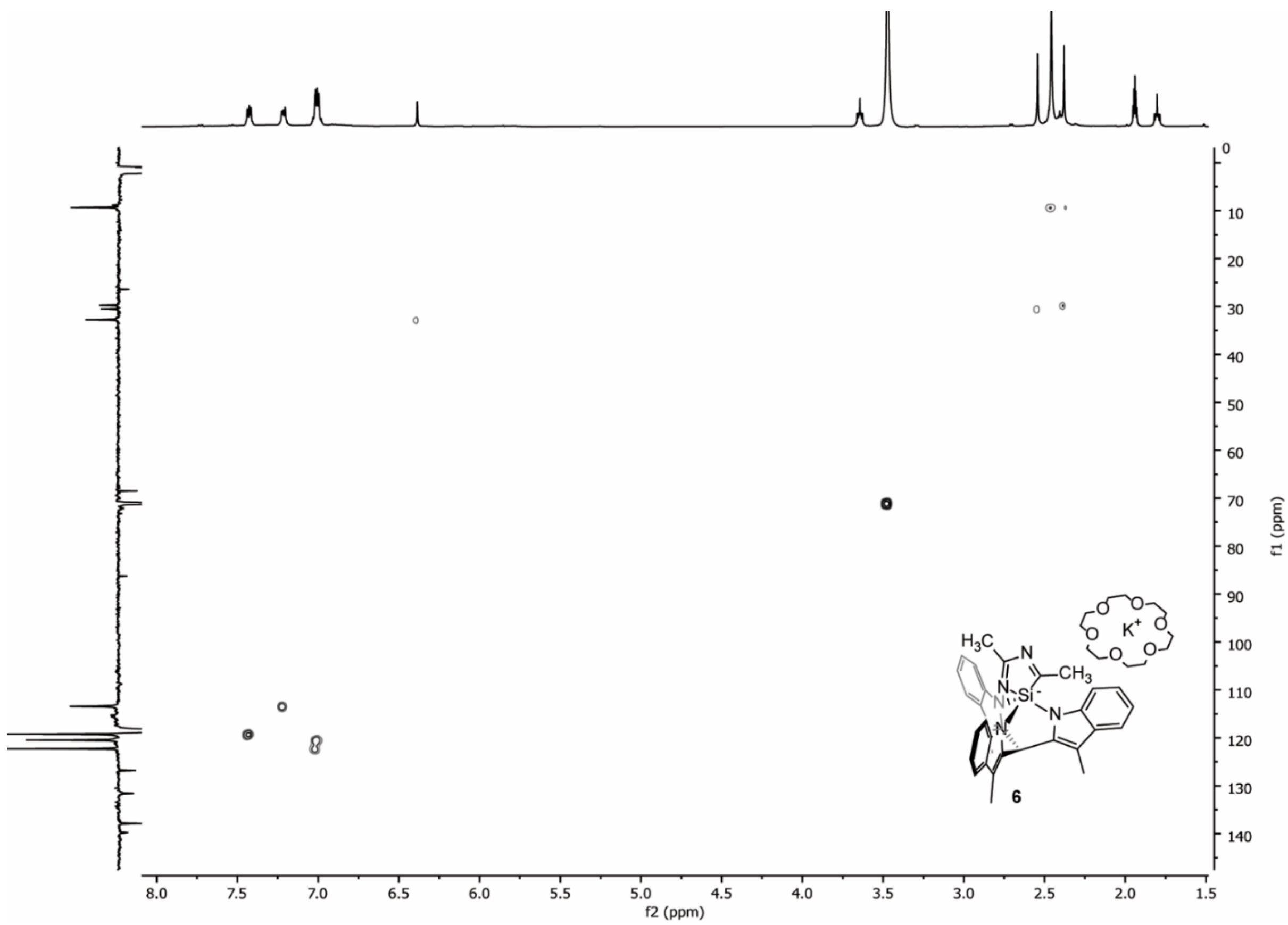

Figure S57. ^{1}H-^{13}C HSQC (400 MHz/100 MHz, CD_3CN, 25°C) spectrum of compound **6**.

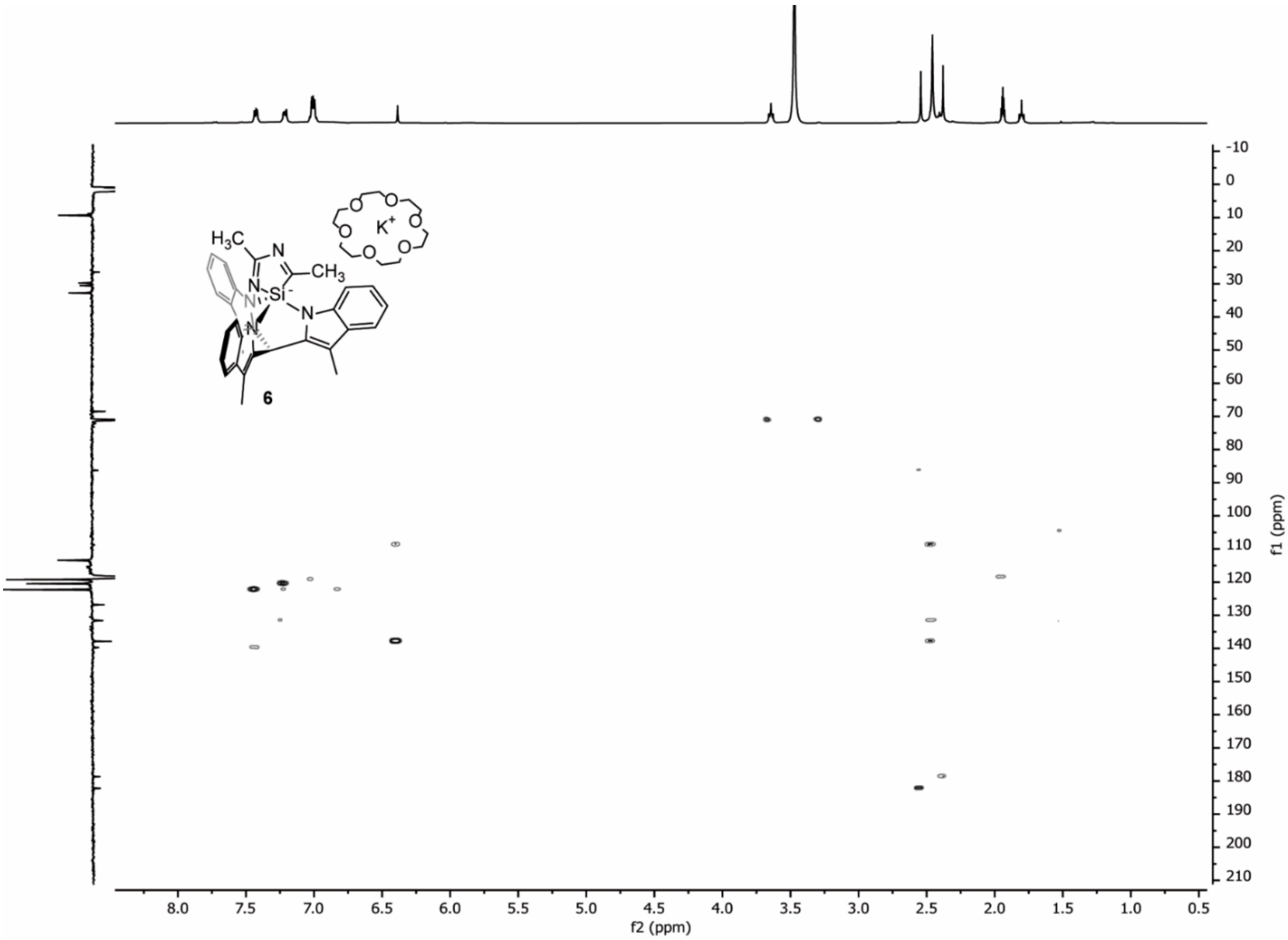

Figure S58. ^{1}H-^{13}C HMBC (400 MHz/100 MHz, CD_3CN, 25°C)spectrum of compound **6**.

6. References

1 L. Witteman, T. Evers, M. Lutz, M. E. Moret, "A Free Silanide from Nucleophilic Substitution at Silicon(II)" *Chemistry - A European Journal* **2018**, *24*, 12236–12240.

2 P. A. Benzan Lantigua, M. Lutz, M. E. Moret, "Polar X−H Bond (X=O, S, N) Activation at a Cage Silanide" *Angew. Chem. Int. Ed.* **2024**, *63*.

3 R. S. Ghadwal, H. W. Roesky, S. Merkel, J. Henn, D. Stalke, "Lewis base stabilized dichlorosilylene" *Angew. Chem. Int. Ed.* **2009**, *48*, 5683–5686.

4 A. M. M. Schreurs, X. Xian, L. M. J. Kroon-Batenburg. "EVAL15: a diffraction data integration method based on ab initio predicted profiles". *J. Appl. Cryst*. **2010**, *43*, 70-82.

5 L. Krause, R. Herbst-Irmer, G.M. Sheldrick, D. Stalke. "Comparison of silver and molybdenum microfocus X-ray sources for single-crystal structure determination". *J. Appl. Cryst.,* **2015**, *48*, 3-10.

6 G. M. Sheldrick. "*SHELXT* - Integrated space-group and crystal-structure determination". *Acta Cryst.,* **2015**, *A71*, 3-8.

7 G. M. Sheldrick. "Crystal structure refinement with *SHELXL*". *Acta Cryst.* **2015**. *C71*, 3-8.

8 A. L. Spek. "Structure validation in chemical crystallography". *Acta Cryst.* **2009**. *D65*, 148-155.

9 M. Sevvana, M. Ruf, I. Uson, G.M. Sheldrick, R. Herbst-Irmer. "Non-merohedral twinning: from minerals to proteins". *Acta Cryst.* **2019**. *D75*, 1040-1050.

10 Gaussian 16, Revision C.02, M. J. Frisch, G. W. Trucks, H. B. Schlegel, G. E. Scuseria, M. A. Robb, J. R. Cheeseman, G. Scalmani, V. Barone, G. A. Petersson, H. Nakatsuji, X. Li, M. Caricato, A. V. Marenich, J. Bloino, B. G. Janesko, R. Gomperts, B. Mennucci, H. P. Hratchian, J. V. Ortiz, A. F. Izmaylov, J. L. Sonnenberg, D. Williams-Young, F. Ding, F. Lipparini, F. Egidi, J. Goings, B. Peng, A. Petrone, T. Henderson, D. Ranasinghe, V. G. Zakrzewski, J. Gao, N. Rega, G. Zheng, W. Liang, M. Hada, M. Ehara, K. Toyota, R. Fukuda, J. Hasegawa, M. Ishida, T. Nakajima, Y. Honda, O. Kitao, H. Nakai, T. Vreven, K. Throssell, J. A. Montgomery, Jr., J. E. Peralta, F. Ogliaro, M. J. Bearpark, J. J. Heyd, E. N. Brothers, K. N. Kudin, V. N. Staroverov, T. A. Keith, R. Kobayashi, J. Normand, K. Raghavachari, A. P. Rendell, J. C. Burant, S. S. Iyengar, J. Tomasi, M. Cossi, J. M. Millam, M. Klene, C. Adamo, R. Cammi, J. W. Ochterski, R. L. Martin, K. Morokuma, O. Farkas, J. B. Foresman, and D. J. Fox, Gaussian, Inc., Wallingford CT, **2019**.

11 NBO 7.0. E. D. Glendening, J. K. Badenhoop, A. E. Reed, J. E. Carpenter, J. A. Bohmann, C. M. Morales, P. Karafiloglou, C. R. Landis, and F. Weinhold, Theoretical Chemistry Institute, University of Wisconsin, Madison, WI (2018).

12 Jmol: an open-source Java viewer for chemical structures in 3D. https://www.jmol.org/

7. Author Contributions

Dr. M.-E. Moret initiated and supervised the project. P.A. Benzan Lantigua performed the experiments and Dr. M. L. G. Sansores-Paredes performed the DFT calculations. Dr. M. Lutz oversaw the acquisition, solving and interpretation of the x-ray crystal structure. P.A. Benzan Lantigua and Dr. M. L. G. Sansores-Paredes wrote the manuscript with input from Dr. M.-E. Moret and with the contribution of Dr. M. Lutz. All authors approved the final manuscript.
